# Supplementary material for: In Vitro and In Silico Biological Studies of 4-Phenyl-2-quinolone (4-PQ) Derivatives as Anticancer Agents
Source: Molecules. 2023 Jan 5;28(2):555. doi: 10.3390/molecules28020555 (PMC9861105; doi:10.3390/molecules28020555)
Supplement: Supplementary file 1 [file molecules-28-00555-s001.zip › molecules-2104377-supplementary materials.pdf]

molecules

## Supplementary Materials

# In Vitro and In Silico Biological Studies of 4-Phenyl-2-Quinolone (4-PQ) Derivatives as Anticancer Agents

**Yi-Fong Chen [1,2,3], Bashir Lawal [4,5], Li-Jiau Huang [2,6], Sheng-Chu Kuo [2,6,7], Maryam Rachmawati Sumitra [1,3], Ntlotlang Mokgautsi [1,3], Hung-Yun Lin [1,8,9,10,11,*] and Hsu-Shan Huang [1,3,12,13,*]**

[1] Graduate Institute of Cancer Molecular Biology and Drug Discovery, College of Medical Science and Technology, Taipei Medical University, Taipei 11031, Taiwan

[2] Ph.D. Program for Cancer Biology and Drug Discovery, College of Medicine, China Medical University and Academia Sinica, Taichung 40402, Taiwan

[3] Ph.D. Program for Cancer Molecular Biology and Drug Discovery, College of Medical Science and Technology, Taipei Medical University and Academia Sinica, Taipei 11031, Taiwan

[4] UPMC Hillman Cancer Center, University of Pittsburgh, Pittsburgh, Pennsylvania, USA

[5] Department of Pathology, University of Pittsburgh, Pittsburgh, Pennsylvania, USA

[6] School of Pharmacy, China Medical University, Taichung 40402, Taiwan

[7] Chinese Medicinal Research and Development Center, China Medical University Hospital, Taichung 40402, Taiwan

[8] Cancer Center, Wan Fang Hospital, Taipei Medical University, Taipei 11031, Taiwan

[9] TMU Research Center of Cancer Translational Medicine, Taipei Medical University, Taipei 11031, Taiwan

[10] Traditional Herbal Medicine Research Center of Taipei Medical University Hospital, Taipei Medical University, Taipei 11031, Taiwan

[11] Pharmaceutical Research Institute, Albany College of Pharmacy and Health Sciences, Rensselaer, NY 12144, USA

[12] Graduate Institute of Medical Sciences, National Defense Medical Center, Taipei 11490, Taiwan

[13] Ph.D. Program in Drug Discovery and Development Industry, College of Pharmacy, Taipei Medical University, Taipei 11031, Taiwan

* **Correspondence: linhy@tmu.edu.tw (H.-Y.L.); huanghs99@tmu.edu.tw (H.-S.H.); Tel.: +886-2-2736-1661 (H.-Y.L. and H.-S.H.).**

The supplemental contents are as follows:

1. Characterization data of compounds benzoylacetates (**3b–e**)--- Page S2
2. Characterization data of compounds benzoylacetanilides (**5-18**)--- Page S2-S4
3. Characterization data of compounds 5-, 6-, 7-methoxy-substituted 4-phenyl-2-quinolones (**19–32**)---Page S4-S6
4. Meta-Pred Web Server prediction results of site of metabolism for compound **22**---Page S7-S9
5. Spectral copies of $^{1}$HNMR, $^{13}$C NMR, IR and Mass of the 4-PQ compounds **19-32**---Page S10-S53

## 1. *Characterization data of compounds benzoylacetates (**3b–e**)*

### 1.1. Ethyl 3-(3-methoxyphenyl)-3-oxopropanoate (**3b**)

Yield 83.7% from **1b** as a yellow liquid; IR (KBr) m ($cm^{-1}$): 1743.65, 1689.64 (C=O); $^1H$ NMR (200 MHz, DMSO-*d6*): δ 1.16 (t, *J* = 7.2 Hz, 3H, $–OCH_2CH_3$), 3.80 (s, 3H, $–OCH_3$), 4.06–4.20 (m, 4H, $–COCH_2CO–$ and $–OCH_2CH_3$), 7.19–7.25 (m, 1H, ArH), 7.40–7.57 (m, 3H, ArH).; $^{13}C$ NMR (50 MHz, DMSO-*d6*): δ 14.36, 46.12, 55.73, 61.05, 113.31, 120.18, 121.34, 130.38, 137.64, 159.91, 168.08, 193.67; LRMS (EI) m/z: 222.1 ($M^+$); HRMS (EI) m/z: calculated for $C_{12}H_{14}O_4$: 222.0892; found: 222.0898.

### 1.2. Ethyl 3-(4-methoxyphenyl)-3-oxopropanoate (**3c**)

Yield 76.9% from **1c** as a yellow liquid; IR (KBr) m ($cm^{-1}$): 1735.93, 1674.21 (C=O); $^1H$ NMR (200 MHz, DMSO-*d6*): δ 1.16 (t, *J* = 7.2 Hz, 3H, $–OCH_2CH_3$), 3.82 (s, 3H, $–OCH_3$), 4.05–4.15 (m, 4H, $–COCH_2CO–$ and $–OCH_2CH_3$), 7.03 (d, *J* = 8.9 Hz, 2H, ArH), 7.93 (d, *J* = 8.9 Hz, 2H, ArH); $^{13}C$ NMR (50 MHz, DMSO-*d6*): δ 14.36, 45.71, 55.95, 60.97, 114.40 (2C), 129.24, 131.24 (2C), 164.04, 168.25, 192.06; LRMS (EI) m/z: 222.1 ($M^+$); HRMS (EI) m/z: calculated for $C_{12}H_{14}O_4$: 222.0892; found: 222.0899.

### 1.3. Ethyl 3-(2,4-dimethoxyphenyl)-3-oxopropanoate (**3d**)

Yield 73.9% from **1d** as a yellow liquid; IR (KBr) m ($cm^{-1}$): 1735.93, 1666.50 (C=O); $^1H$ NMR (200 MHz, DMSO-*d6*): δ 1.16 (t, *J* = 7.2 Hz, 3H, $-O-CH_2CH_3$), 3.83-3.84 (m, 8H, 2 × $–OCH_3$ and $–COCH_2CO–$), 4.08 (q, *J* = 7.2 Hz, 2H, $–OCH_2CH_3$), 6.58–6.62 (m, 2H, ArH), 7.75 (d, *J* = 9.4 Hz, 1H, ArH); $^{13}C$ NMR (50 MHz, DMSO-*d6*): δ 14.39, 50.42, 56.03 (2C), 60.64, 98.59, 106.83, 119.11, 132.61, 161.57, 165.47, 168.46, 190.91; LRMS (EI) m/z: 252.1 ($M^+$); HRMS (EI) m/z: calculated for $C_{13}H_{16}O_5$: 252.0998; found: 252.0991.

### 1.4. Ethyl 3-(2,5-dimethoxyphenyl)-3-oxopropanoate (**3e**)

Yield 70.0% from **1e** as a yellow liquid; IR (KBr) m ($cm^{-1}$): 1735.93, 1674.21 (C=O); $^1H$ NMR (200 MHz, DMSO-*d6*): δ 1.16 (t, *J* = 7.1 Hz, 3H, $–OCH_2CH_3$), 3.73 (s, 3H, $–OCH_3$), 3.80 (s, 3H, $–OCH_3$), 3.91 (s, 2H, $–COCH_2CO–$), 4.08 (q, *J* = 7.1 Hz, 2H, $–OCH_2CH_3$), 7.12–7.15 (m, 2H, ArH), 7.22–7.24 (m, 1H, ArH); $^{13}C$ NMR (50 MHz, DMSO-*d6*): δ 14.38, 50.38, 55.90, 56.42, 60.75, 113.94, 114.51, 121.47, 126.35, 153.40, 153.81, 168.12, 192.75; LRMS (EI) m/z: 252.1 ($M^+$); HRMS (EI) m/z: calculated for $C_{13}H_{16}O_5$: 252.0998; found: 252.0991.

## 2. *Characterization data of compounds benzoylacetanilides (**5-18**)*

### 2.1. 3-Oxo-*N*,3-diphenylpropanamide (**5**)

Compound **5** was obtained by the reaction of ethyl benzoyl acetate (**3a**) and aniline (**4a**). TLC monitored (*n*-hexane/ethyl acetate = 5/1; Rf = 0.15). Yield 37.6% as a white solid; Mp = 106–109 °C; IR (KBr) m ($cm^{-1}$): 1662, 1695 (C=O); $^1H$ NMR (400 MHz, $CDCl_3$-*d1*): δ 4.08 (s, 2H, $–CH_2–$), 7.10 (t, *J* = 7.2 Hz, 1H, ArH), 7.30 (t, *J* = 7.6 Hz, 2H, ArH), 7.48 (t, *J* = 7.6 Hz, 2H, ArH), 7.56 (d, *J* = 8.0 Hz, 2H, ArH), 7.61 (t, *J* = 7.6 Hz, 1H, ArH), 7.99 (d, *J* = 7.6 Hz, 1H, ArH), 9.32 (s, 1H, NH); $^{13}C$ NMR (100 MHz, $CDCl_3$-*d1*): δ 45.58 ($–CH_2–$), 120.17 (2C), 124.53, 128.56 (2C), 128.91 (2C), 128.95 (2C), 134.28, 135.99, 137.54, 163.89, 196.32.

### 2.2. 3-(4-Methoxyphenyl)-3-oxo-*N*-phenylpropanamide (**6**)

Compound **6** was obtained by the reaction of ethyl 3-(4-methoxyphenyl)-3-oxopropanoate (**3c**) and aniline (**4a**). TLC monitored (*n*-hexane/ethyl acetate = 5/1; Rf = 0.13). Yield 39.2% as a white solid; Mp = 120–122 °C; IR (KBr) m ($cm^{-1}$): 1593, 1680 (C=O); $^1H$ NMR (400 MHz, $CDCl_3$-*d1*): δ 3.85 (s, 3H, $–OCH_3$), 4.02 (s, 2H, $–CH_2–$), 6.93 (d, *J* = 8.8 Hz, 2H, ArH), 7.08 (t, *J* = 7.2 Hz, 1H, ArH), 7.29 (t, *J* = 8.0 Hz, 2H, ArH), 7.56 (d, *J* = 8.0 Hz, 2H, ArH), 7.98 (d, *J* = 8.8 Hz, 2H, ArH), 9.40 (s, 1H, NH); $^{13}C$ NMR (100 MHz, $CDCl_3$-*d1*): δ 45.19 ($–CH_2–$), 55.55 ($–OCH_3$), 114.09 (2C), 120.10 (2C), 124.39, 128.90 (2C), 128.99, 131.06 (2C), 137.64, 164.11, 164.47, 194.68.

### 2.3. *N*-(4-Methoxyphenyl)-3-oxo-3-phenylpropanamide (**7**)

Compound **7** was obtained by the reaction of ethyl benzoyl acetate (**3a**) and *p*-anisidine (**4b**). TLC monitored (*n*-hexane/ethyl acetate = 1/1; Rf = 0.75). Yield 68.2% as a light-yellow solid; Mp = 107–108 °C; IR (KBr) m ($cm^{-1}$): 1697, 1741 (C=O); $^1H$ NMR (400 MHz, $CDCl_3$-*d1*): δ 3.75 (s, 3H, $–OCH_3$), 4.06 (s, 2H, $–CH_2–$), 6.82 (d, *J* = 9.2 Hz, 2H, ArH), 7.43–7.49 (m, 4H, ArH), 7.60 (t, *J* = 7.6 Hz, 1H, ArH), 7.99 (d, *J* = 7.6 Hz, 1H, ArH), 9.16 (s, 1H, NH); $^{13}C$ NMR (100 MHz, $CDCl_3$-*d1*): δ 45.46 ($–CH_2–$), 55.40 ($–OCH_3$), 114.07 (2C), 121.95 (2C), 128.54 (2C), 128.88 (2C), 130.68, 134.21, 136.04, 156.51, 163.69, 196.37.

### 2.4. 3-(3-Methoxyphenyl)-*N*-(4-methoxyphenyl)-3-oxopropanamide (**8**)

Compound **8** was obtained by the reaction of ethyl 3-(3-methoxyphenyl)-3-oxopropanoate (**3b**) and *p*-anisidine (**4b**). TLC monitored (*n*-hexane/ethyl acetate = 2/1; Rf = 0.5). Yield 33.5% as a brown liquid; IR (KBr) m ($cm^{-1}$): 1581,

1598 (C=O); $^1$H NMR (400 MHz, $CDCl_3$-$d_1$): δ 3.75 (s, 3H, –$OCH_3$), 3.84 (s, 3H, –$OCH_3$), 4.06 (s, 2H, –$CH_2$–), 6.72–6.73 (m, 2H, ArH), 6.82–6.88 (m, 2H, ArH), 7.15–7.17 (m, 1H, ArH), 7.38–7.60 (m, 3H, ArH), 9.16 (s, 1H, NH); $^{13}$C NMR (100 MHz, $CDCl_3$-$d_1$): δ 45.46 (–$CH_2$–), 55.17 (–$OCH_3$), 55.45 (–$OCH_3$), 114.43 (2C), 114.79, 120.90, 121.26, 121.92 (2C), 129.92, 130.63, 138.11, 156.35, 159.97, 163.47, 194.79.

2.5. *N*,3-Bis(4-methoxyphenyl)-3-oxopropanamide (**9**)

Compound **9** was obtained by the reaction of ethyl 3-(4-methoxyphenyl)-3-oxopropanoate (**3c**) and *p*-anisidine (**4b**). TLC monitored (*n*-hexane/ethyl acetate = 5/1; Rf = 0.1). Yield 68.7% as a white solid; Mp = 160–162 °C; IR (KBr) m ($cm^{-1}$): 1647, 1680 (C=O); $^1$H NMR (400 MHz, $CDCl_3$-$d_1$): δ 3.76 (s, 3H, –$OCH_3$), 3.87 (s, 3H, –$OCH_3$), 4.01 (s, 2H, –$CH_2$–), 6.84 (d, *J* = 8.8 Hz, 2H, ArH), 6.95 (d, *J* = 8.8 Hz, 2H, ArH), 7.45 (d, *J* = 8.4 Hz, 1H, ArH), 7.99 (d, *J* = 8.8 Hz, 1H, ArH), 9.24 (s, 1H, NH); $^{13}$C NMR (100 MHz, $CDCl_3$-$d_1$): δ 44.95 (–$CH_2$–), 55.41 (–$OCH_3$), 55.55 (–$OCH_3$), 114.05 (2C), 114.09 (2C), 121.86 (2C), 129.04, 130.76, 131.05 (2C), 156.43, 163.80, 164.47, 194.86.

2.6. *N*-(3,5-Dimethoxyphenyl)-3-oxo-3-phenylpropanamide (**10**)

Compound **10** was obtained by the reaction of ethyl benzoyl acetate (**3a**) and 3,5-dimethoxyaniline (**4c**). TLC monitored (*n*-hexane/ethyl acetate = 1/1; Rf = 0.68). Yield 73.4% as a white solid; Mp = 114–115 °C; IR (KBr) m ($cm^{-1}$): 1654, 1697 (C=O); $^1$H NMR (400 MHz, DMSO-$d_6$): δ 3.71 (s, 6H, 2 × –$OCH_3$), 4.13 (s, 2H, –$CH_2$–), 6.23 (s, 1H, ArH), 6.83 (s, 2H, ArH), 7.57 (d, *J* = 8.0 Hz, 2H, ArH), 7.67 (t, *J* = 7.2 Hz, 1H, ArH), 8.00 (d, *J* = 7.6 Hz, 2H, ArH), 10.17 (s, 1H, NH); $^{13}$C NMR (100 MHz, DMSO-$d_6$): δ 48.55 (–$CH_2$–), 55.47 (–$OCH_3$), 55.54 (–$OCH_3$), 95.85, 97.72 (2C), 128.78 (2C), 129.21 (2C), 134.00, 136.65, 141.02, 160.93, 160.98, 165.85, 194.93.

2.7. *N*-(3,5-Dimethoxyphenyl)-3-(3-methoxyphenyl)-3-oxopropanamide (**11**)

Compound **11** was obtained by the reaction of ethyl 3-(3-methoxyphenyl)-3-oxopropanoate (**3b**) and 3,5-dimethoxyaniline (**4c**). TLC monitored (*n*-hexane/ethyl acetate = 1/1; Rf = 0.6). Yield 7.8% as a white solid; Mp = 96–98 °C; IR (KBr) m ($cm^{-1}$): 1664, 1693 (C=O); $^1$H NMR (400 MHz, $CDCl_3$-$d_1$): δ 3.76 (s, 6H, 2 × –$OCH_3$), 3.85 (s, 3H, –$OCH_3$), 4.07 (s, 2H, –$CH_2$–), 6.23 (s, 1H, ArH), 6.81 (s, 2H, ArH), 7.16 (dd, *J* = 8.0, 2.0 Hz, 1H, ArH), 7.40 (t, *J* = 8.0 Hz, 1H, ArH), 7.51 (s, 1H, ArH), 7.59 (d, *J* = 7.6 Hz, 1H, ArH), 9.21 (s, 1H, NH); $^{13}$C NMR (100 MHz, $CDCl_3$-$d_1$): δ 45.70 (–$CH_2$–), 55.35 (2C, 2 × –$OCH_3$), 55.44 (–$OCH_3$), 97.03, 98.33 (2C), 112.52, 120.91, 121.25, 129.92, 137.27, 139.22, 159.97, 160.97 (2C), 163.75, 196.18.

2.8. *N*-(3,5-Dimethoxyphenyl)-3-(4-methoxyphenyl)-3-oxopropanamide (**12**)

Compound **12** was obtained by the reaction of ethyl 3-(4-methoxyphenyl)-3-oxopropanoate (**3c**) and 3,5-dimethoxyaniline (**4c**). TLC monitored (*n*-hexane/ethyl acetate = 5/1; Rf = 0.13). Yield 69.0% as a light-yellow solid; Mp = 109–111 °C; IR (KBr) m ($cm^{-1}$): 1600, 1678 (C=O); $^1$H NMR (400 MHz, $CDCl_3$-$d_1$): δ 3.75 (s, 6H, 2 × –$OCH_3$), 3.86 (s, 3H, –$OCH_3$), 4.01 (s, 2H, –$CH_2$–), 6.22 (s, 1H, ArH), 6.80 (s, 2H, ArH), 6.94 (d, *J* = 8.8 Hz, 2H, ArH), 7.98 (d, *J* = 8.8 Hz, 2H, ArH), 9.35 (s, 1H, NH); $^{13}$C NMR (100 MHz, $CDCl_3$-$d_1$): δ 45.16 (–$CH_2$–), 55.35 (2C, 2 × –$OCH_3$), 55.56 (–$OCH_3$), 96.99, 98.26 (2C), 114.11 (2C), 128.95, 131.06 (2C), 139.30, 160.95 (2C), 164.01, 164.52, 194.72.

2.9. 3-(2,4-Dimethoxyphenyl)-*N*-(3,5-dimethoxyphenyl)-3-oxopropanamide (**13**)

Compound **13** was obtained by the reaction of ethyl 3-(2,4-dimethoxyphenyl)-3-oxopropanoate (**3d**) and 3,5-dimethoxyaniline (**4c**). TLC monitored (*n*-hexane/ethyl acetate = 1/1; Rf = 0.25). Yield 28.3% as a gray solid; Mp = 136–138 °C; IR (KBr) m ($cm^{-1}$): 1595, 1676 (C=O); $^1$H NMR (400 MHz, $CDCl_3$-$d_1$): δ 3.76 (s, 6H, 2 × –$OCH_3$), 3.85 (s, 3H, –$OCH_3$), 3.90 (s, 3H, –$OCH_3$), 4.07 (s, 2H, –$CH_2$–), 6.21 (s, 1H, ArH), 6.44 (d, *J* = 1.2 Hz, 1H, ArH), 6.54 (dd, *J* = 8.8, 1.6 Hz, 1H, ArH), 6.82 (s, 2H, ArH), 7.83 (d, *J* = 8.8 Hz, 1H, ArH), 9.37 (s, 1H, NH); $^{13}$C NMR (100 MHz, $CDCl_3$-$d_1$): δ 49.85 (–$CH_2$–), 55.33 (2C, 2 × –$OCH_3$), 55.58 (2C, 2 × –$OCH_3$), 96.76, 98.22 (3C), 105.71, 120.03, 133.02, 139.57, 160.91 (2C), 161.27, 165.10, 165.55, 195.91.

2.10. 3-(2,5-Dimethoxyphenyl)-*N*-(3,5-dimethoxyphenyl)-3-oxopropanamide (**14**)

Compound **14** was obtained by the reaction of ethyl 3-(2,5-dimethoxyphenyl)-3-oxopropanoate (**3e**) and 3,5-dimethoxyaniline (**4c**). TLC monitored (*n*-hexane/ethyl acetate = 1/1; Rf = 0.5). Yield 32.3% as a light-yellow solid; Mp = 125–127 °C; IR (KBr) m ($cm^{-1}$): 1654, 1674 (C=O); $^1$H NMR (400 MHz, $CDCl_3$-$d_1$): δ 3.76 (s, 6H, 2 × –$OCH_3$), 3.78 (s, 3H, –$OCH_3$), 3.87 (s, 3H, –$OCH_3$), 4.11 (s, 2H, –$CH_2$–), 6.22 (s, 1H, ArH), 6.81 (s, 2H, ArH), 6.91 (d, *J* = 9.2 Hz, 1H, ArH), 7.07 (dd, *J* = 9.2, 3.2 Hz, 1H, ArH), 7.27 (d, *J* = 3.2 Hz, 1H, ArH), 9.16 (s, 1H, NH); $^{13}$C NMR (100 MHz, $CDCl_3$-$d_1$): δ 50.24 (–$CH_2$–), 55.35 (2C, 2 × –$OCH_3$), 55.80 (–$OCH_3$), 56.02 (–$OCH_3$), 97.67 (2C), 96.81, 98.23 (2C), 113.18, 113.76, 121.77, 127.09, 139.45, 153.48, 153.54, 160.95 (2C), 164.55, 197.80.

2.11. 3-Oxo-3-phenyl-*N*-(3,4,5-trimethoxyphenyl)propanamide (**15**)

Compound **15** was obtained by the reaction of ethyl benzoyl acetate (**3a**) and 3,4,5-trimethoxyaniline (**4d**). TLC monitored (*n*-hexane/ethyl acetate = 1/1; Rf = 0.45). Yield 87.8% as a white solid; Mp = 129–132 °C; IR (KBr) m ($cm^{-1}$):

1672, 1687 (C=O); $^{1}$H NMR (400 MHz, $CDCl_3$-$d_1$): δ 3.79 (s, 3H, –$OCH_3$), 3.83 (s, 6H, 2 × –$OCH_3$), 4.09 (s, 2H, –$CH_2$–), 6.87 (s, 2H, ArH), 7.51 (t, *J* = 7.6 Hz, 2H, ArH), 7.64 (t, *J* = 7.6 Hz, 1H, ArH), 8.02 (d, *J* = 7.6 Hz, 2H, ArH), 9.23 (s, 1H, NH); $^{13}$C NMR (100 MHz, $CDCl_3$-$d_1$): δ 45.37 (–$CH_2$–), 56.09 (2C, 2 × –$OCH_3$), 60.90 (–$OCH_3$), 97.77 (2C), 128.56 (2C), 128.97 (2C), 133.61, 134.43 (2C), 135.94, 153.26 (2C), 163.54, 196.62.

2.12. 3-(4-Methoxyphenyl)-3-oxo-*N*-(3,4,5-trimethoxyphenyl)propanamide (**16**)

Compound **16** was obtained by the reaction of ethyl 3-(4-methoxyphenyl)-3-oxopropanoate (**3c**) and 3,4,5-trimethoxyaniline (**4d**). TLC monitored (*n*-hexane/ethyl acetate = 1/1; Rf = 0.18). Yield 55.0% as a white solid; Mp = 125–128 °C; IR (KBr) m (cm$^{-1}$): 1664, 1685 (C=O); $^{1}$H NMR (400 MHz, $CDCl_3$-$d_1$): δ 3.78 (s, 3H, –$OCH_3$), 3.81 (s, 6H, 2 × –$OCH_3$), 3.86 (s, 3H, –$OCH_3$), 4.01 (s, 2H, –$CH_2$–), 6.86 (s, 2H, ArH), 6.94 (d, *J* = 8.8 Hz, 2H, ArH), 7.98 (d, *J* = 8.8 Hz, 2H, ArH), 9.34 (s, 1H, NH); $^{13}$C NMR (100 MHz, $CDCl_3$-$d_1$): δ 45.12 (–$CH_2$–), 55.56 (–$OCH_3$), 56.04 (2C, 2 × –$OCH_3$), 60.88 (–$OCH_3$), 97.66 (2C), 114.13 (2C), 128.90, 131.06 (2C), 133.78, 134.65, 153.20 (2C), 163.98, 164.56, 194.80.

2.13. 3-(2,4-Dimethoxyphenyl)-3-oxo-*N*-(3,4,5-trimethoxyphenyl)propanamide (**17**)

Compound **17** was obtained by the reaction of ethyl 3-(2,4-dimethoxyphenyl)-3-oxopropanoate (**3d**) and 3,4,5-trimethoxyaniline (**4d**). TLC monitored (*n*-hexane/ethyl acetate = 1/1; Rf = 0.3). Yield 50.0% as a gray solid; Mp = 148–151 °C; IR (KBr) m (cm$^{-1}$): 1645, 1660 (C=O); $^{1}$H NMR (400 MHz, $CDCl_3$-$d_1$): δ 3.79 (s, 3H, –$OCH_3$), 3.83 (s, 6H, 2 × –$OCH_3$), 3.85 (s, 3H, –$OCH_3$), 3.90 (s, 3H, –$OCH_3$), 4.08 (s, 2H, –$CH_2$–), 6.44 (d, *J* = 1.6 Hz, 1H, ArH), 6.55 (dd, *J* = 8.8, 2.0 Hz, 1H, ArH), 6.88 (s, 2H, ArH), 7.83 (d, *J* = 8.8 Hz, 1H, ArH), 9.41 (s, 1H, NH); $^{13}$C NMR (100 MHz, $CDCl_3$-$d_1$): δ 49.53 (–$CH_2$–), 55.61 (2C, 2 × –$OCH_3$), 56.06 (2C, 2 × –$OCH_3$), 60.89 (–$OCH_3$), 97.67 (2C), 98.30, 105.73, 120.04, 133.01, 133.98, 134.50, 153.19 (2C), 161.32, 164.93, 165.63, 196.26.

2.14. 3-(2,5-Dimethoxyphenyl)-3-oxo-*N*-(3,4,5-trimethoxyphenyl)propanamide (**18**)

Compound **18** was obtained by the reaction of ethyl 3-(2,5-dimethoxyphenyl)-3-oxopropanoate (**3e**) and 3,4,5-trimethoxyaniline (**4d**). TLC monitored (*n*-hexane/ethyl acetate = 1/1; Rf = 0.3). Yield 20.3% as a white solid; Mp = 166–169 °C; IR (KBr) m (cm$^{-1}$): 1645, 1664 (C=O); $^{1}$H NMR (400 MHz, $CDCl_3$-$d_1$): δ 3.78 (s, 3H, –$OCH_3$), 3.79 (s, 3H, –$OCH_3$), 3.83 (s, 6H, 2 × –$OCH_3$), 3.88 (s, 3H, –$OCH_3$), 4.12 (s, 2H, –$CH_2$–), 6.87 (s, 2H, ArH), 6.92 (d, *J* = 8.8 Hz, 1H, ArH), 7.08 (dd, *J* = 8.8, 3.2 Hz, 1H, ArH), 7.27 (d, *J* = 2.8 Hz, 1H, ArH), 9.19 (s, 1H, NH); $^{13}$C NMR (100 MHz, $CDCl_3$-$d_1$): δ 50.01 (–$CH_2$–), 55.80 (–$OCH_3$), 56.06 (3C, 3 × –$OCH_3$), 60.90 (–$OCH_3$), 97.67 (2C), 113.21, 113.79, 121.77, 127.07, 133.88, 134.58, 153.22 (2C), 153.49, 153.57, 164.48, 198.08.

## *3. Characterization data of compounds 5-, 6-, 7-methoxy-substituted 4-phenyl-2-quinolones (**19–32**)*

3.1. 4-Phenylquinolin-2(1*H*)-one (**19**)

Compound **19** (0.74 g, 3.35 mmole) was obtained by cyclization of compound **5** (1.26 g, 5.27 mmole). TLC monitored (*n*-hexane/ethyl acetate = 1/1; Rf = 0.3). Yield 63.6% was recrystallized by ethanol as a colorless rectangular crystal; Mp = 263–265 °C; IR (KBr) m (cm$^{-1}$): 1664 (C=O); $^{1}$H NMR (400 MHz, DMSO-$d_6$): δ 6.39 (s, 1H, H–3), 7.12 (t, *J* = 7.6 Hz, 1H, H–6), 7.36 (d, *J* = 8.0 Hz, 1H, H–5), 7.40 (d, *J* = 8.4 Hz, 1H, H–8), 7.44–7.46 (m, 2H, H–7,4′), 7.49–7.53 (m, 4H, H–2′,3′,5′,6′), 11.90 (s, 1H, NH); $^{13}$C NMR (100 MHz, DMSO-$d_6$): δ 116.20 (C–3), 118.75 (C–4a), 121.61 (C–8), 122.26 (C–6), 126.52 (C–7), 129.05 (2C, C–3′,5′), 129.08 (2C, C–2′,6′), 129.15 (C–4′), 130.95 (C–5), 137.09 (C–1′), 139.69 (C–8a), 151.90 (C–4), 161.74 (C=O); HRMS (ESI) m/z calcd for $C_{15}H_{11}NO^+$ [M]$^+$: 221.0841, found [M+H]$^+$: 222.0911.

3.2. 4-(4-Methoxyphenyl)quinolin-2(1*H*)-one (**20**)

Compound **20** (0.45 g, 1.79 mmole) was obtained by cyclization of compound **6** (1.25 g, 4.64 mmole). TLC monitored (*n*-hexane/ethyl acetate = 1/1; Rf = 0.2). Yield 38.6% was recrystallized by ethanol as a pale yellow needle crystal; Mp = 230–232 °C; IR (KBr) m (cm$^{-1}$): 1672 (C=O); $^{1}$H NMR (400 MHz, DMSO-$d_6$): δ 3.83 (s, 3H, 4′–$OCH_3$), 6.36 (s, 1H, H–3), 7.08 (d, *J* = 8.4 Hz, 2H, H–3′,5′), 7.13 (t, *J* = 7.6 Hz, 1H, H–6), 7.37–7.44 (m, 4H, H–5,8,2′,6′), 7.51 (t, *J* = 8.0 Hz, 1H, H–7), 11.84 (s, 1H, NH); $^{13}$C NMR (100 MHz, DMSO-$d_6$): δ 55.65 (4′–$OCH_3$), 114.53 (2C, C–3′,5′), 116.19 (C–3), 118.92 (C–4a), 121.28 (C–8), 122.19 (C–6), 126.60 (C–7), 129.23 (C–5), 130.47 (2C, C–2′,6′), 130.86 (C–1′), 139.73 (C–8a), 151.60 (C–4), 160.06 (C–4′), 161.81 (C=O); HRMS (ESI) m/z calcd for $C_{16}H_{13}NO_2^+$ [M]$^+$: 251.0946, found [M+H]$^+$: 252.1016.

3.3. 6-Methoxy-4-phenylquinolin-2(1*H*)-one (**21**)

Compound **21** (0.48 g, 1.91 mmole) was obtained by cyclization of compound **7** (1.63 g, 6.06 mmole). TLC monitored (*n*-hexane/ethyl acetate = 1/1; Rf = 0.25). Yield 31.5% was recrystallized by ethanol/ethyl acetate as a white cotton crystal; Mp = 216–218 °C; IR (KBr) m (cm$^{-1}$): 1654 (C=O); $^{1}$H NMR (400 MHz, DMSO-$d_6$): δ 3.64 (s, 3H, 6–$OCH_3$), 6.39 (s, 1H, H–3), 6.82 (d, *J* = 2.4 Hz, 1H, H–5), 7.22 (d, *J* = 8.8, 2.4 Hz, 1H, H–7), 7.36 (d, *J* = 8.8 Hz, 1H, H–8), 7.48–7.55 (m, 5H, H–2′,3′,4′,5′,6′), 11.81 (s, 1H, NH); $^{13}$C NMR (100 MHz, DMSO-$d_6$): δ 55.67 (6–$OCH_3$), 108.62 (C–5), 117.52 (C–8), 119.28 (C–4a), 119.60 (C–7), 122.08 (C–3), 128.98 (2C, C–3′,5′), 129.17 (2C, C–2′,6′), 129.24 (C–4′), 134.16 (C–8a), 137.12 (C–1′),

151.35 (C–4), 154.45 (C–6), 161.35 (C=O); HRMS (ESI) m/z calcd for $C_{16}H_{13}NO_2^+$ [M]$^+$: 251.0946, found [M+H]$^+$: 252.1014.

3.4. 6-Methoxy-4-(3-methoxyphenyl)quinolin-2(1*H*)-one (**22**)

Compound **22** (0.15 g, 0.53 mmole) was obtained by cyclization of compound **8** (0.83 g, 2.77 mmole). TLC monitored (ethyl acetate; Rf = 0.2). Yield 19.1% was purified by column chromatography (silica gel, ethyl acetate) as a brown solid; Mp = 286–288 °C; IR (KBr) m (cm$^{-1}$): 1598 (C=O); $^1$H NMR (400 MHz, DMSO-*d*$_6$): δ 3.84 (s, 3H, 6–OCH$_3$), 3.86 (s, 3H, 3′–OCH$_3$), 6.38 (s, 1H, H–3), 7.12 (d, *J* = 7.6 Hz, 1H, H–6′), 7.32 (dd, *J* = 8.8, 2.4 Hz, 1H, H–7), 7.36–7.39 (m, 2H, H–2′,4′), 7.48 (t, *J* = 8.0 Hz, 1H, H–5′), 7.51 (d, *J* = 2.4 Hz, 1H, H–5), 7.76 (d, *J* = 8.8 Hz, 1H, H–8), 11.76 (s, 1H, NH); $^{13}$C NMR (100 MHz, DMSO-*d*$_6$): δ 55.73 (6–OCH$_3$), 55.77 (3′–OCH$_3$), 104.27 (C–5), 106.52 (C–3), 113.11 (C–2′), 116.31 (C–6′), 119.92 (C–4′), 121.18 (C–8), 122.63 (C–7), 126.20 (C–4a), 130.57 (C–1′), 149.47 (C–4), 156.08 (2C, C–6,8a), 159.94 (2C, C=O and C–3′); HRMS (ESI) m/z calcd for $C_{17}H_{15}NO_3^+$ [M]$^+$: 281.1052, found [M+H]$^+$: 282.1122.

3.5. 6-Methoxy-4-(4-methoxyphenyl)quinolin-2(1*H*)-one (**23**)

Compound **23** (0.32 g, 1.14 mmole) was obtained by cyclization of compound **9** (1.60 g, 5.35 mmole). TLC monitored (*n*-hexane/ethyl acetate = 1/1; Rf = 0.15). Yield 21.3% was recrystallized by ethanol/ethyl acetate as a light-yellow solid; Mp = 275–277 °C; IR (KBr) m (cm$^{-1}$): 1649 (C=O); $^1$H NMR (400 MHz, DMSO-*d*$_6$): δ 3.66 (s, 3H, 6–OCH$_3$), 3.83 (s, 3H, 4′–OCH$_3$), 6.35 (s, 1H, H–3), 6.89 (d, *J* = 2.4 Hz, 1H, H–5), 7.09 (d, *J* = 8.4 Hz, 2H, H–3′,5′), 7.21 (d, *J* = 8.8, 2.4 Hz, 1H, H–7), 7.35 (d, *J* = 8.8 Hz, 1H, H–8), 7.44 (d, *J* = 8.4 Hz, 2H, H–2′,6′), 11.71 (s, 1H, NH); $^{13}$C NMR (100 MHz, DMSO-*d*$_6$): δ 55.62 (4′–OCH$_3$), 55.69 (6–OCH$_3$), 108.67 (C–5), 114.59 (2C, C–3′,5′), 117.48 (C–8), 119.45 (C–4a), 119.52 (C–7), 121.78 (C–3), 129.28 (C–1′), 130.39 (2C, C–2′,6′), 134.19 (C–8a), 151.04 (C–4), 154.41 (C–6), 160.06 (C–4′), 161.41 (C=O); HRMS (ESI) m/z calcd for $C_{17}H_{15}NO_3^+$ [M]$^+$: 281.1052, found [M+H]$^+$: 282.1121.

3.6. 5,7-Dimethoxy-4-phenylquinolin-2(1*H*)-one (**24**)

Compound **24** (0.63 g, 2.24 mmole) was obtained by cyclization of compound **10** (1.53 g, 5.12 mmole). TLC monitored (*n*-hexane/ethyl acetate = 1/1; Rf = 0.1). Yield 43.8% was recrystallized by ethanol/ethyl acetate as a white cotton crystal; Mp = 258–259 °C; IR (KBr) m (cm$^{-1}$): 1654 (C=O); $^1$H NMR (400 MHz, DMSO-*d*$_6$): δ 3.34 (s, 3H, 5–OCH$_3$), 3.80 (s, 3H, 7–OCH$_3$), 5.92 (s, 1H, H–3), 6.25 (d, *J* = 1.6 Hz, 1H, H–6), 6.54 (d, *J* = 1.6 Hz, 1H, H–8), 7.20–7.23 (m, 2H, H–2′,6′), 7.33–7.35 (m, 3H, H–3′,4′,5′), 11.69 (s, 1H, NH); $^{13}$C NMR (100 MHz, DMSO-*d*$_6$): δ 55.67 (5–OCH$_3$), 55.77 (7–OCH$_3$), 91.68 (C–8), 94.30 (C–6), 103.70 (C–4a), 119.41 (C–3), 127.35 (C–4′), 127.48 (2C, C–3′,5′), 127.60 (2C, C–2′,6′), 141.90 (C–1′), 142.69 (C–8a), 151.46 (C–4), 158.40 (C–5), 161.65 (C=O), 162.35 (C–7); HRMS (ESI) m/z calcd for $C_{17}H_{15}NO_3^+$ [M]$^+$: 281.1052, found [M+H]$^+$: 282.1121.

3.7. 5,7-Dimethoxy-4-(3-methoxyphenyl)quinolin-2(1*H*)-one (**25**)

Compound **25** (0.16 g, 0.51 mmole) was obtained by cyclization of compound **11** (0.40 g, 1.22 mmole). TLC monitored (*n*-hexane/ethyl acetate = 1/1; Rf = 0.05). Yield 41.8% was recrystallized by ethyl acetate as a light-brown solid; Mp = 242–244 °C; IR (KBr) m (cm$^{-1}$): 1654 (C=O); $^1$H NMR (400 MHz, DMSO-*d*$_6$): δ 3.37 (s, 3H, 5–OCH$_3$), 3.75 (s, 3H, 3′–OCH$_3$), 3.80 (s, 3H, 7–OCH$_3$), 5.94 (s, 1H, H–3), 6.25 (s, 1H, H–6), 6.53 (s, 1H, H–8), 6.77–6.79 (m, 2H, H–2′,4′), 6.90 (d, *J* = 8.0 Hz, 1H, H–6′), 7.25 (t, *J* = 8.0 Hz, 1H, H–5′), 11.69 (s, 1H, NH); $^{13}$C NMR (100 MHz, DMSO-*d*$_6$): δ 55.45 (3′–OCH$_3$), 55.76 (2C, 5,7–OCH$_3$), 91.66 (C–8), 94.35 (C–6), 103.71 (C–4a), 113.06 (C–4′), 113.16 (C–6′), 119.31 (C–3), 120.16 (C–2′), 128.53 (C–5′), 142.68 (C–8a), 143.29 (C–1′), 151.21 (C–4), 158.40 (C–5), 158.65 (C–3′), 161.65 (C=O), 162.34 (C–7); HRMS (ESI) m/z calcd for $C_{18}H_{17}NO_4^+$ [M]$^+$: 311.1158, found [M+H]$^+$: 312.1227.

3.8. 5,7-Dimethoxy-4-(4-methoxyphenyl)quinolin-2(1*H*)-one (**26**)

Compound **26** (0.35 g, 1.12 mmole) was obtained by cyclization of compound **12** (2.47 g, 7.50 mmole). TLC monitored (*n*-hexane/ethyl acetate = 1/1; Rf = 0.1). Yield 14.9% was recrystallized by ethanol as a light-yellow solid; Mp = 287–289 °C; IR (KBr) m (cm$^{-1}$): 1660 (C=O); $^1$H NMR (400 MHz, DMSO-*d*$_6$): δ 3.40 (s, 3H, 5–OCH$_3$), 3.79 (s, 3H, 4′–OCH$_3$), 3.80 (s, 3H, 7–OCH$_3$), 5.91 (s, 1H, H–3), 6.26 (d, *J* = 2.0 Hz, 1H, H–6), 6.53 (d, *J* = 1.6 Hz, 1H, H–8), 6.90 (d, *J* = 8.4 Hz, 2H, H–3′,5′), 7.16 (d, *J* = 8.4 Hz, 2H, H–2′,6′), 11.65 (s, 1H, NH); $^{13}$C NMR (100 MHz, DMSO-*d*$_6$): δ 55.45 (4′–OCH$_3$), 55.76 (2C, 5,7–OCH$_3$), 91.68 (C–8), 94.30 (C–6), 103.82 (C–4a), 112.88 (2C, C–3′,5′), 119.48 (C–3), 129.04 (2C, C–2′,6′), 134.11 (C–1′), 142.74 (C–8a), 151.28 (C–4), 158.52 (C–5), 158.82 (C–4′), 161.71 (C=O), 162.25 (C–7); HRMS (ESI) m/z calcd for $C_{18}H_{17}NO_4^+$ [M]$^+$: 311.1158, found [M+H]$^+$: 312.1226.

3.9. 4-(2,4-Dimethoxyphenyl)-5,7-dimethoxyquinolin-2(1*H*)-one (**27**)

Compound **27** (0.26 g, 0.76 mmole) was obtained by cyclization of compound **13** (0.46 g, 1.28 mmole). TLC monitored (ethyl acetate; Rf = 0.4). Yield 59.4% was recrystallized by ethanol/ethyl acetate as a brown solid; Mp = 261–263 °C; IR (KBr) m (cm$^{-1}$): 1647 (C=O); $^1$H NMR (400 MHz, DMSO-*d*$_6$): δ 3.38 (s, 3H, 5–OCH$_3$), 3.62 (s, 3H, 2′–OCH$_3$), 3.78 (s, 3H, 7–OCH$_3$), 3.79 (s, 3H, 4′–OCH$_3$), 5.86 (s, 1H, H–3), 6.21 (d, *J* = 1.6 Hz, 1H, H–6), 6.49–6.52 (m, 2H, H–5′,8), 6.54 (s,

1H, H–3′), 6.99 (d, *J* = 8.4 Hz, 1H, H–6′), 11.56 (s, 1H, NH); $^{13}$C NMR (100 MHz, DMSO-$d_6$): δ 55.58 (4′–$OCH_3$), 55.62 (7–$OCH_3$), 55.68 (5–$OCH_3$), 56.01 (2′–$OCH_3$), 91.43 (C–8), 94.03 (C–6), 97.94 (C–3′), 103.96 (C–5′), 105.13 (C–4a), 119.42 (C–3), 124.20 (C–1′), 128.72 (C–6′), 142.09 (C–8a), 148.78 (C–4), 157.76 (C–2′), 158.88 (C–5), 160.50 (C–4′), 161.90 (C=O), 162.00 (C–7); HRMS (ESI) m/z calcd for $C_{19}H_{19}NO_5^+$ [M]$^+$: 341.1263, found [M+H]$^+$: 342.1332.

3.10. 4-(2,5-Dimethoxyphenyl)-5,7-dimethoxyquinolin-2(1*H*)-one (**28**)

Compound **28** (0.30 g, 0.88 mmole) was obtained by cyclization of compound **14** (0.88 g, 2.45 mmole). TLC monitored (ethyl acetate; Rf = 0.15). Yield 35.9% was recrystallized by ethanol/ethyl acetate as a light-yellow cotton crystal; Mp = 236–238 °C; IR (KBr) m (cm$^{-1}$): 1664 (C=O); $^1$H NMR (400 MHz, DMSO-$d_6$): δ 3.35 (s, 3H, 5–$OCH_3$), 3.57 (s, 3H, 2′–$OCH_3$), 3.71 (s, 3H, 5′–$OCH_3$), 3.79 (s, 3H, 7–$OCH_3$), 5.90 (s, 1H, H–3), 6.21 (d, *J* = 1.2 Hz, 1H, H–6), 6.50 (d, *J* = 1.6 Hz, 1H, H–8), 6.68 (d, *J* = 2.0 Hz, 1H, H–6′), 6.84–6.88 (m, 2H, H–3′,5′), 11.64 (s, 1H, NH); $^{13}$C NMR (100 MHz, DMSO-$d_6$): δ 55.74 (2C, 2′,5′–$OCH_3$), 55.94 (7–$OCH_3$), 55.05 (5–$OCH_3$), 91.48 (C–8), 94.10 (C–6), 104.79 (C–4a), 111.09 (C–3′), 112.97 (C–4′), 114.67 (C–6′), 119.19 (C–3), 132.23 (C–1′), 142.08 (C–8a), 148.50 (C–4), 150.76 (C–5′), 152.85 (C–2′), 158.72 (C–5), 161.92 (C=O), 162.04 (C–7); HRMS (ESI) m/z calcd for $C_{19}H_{19}NO_5^+$ [M]$^+$: 341.1263, found [M+H]$^+$: 342.1335.

3.11. 5,6,7-Trimethoxy-4-phenylquinolin-2(1*H*)-one (**29**)

Compound **29** (0.12 g, 0.39 mmole) was obtained by cyclization of compound **15** (0.36 g, 1.09 mmole). TLC monitored (ethyl acetate; Rf = 0.2). Yield 35.8% was recrystallized by ethyl acetate as a white needle crystal; Mp = 274–276 °C; IR (KBr) m (cm$^{-1}$): 1654 (C=O); $^1$H NMR (400 MHz, DMSO-$d_6$): δ 3.12 (s, 3H, 5–$OCH_3$), 3.64 (s, 3H, 6–$OCH_3$), 3.85 (s, 3H, 7–$OCH_3$), 5.97 (s, 1H, H–3), 6.78 (s, 1H, H–8), 7.28–7.30 (m, 2H, H–2′,6′), 7.35–7.40 (m, 3H, H–3′,4′,5′), 11.70 (s, 1H, NH); $^{13}$C NMR (100 MHz, DMSO-$d_6$): δ 56.22 (7–$OCH_3$), 60.83 (5–$OCH_3$), 60.95 (6–$OCH_3$), 94.40 (C–8), 107.34 (C–4a), 120.66 (C–3), 127.41 (C–4′), 127.58 (2C, C–3′,5′), 127.69 (2C, C–2′,6′), 137.54 (C–8a), 137.79 (C–6), 141.31 (C–1′), 150.82 (C–4), 151.07 (C–5), 156.29 (C–7), 161.26 (C=O); HRMS (ESI) m/z calcd for $C_{18}H_{17}NO_4^+$ [M]$^+$: 311.1158, found [M+H]$^+$: 312.1226.

3.12. 5,6,7-Trimethoxy-4-(4-methoxyphenyl)quinolin-2(1*H*)-one (**30**)

Compound **30** (0.15 g, 0.44 mmole) was obtained by cyclization of compound **16** (1.59 g, 4.43 mmole). TLC monitored (ethyl acetate; Rf = 0.07). Yield 9.9% was recrystallized by ethanol/ethyl acetate as a light-brown solid; Mp = 269–271 °C; IR (KBr) m (cm$^{-1}$): 1654 (C=O); $^1$H NMR (400 MHz, DMSO-$d_6$): δ 3.14 (s, 3H, 5–$OCH_3$), 3.65 (s, 3H, 6–$OCH_3$), 3.79 (s, 3H, 4′–$OCH_3$), 3.84 (s, 3H, 7–$OCH_3$), 5.97 (s, 1H, H–3), 6.78 (s, 1H, H–8), 6.99 (d, *J* = 8.4 Hz, 2H, H–3′,5′), 7.23 (d, *J* = 8.4 Hz, 2H, H–2′,6′), 11.67 (s, 1H, NH); $^{13}$C NMR (100 MHz, DMSO-$d_6$): δ 55.48 (7–$OCH_3$), 56.18 (4′–$OCH_3$), 60.98 (2C, 5,6–$OCH_3$), 94.44 (C–8), 107.43 (C–4a), 112.96 (2C, C–3′,5′), 120.83 (C–3), 129.16 (2C, C–2′,6′), 133.40 (C–1′), 137.60 (C–8a), 137.85 (C–6), 150.88 (C–4), 150.98 (C–5), 156.16 (C–7), 158.83 (C–4′), 161.32 (C=O); HRMS (ESI) m/z calcd for $C_{19}H_{19}NO_5^+$ [M]$^+$: 341.1263, found [M+H]$^+$: 342.1332.

3.13. 4-(2,4-Dimethoxyphenyl)-5,6,7-trimethoxyquinolin-2(1*H*)-one (**31**)

Compound **31** (0.37 g, 1.00 mmole) was obtained by cyclization of compound **17** (0.62 g, 1.59 mmole). TLC monitored (ethyl acetate; Rf = 0.3). Yield 62.9% was recrystallized by ethyl acetate as a white square crystal; Mp = 216–218 °C; IR (KBr) m (cm$^{-1}$): 1651 (C=O); $^1$H NMR (400 MHz, DMSO-$d_6$): δ 3.15 (s, 3H, 5–$OCH_3$), 3.63 (s, 6H, 2′,6–$OCH_3$), 3.80 (s, 3H, 4′–$OCH_3$), 3.83 (s, 3H, 7–$OCH_3$), 5.94 (s, 1H, H–3), 6.53 (d, *J* = 8.0 Hz, 1H, H–5′), 6.56 (s, 1H, H–3′), 6.74 (s, 1H, H–8), 7.07 (d, *J* = 8.0 Hz, 1H, H–6′), 11.63 (s, 1H, NH); $^{13}$C NMR (100 MHz, DMSO-$d_6$): δ 55.57 (2′–$OCH_3$), 55.62 (4′–$OCH_3$), 56.12 (7–$OCH_3$), 60.91 (6–$OCH_3$), 61.11 (5–$OCH_3$), 94.21 (C–8), 98.07 (C–3′), 104.04 (C–5′), 108.40 (C–4a), 120.74 (C–3), 123.34 (C–1′), 128.80 (C–6′), 136.99 (C–8a), 137.58 (C–6), 148.36 (C–4), 151.24 (C–5), 155.82 (C–7), 157.77 (C–2′), 160.57 (C–4′), 161.66 (C=O); HRMS (ESI) m/z calcd for $C_{20}H_{21}NO_6^+$ [M]$^+$: 371.1369, found [M+H]$^+$: 372.1435.

3.14. 4-(2,5-Dimethoxyphenyl)-5,6,7-trimethoxyquinolin-2(1*H*)-one (**32**)

Compound **32** (0.16 g, 0.43 mmole) was obtained by cyclization of compound **18** (0.40 g, 1.03 mmole). TLC monitored (ethyl acetate; Rf = 0.25). Yield 41.7% was recrystallized by ethyl acetate as a light-yellow cotton crystal; Mp = 243–245 °C; IR (KBr) m (cm$^{-1}$): 1664 (C=O); $^1$H NMR (400 MHz, DMSO-$d_6$): δ 3.19 (s, 3H, 5–$OCH_3$), 3.60 (s, 3H, 2′–$OCH_3$), 3.64 (s, 3H, 6–$OCH_3$), 3.72 (s, 3H, 5′–$OCH_3$), 3.84 (s, 3H, 7–$OCH_3$), 5.96 (s, 1H, H–3), 6.74 (s, 1H, H–8), 6.76 (d, *J* = 2.0 Hz, H–6′), 6.88 (dd, *J* = 8.8, 2.4 Hz, 2H, H–4′), 6.93 (d, *J* = 8.8 Hz, H–3′), 11.63 (s, 1H, NH); $^{13}$C NMR (100 MHz, DMSO-$d_6$): δ 55.82 (2′–$OCH_3$), 55.96 (5′–$OCH_3$), 56.17 (7–$OCH_3$), 60.88 (6–$OCH_3$), 60.95 (5–$OCH_3$), 94.17 (C–8), 108.09 (C–4a), 111.25 (C–3′), 113.02 (C–4′), 114.79 (C–6′), 120.47 (C–3), 131.52 (C–1′), 136.99 (C–8a), 137.50 (C–6), 148.09 (C–4), 150.59 (C–5′), 150.99 (C–5), 152.85 (C–2′), 155.98 (C–7), 161.56 (C=O); HRMS (ESI) m/z calcd for $C_{20}H_{21}NO_6^+$ [M]$^+$: 371.1369, found [M+H]$^+$: 372.1436.

*4. Meta-Pred Web Server prediction results of site of metabolism for compound* **22**

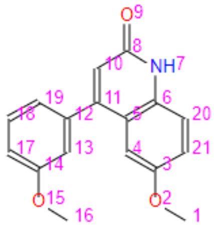


**Table S1.** Meta-Pred Web Server prediction results of site of metabolism for enzyme **CYP3A4**. Date of prediction is 2022-07-29

| Atom Number | Rank | DeltaP |
|---|---|---|
| 1 | 1 | 0,696 |
| 16 | 1 | 0,696 |
| 19 | 2 | 0,350 |
| 18 | 3 | 0,337 |
| 10 | 4 | 0,301 |
| 17 | 5 | 0,186 |
| 13 | 6 | 0,183 |
| 21 | 7 | 0,178 |
| 20 | 8 | 0,174 |
| 4 | 9 | 0,080 |
| 7 | 10 | -0,821 |
| 2 | 11 | -0,812 |
| 15 | 11 | -0,812 |
| 5 | 12 | -0,808 |
| 6 | 13 | -0,627 |
| 11 | 14 | -0,595 |
| 8 | 15 | -0,541 |
| 9 | 16 | -0,524 |
| 12 | 17 | -0,450 |
| 14 | 18 | -0,387 |
| 3 | 19 | -0,292 |

**Table S2.** Meta-Pred Web Server prediction results of site of metabolism for enzyme **CYP2D6**. Date of prediction is 2022-07-29

| Atom Number | Rank | DeltaP |
|---|---|---|
| 1 | 1 | 0,788 |
| 16 | 1 | 0,788 |
| 18 | 2 | 0,138 |
| 19 | 3 | 0,035 |
| 7 | 4 | -0,988 |
| 2 | 5 | -0,919 |
| 15 | 5 | -0,919 |
| 6 | 6 | -0,880 |
| 5 | 7 | -0,876 |
| 11 | 8 | -0,830 |
| 8 | 9 | -0,787 |
| 12 | 10 | -0,786 |
| 9 | 11 | -0,765 |
| 14 | 12 | -0,560 |
| 3 | 13 | -0,460 |
| 4 | 14 | -0,295 |
| 10 | 15 | -0,217 |
| 13 | 16 | -0,203 |
| 21 | 17 | -0,082 |
| 17 | 18 | -0,069 |
| 20 | 19 | -0,021 |

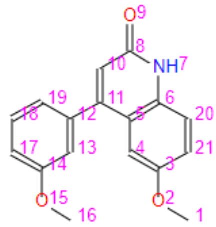


**Table S3.** Meta-Pred Web Server prediction results of site of metabolism for enzyme **CYP2C9**
Date of prediction is 2022-07-29

| Atom Number | Rank | DeltaP |
|---|---|---|
| 1 | 1 | 0,742 |
| 16 | 1 | 0,742 |
| 18 | 2 | 0,082 |
| 17 | 3 | 0,023 |
| 7 | 4 | -0,961 |
| 6 | 5 | -0,941 |
| 5 | 6 | -0,896 |
| 8 | 7 | -0,886 |
| 2 | 8 | -0,875 |
| 15 | 8 | -0,875 |
| 11 | 9 | -0,849 |
| 12 | 10 | -0,779 |
| 14 | 11 | -0,654 |
| 9 | 12 | -0,577 |
| 3 | 13 | -0,455 |
| 4 | 14 | -0,285 |
| 13 | 15 | -0,260 |
| 20 | 16 | -0,158 |
| 10 | 17 | -0,126 |
| 19 | 18 | -0,048 |
| 21 | 19 | -0,028 |

**Table S4.** Meta-Pred Web Server prediction results of site of metabolism for enzyme **CYP2C19**
Date of prediction is 2022-07-29

| Atom Number | Rank | DeltaP |
|---|---|---|
| 1 | 1 | 0,731 |
| 16 | 1 | 0,731 |
| 17 | 2 | 0,208 |
| 21 | 3 | 0,187 |
| 18 | 4 | 0,181 |
| 7 | 5 | -0,973 |
| 6 | 6 | -0,895 |
| 5 | 7 | -0,855 |
| 2 | 8 | -0,841 |
| 15 | 8 | -0,841 |
| 12 | 9 | -0,780 |
| 11 | 10 | -0,763 |
| 9 | 11 | -0,639 |
| 8 | 12 | -0,628 |
| 14 | 13 | -0,532 |
| 3 | 14 | -0,423 |
| 20 | 15 | -0,252 |
| 4 | 16 | -0,242 |
| 10 | 17 | -0,175 |
| 13 | 18 | -0,123 |
| 19 | 19 | -0,105 |

**Table S5.** Meta-Pred Web Server prediction results of site of metabolism for enzyme **CYP1A2** Date of prediction is 2022-07-29

| Atom Number | Rank | DeltaP |
|---|---|---|
| 1 | 1 | 0,675 |
| 16 | 1 | 0,675 |
| 19 | 2 | 0,250 |
| 18 | 3 | 0,215 |
| 10 | 4 | 0,202 |
| 17 | 5 | 0,149 |
| 21 | 6 | 0,103 |
| 13 | 7 | 0,100 |
| 20 | 7 | 0,100 |
| 4 | 8 | 0,032 |
| 7 | 9 | -0,797 |
| 2 | 10 | -0,753 |
| 15 | 10 | -0,753 |
| 5 | 11 | -0,676 |
| 6 | 12 | -0,597 |
| 11 | 13 | -0,530 |
| 9 | 14 | -0,512 |
| 12 | 15 | -0,441 |
| 14 | 16 | -0,396 |
| 8 | 17 | -0,380 |
| 3 | 18 | -0,342 |

## 5. *Spectral copies of ¹HNMR, ¹³C NMR, IR and Mass of the 4-PQ compounds* ***19-32***

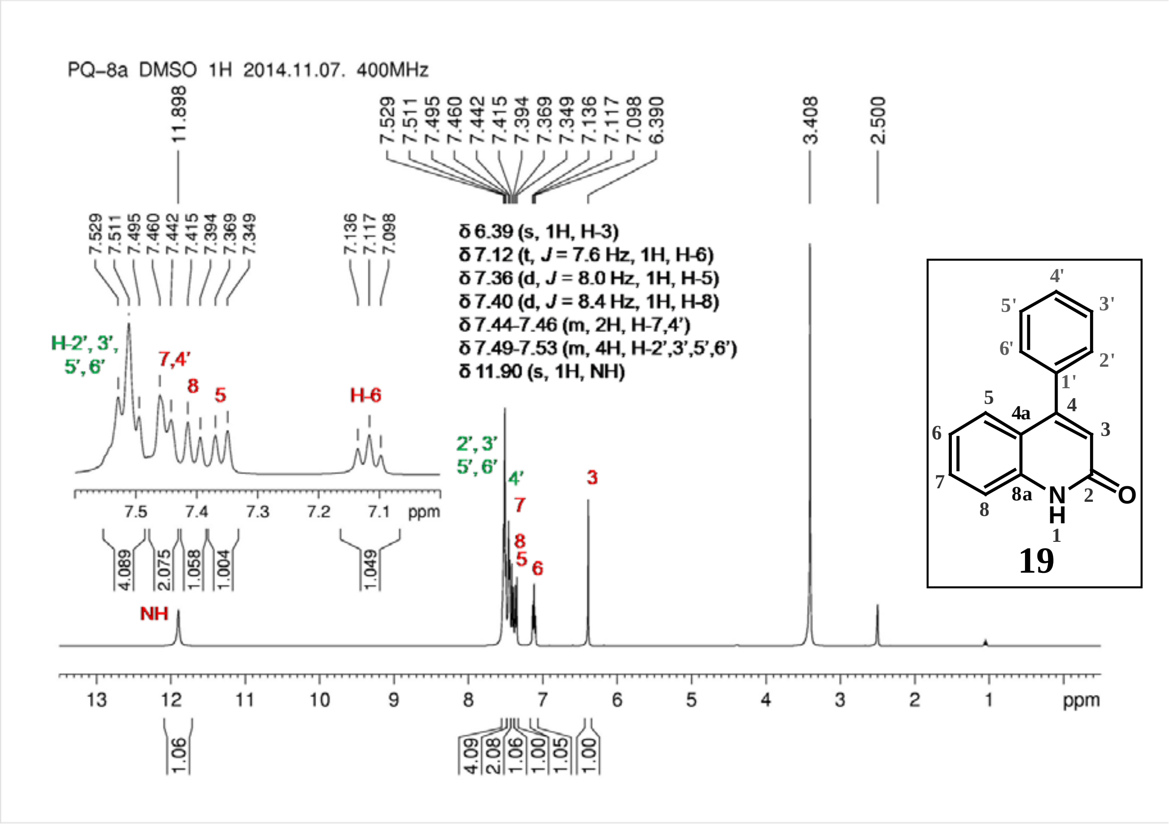


**Figure S1.** ¹H-NMR Spectra of compound **4-Phenylquinolin-2(1*H*)-one (19)** was recorded in DMSO-$d_6$ (400MHz)

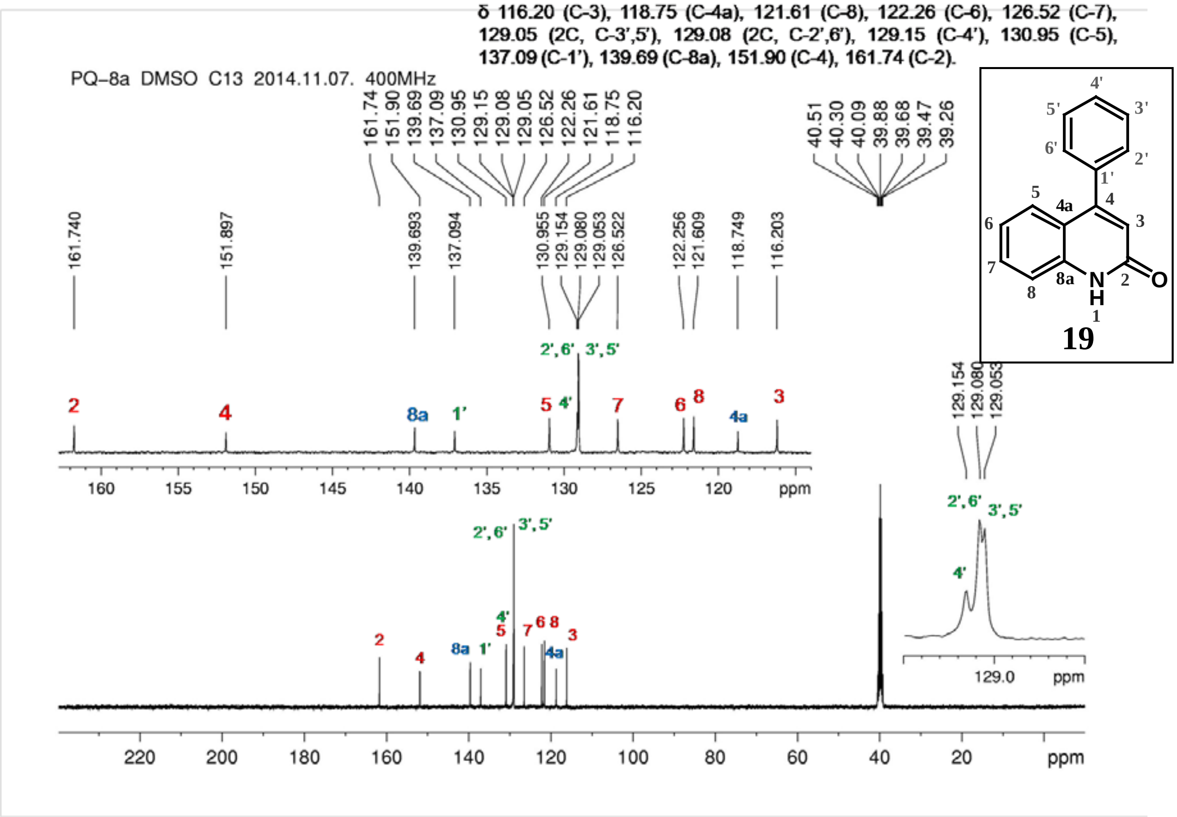


**Figure S2.** ¹³C-NMR Spectra of compound **4-Phenylquinolin-2(1*H*)-one (19)** was recorded in DMSO-$d_6$ (100MHz)

700, 750, 783, 844, 877, 906, 947, 983, 1033, 1074, 1149, 1267, 1384, 1431, 1492, 1548, 1597, 1616, 1664 (C=O), 2590-3167 (C-H), 3315 (N-H).

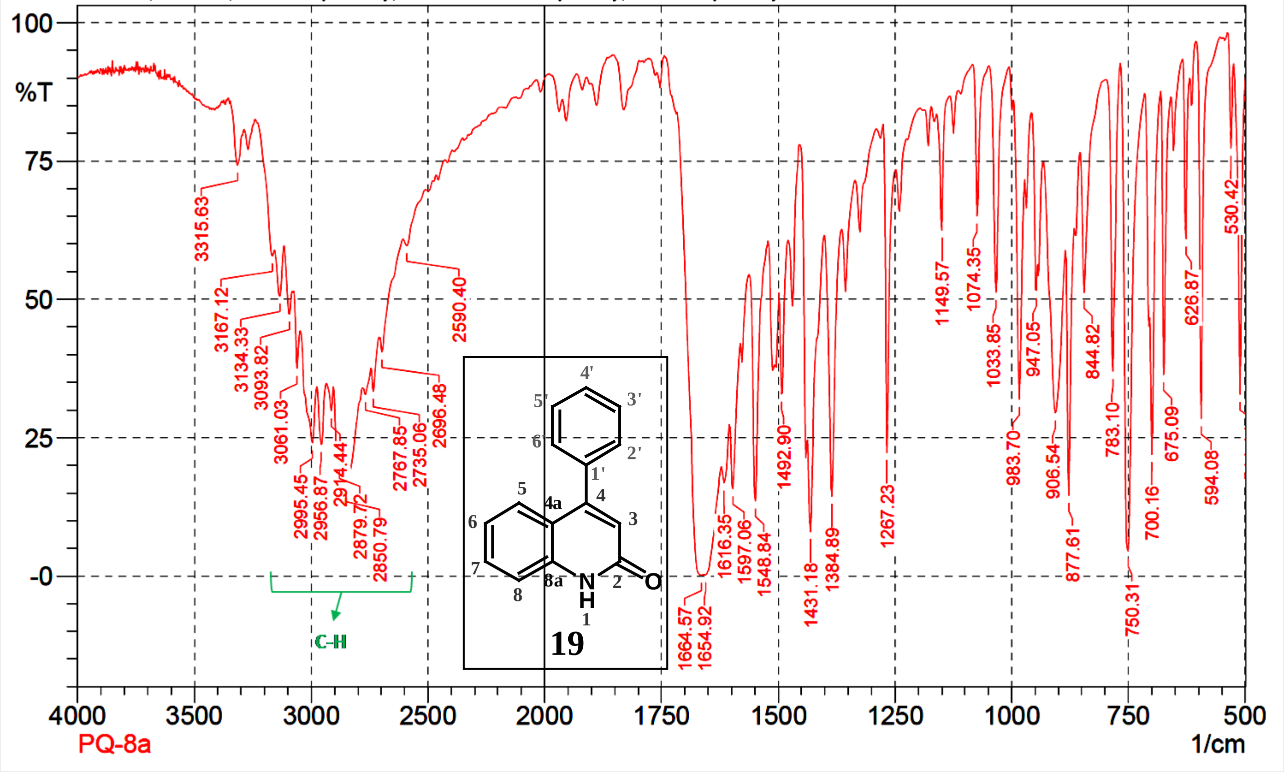


**Figure S3.** IR Spectra of compound **4-Phenylquinolin-2(1*H*)-one (19)**

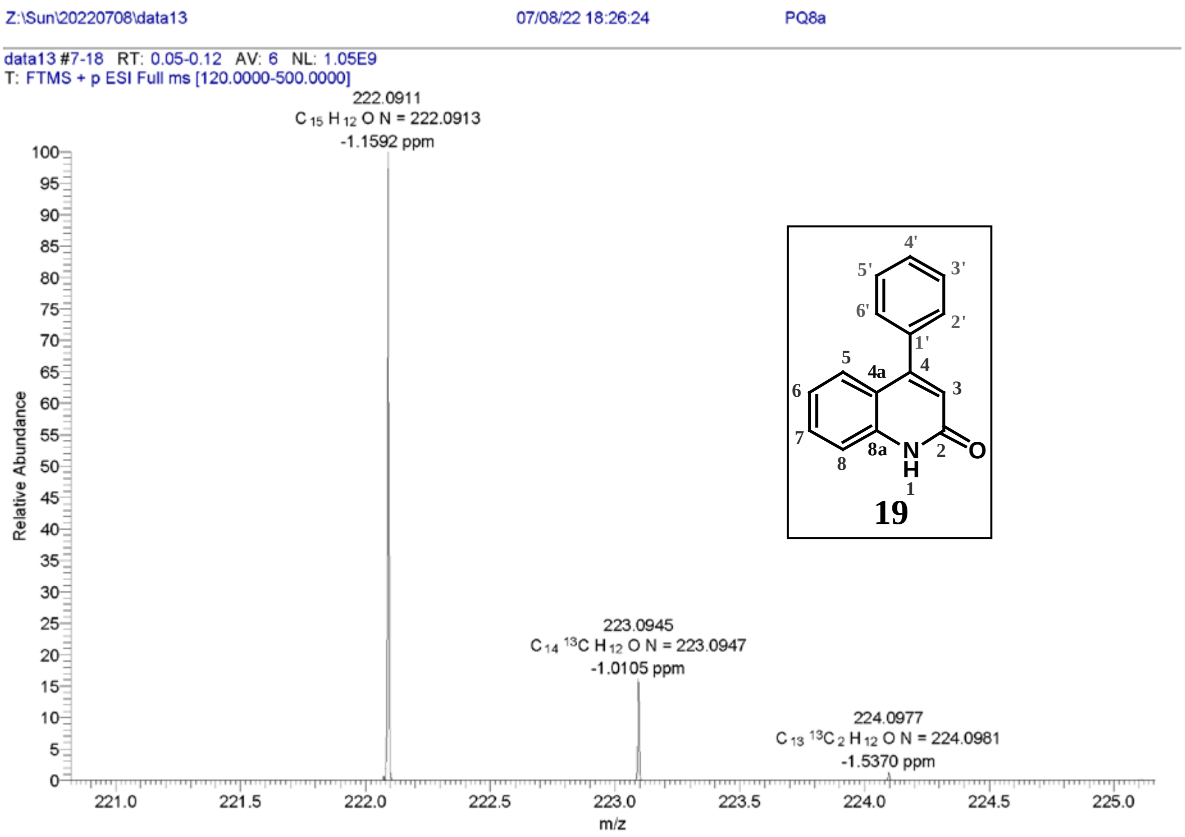


**Figure S4.** Mass Spectra of compound **4-Phenylquinolin-2(1*H*)-one (19)**

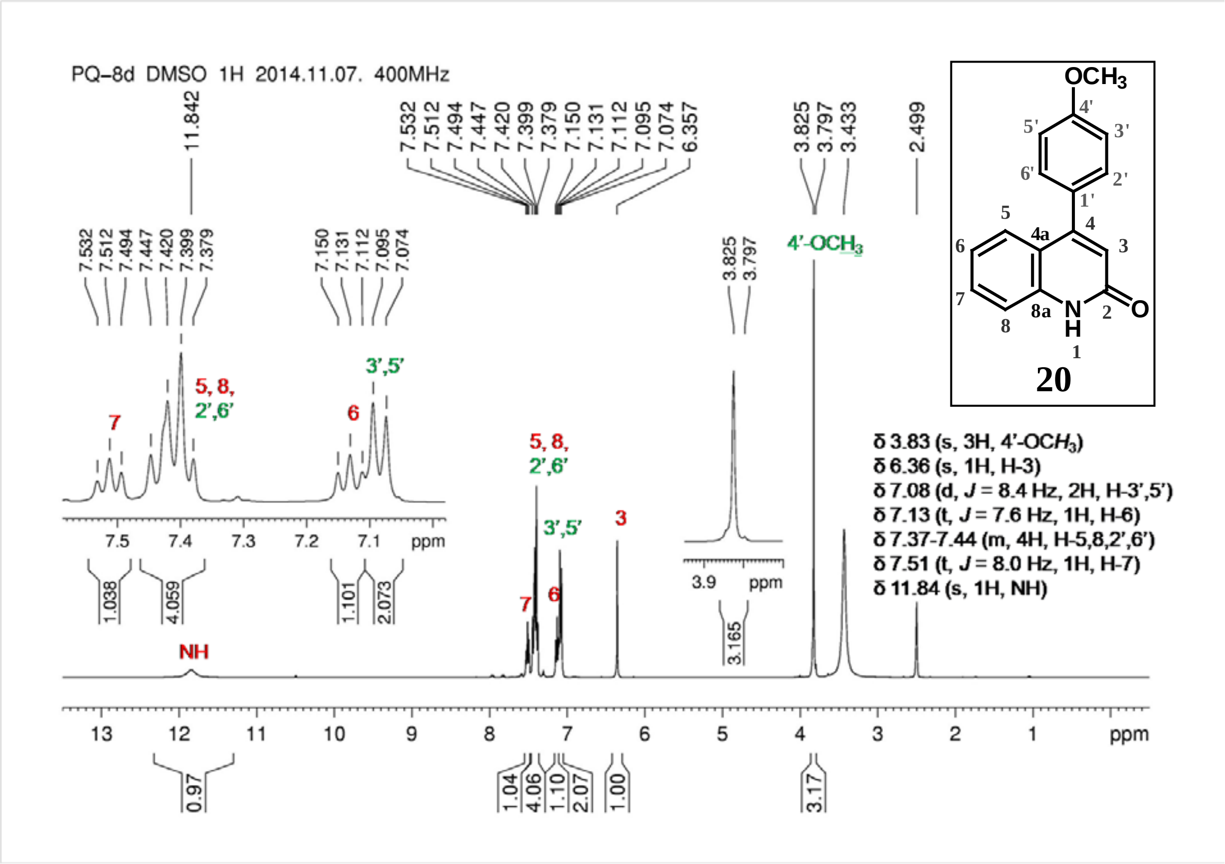


**Figure S5.** $^1$H-NMR Spectra of compound **4-(4-Methoxyphenyl)quinolin-2(1*H*)-one (20)** was recorded in DMSO-d$_6$ (400MHz)

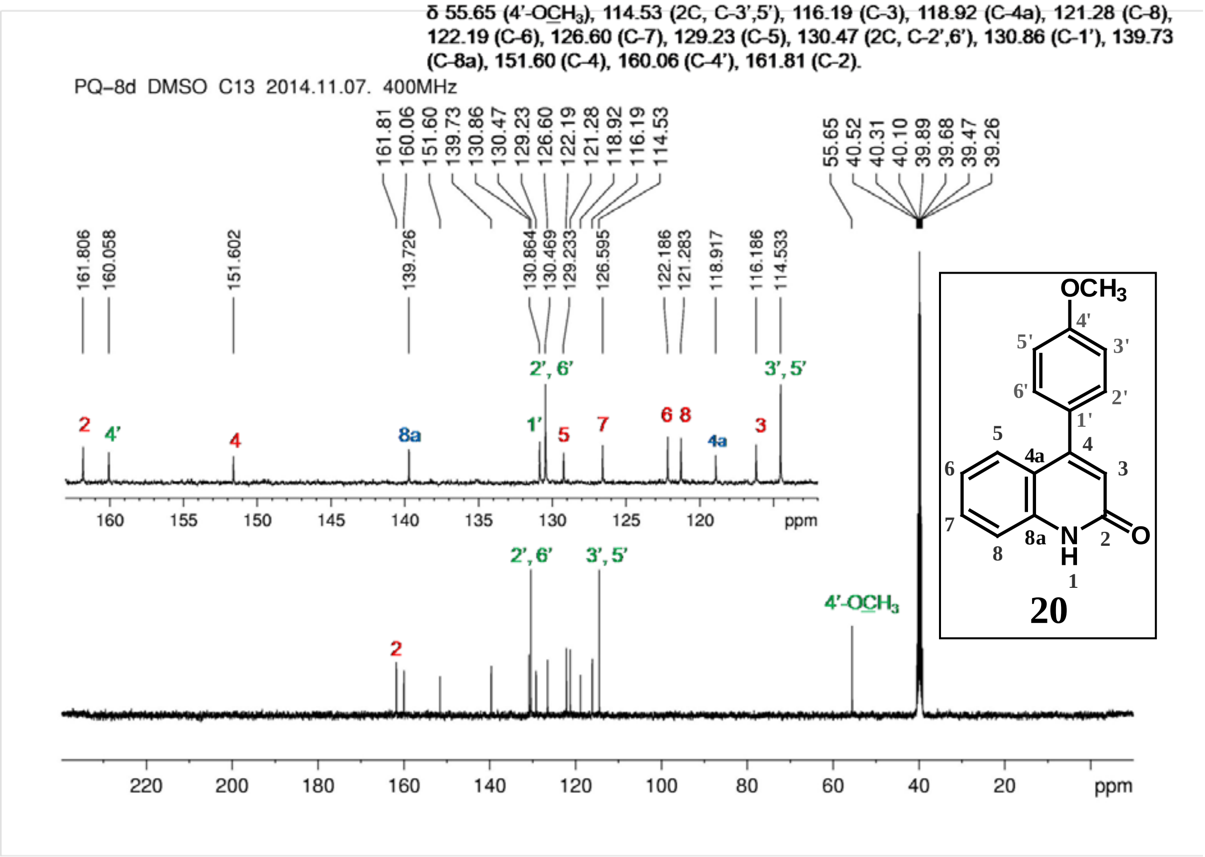


**Figure S6.** $^{13}$C-NMR Spectra of compound **4-(4-Methoxyphenyl)quinolin-2(1*H*)-one (20)** was recorded in DMSO-*d*$_6$ (100MHz)

607, 655, 750, 825, 879, 985, 1028, 1174, 1249, 1296, 1386, 1433, 1510, 1548, 1610, 1672 (C=O), 2698-3010 (C-H), 3435 (N-H).

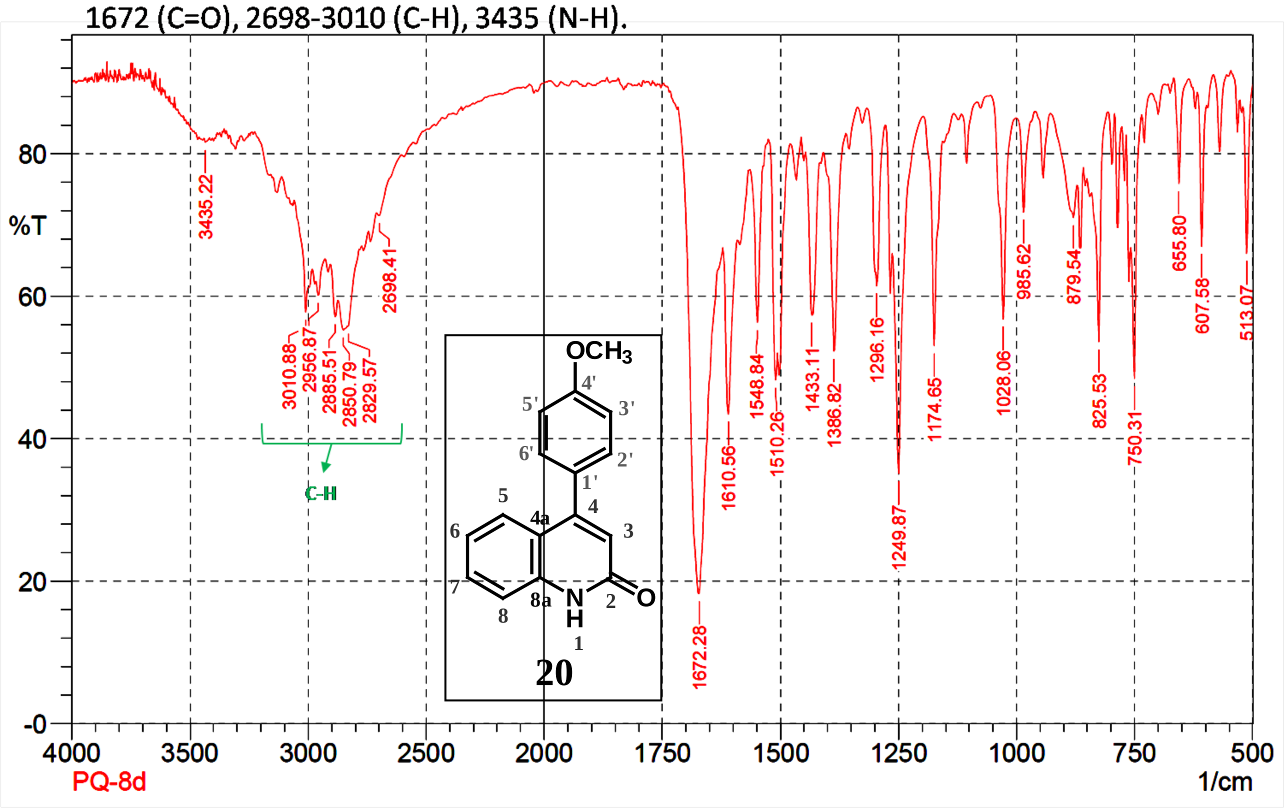


**Figure S7.** IR Spectra of compound **4-(4-Methoxyphenyl)quinolin-2(1*H*)-one (20)**

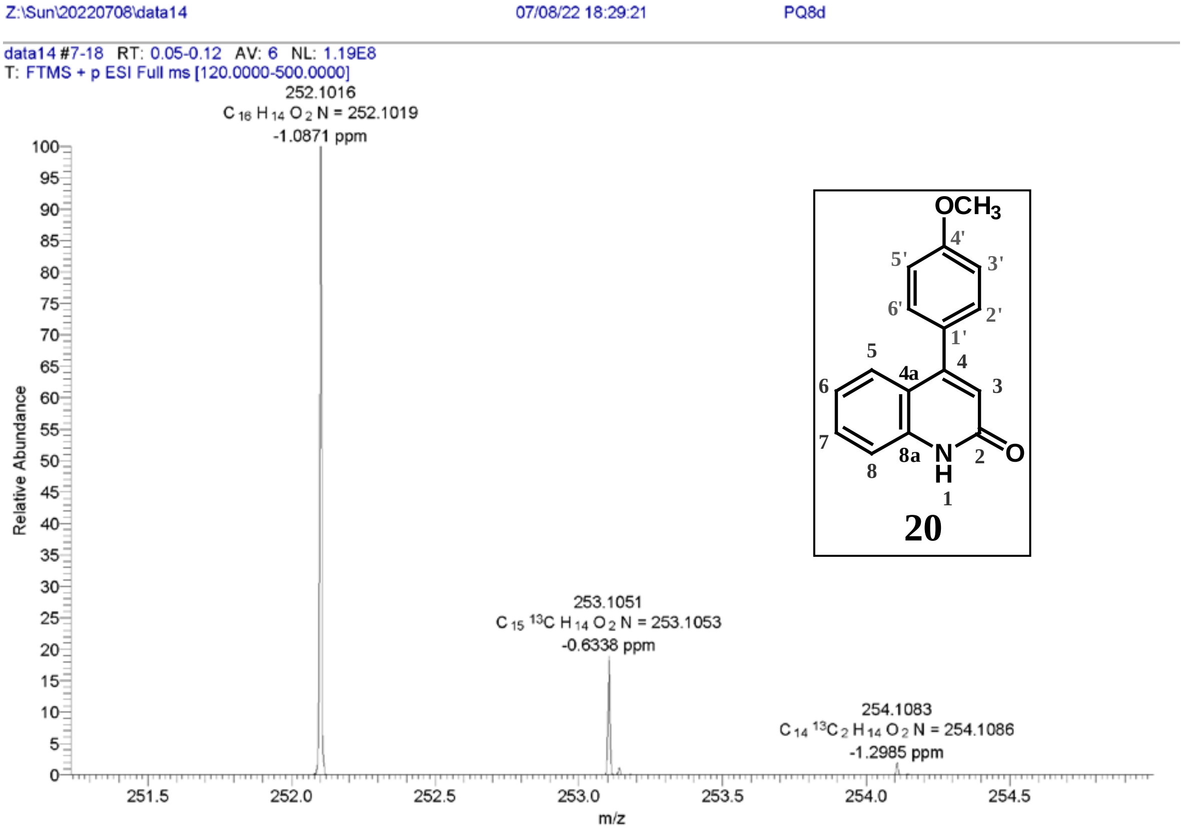


**Figure S8.** Mass Spectra of compound **4-(4-Methoxyphenyl)quinolin-2(1*H*)-one (20)**

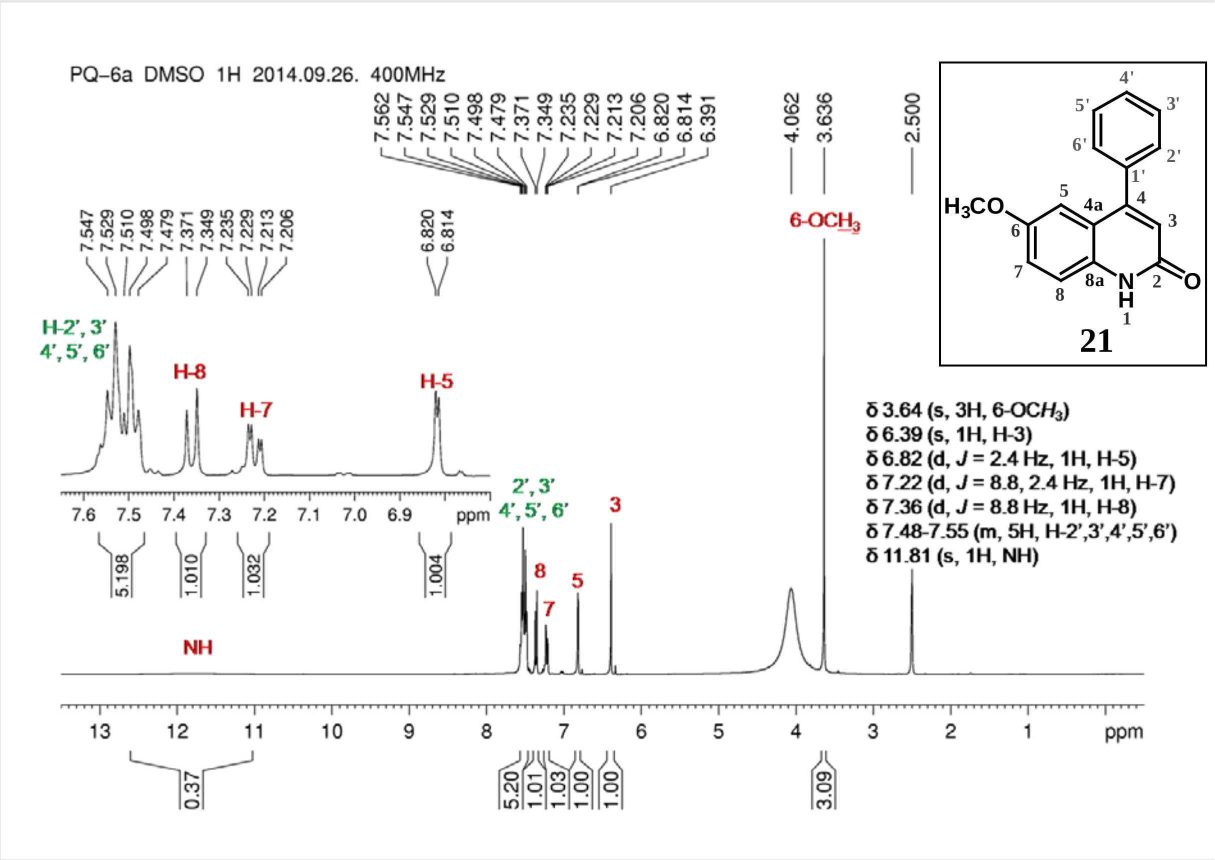


**Figure S9.** $^{1}$H-NMR Spectra of compound **6-Methoxy-4-phenylquinolin-2(1*H*)-one (21)** was recorded in DMSO-d6 (400MHz)

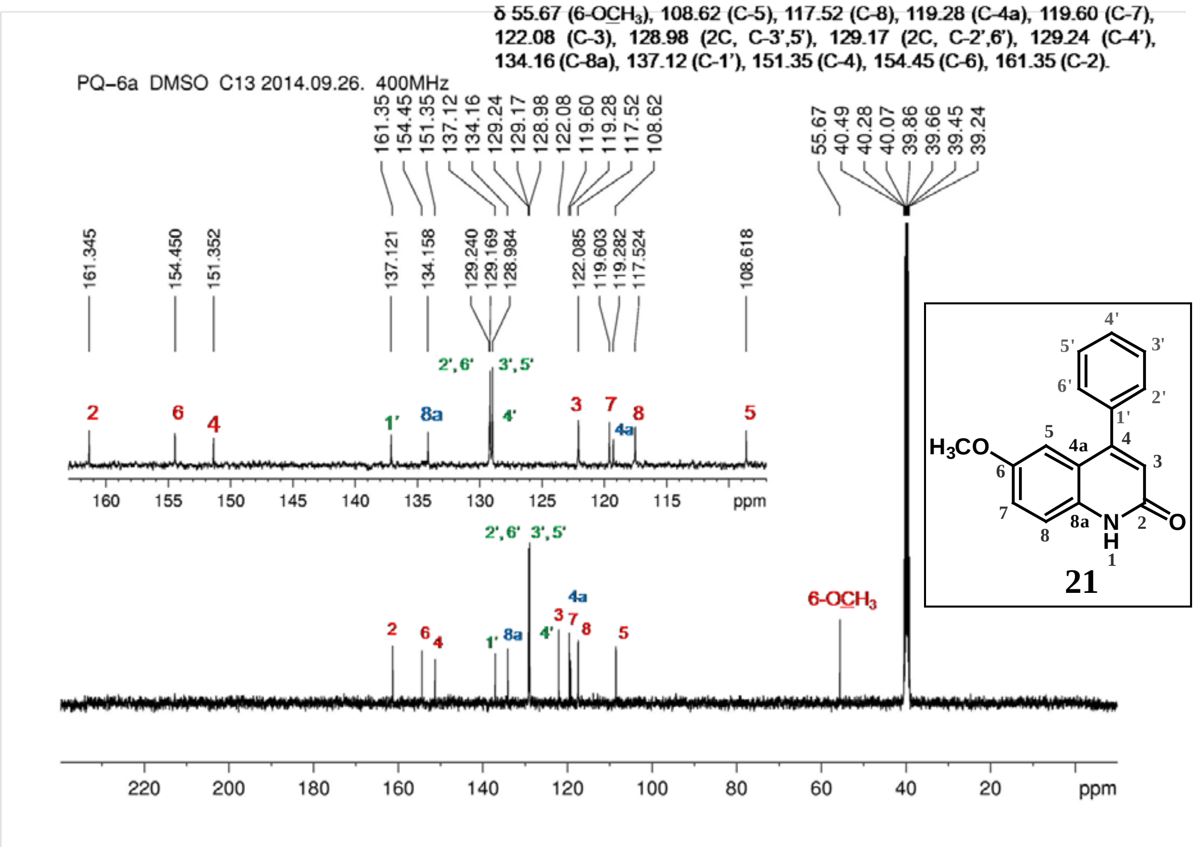


**Figure S10.** $^{13}$C-NMR Spectra of compound **6-Methoxy-4-phenylquinolin-2(1*H*)-one (21)** was recorded in DMSO-*d*6 (100MHz)

767, 815, 871, 1002, 1045, 1122, 1153, 1176, 1220, 1278, 1425, 1444, 1504, 1624, 1654 (C=O), 2644-3140 (C-H), 3294 (N-H).

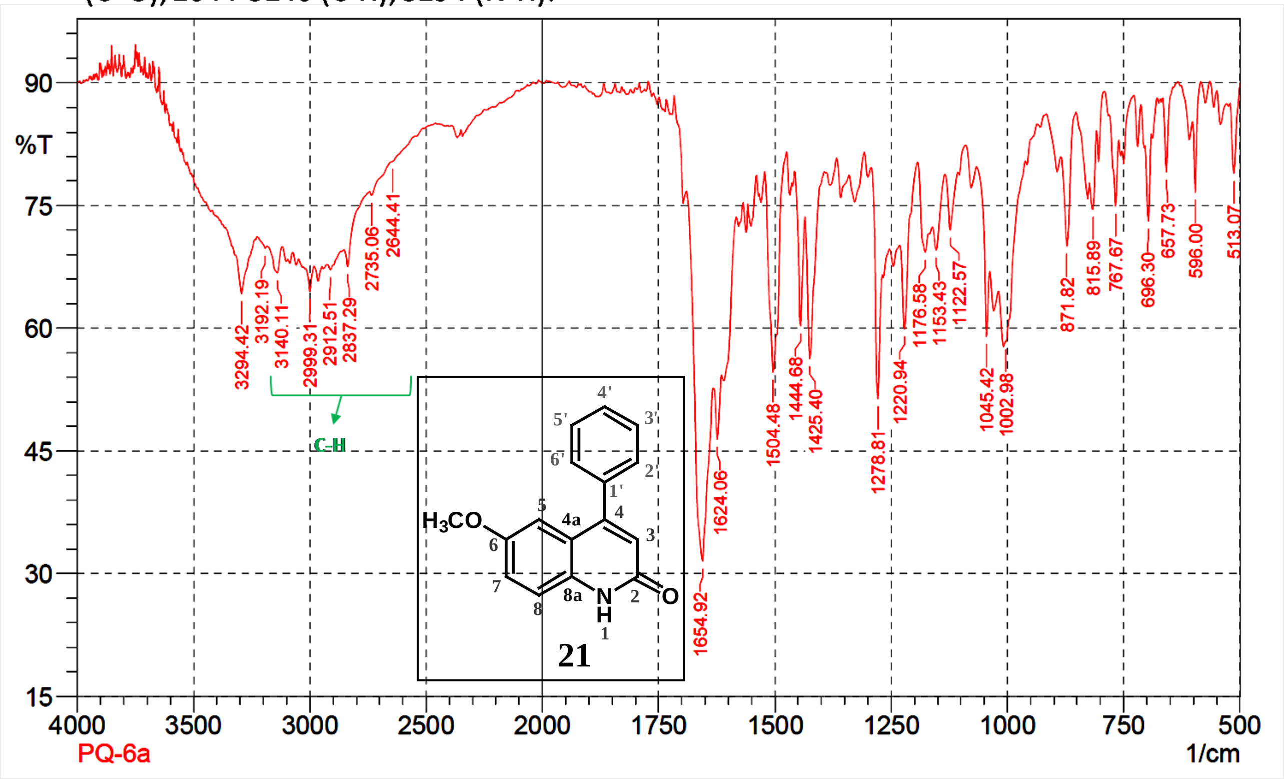


**Figure S11.** IR Spectra of compound **6-Methoxy-4-phenylquinolin-2(1*H*)-one (21)**

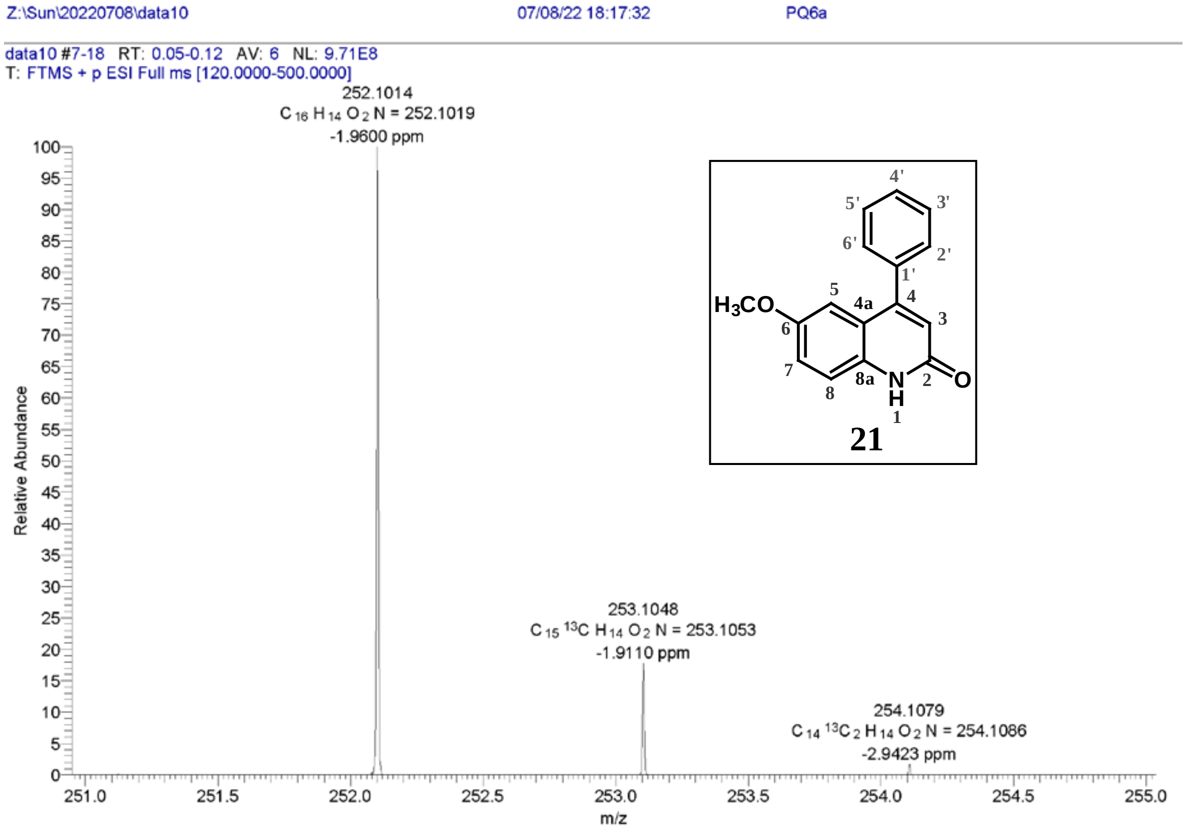


**Figure S12.** Mass Spectra of compound **6-Methoxy-4-phenylquinolin-2(1*H*)-one (21)**

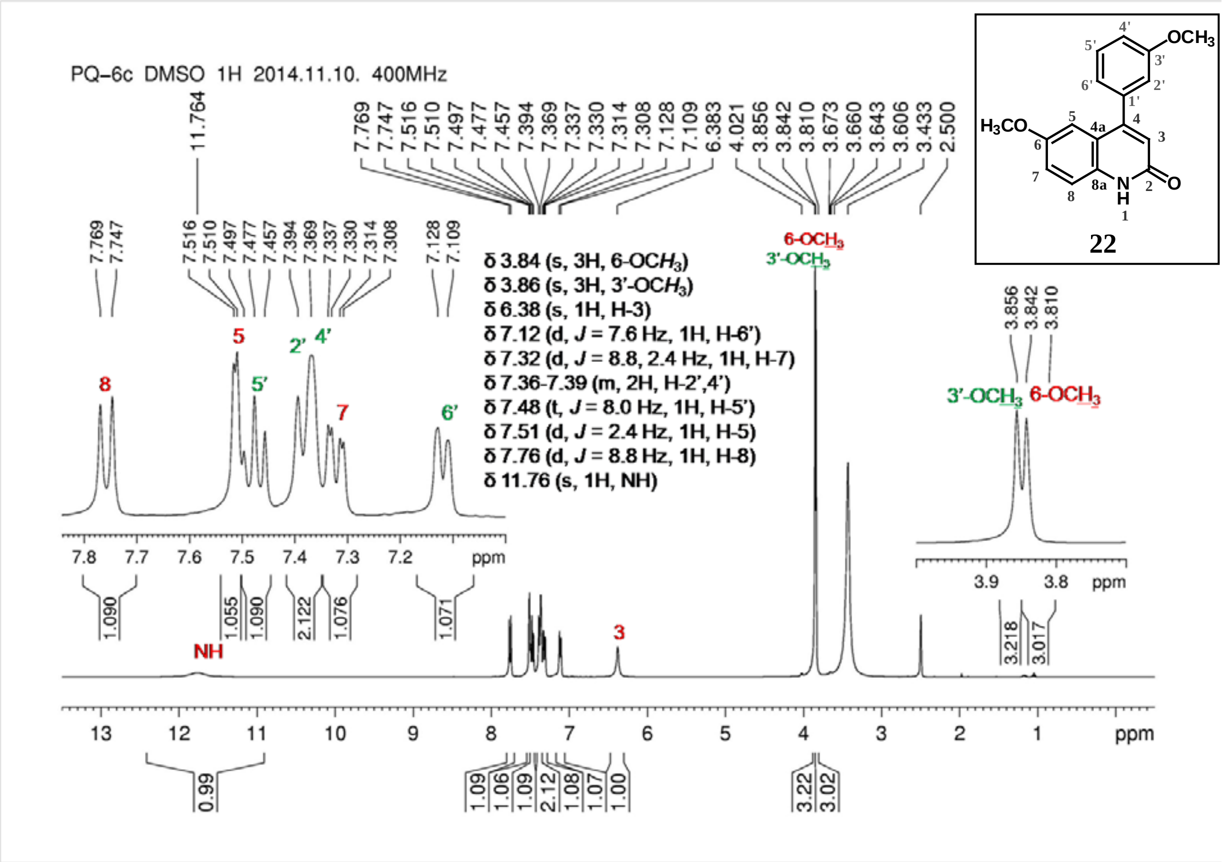


**Figure S13.** $^{1}$H-NMR Spectra of compound **6-Methoxy-4-(3-methoxyphenyl)quinolin-2(1*H*)-one (22)** was recorded in DMSO-d6 (400MHz)

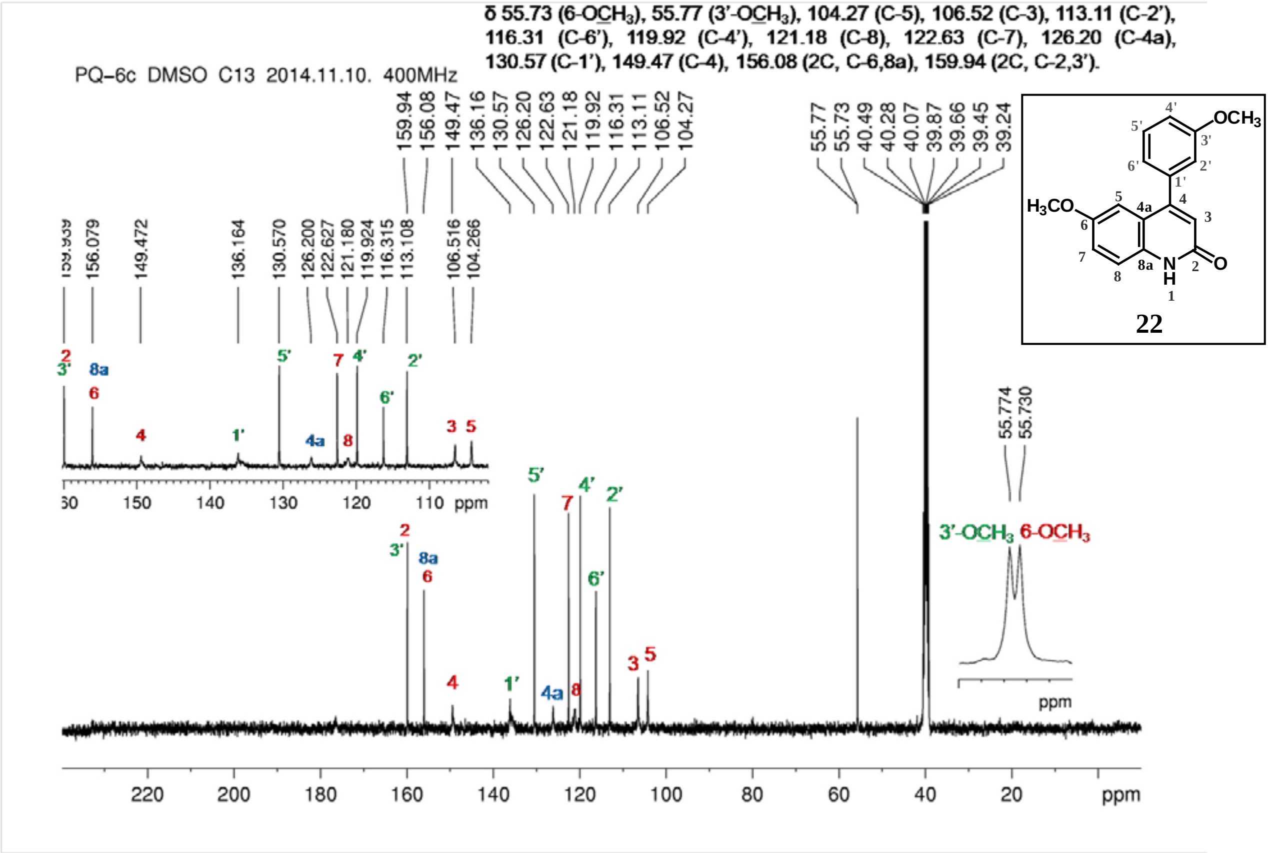


**Figure S14.** $^{13}$C-NMR Spectra of compound **6-Methoxy-4-(3-methoxyphenyl)quinolin-2(1*H*)-one (22)** was recorded in DMSO-*d6* (100MHz)

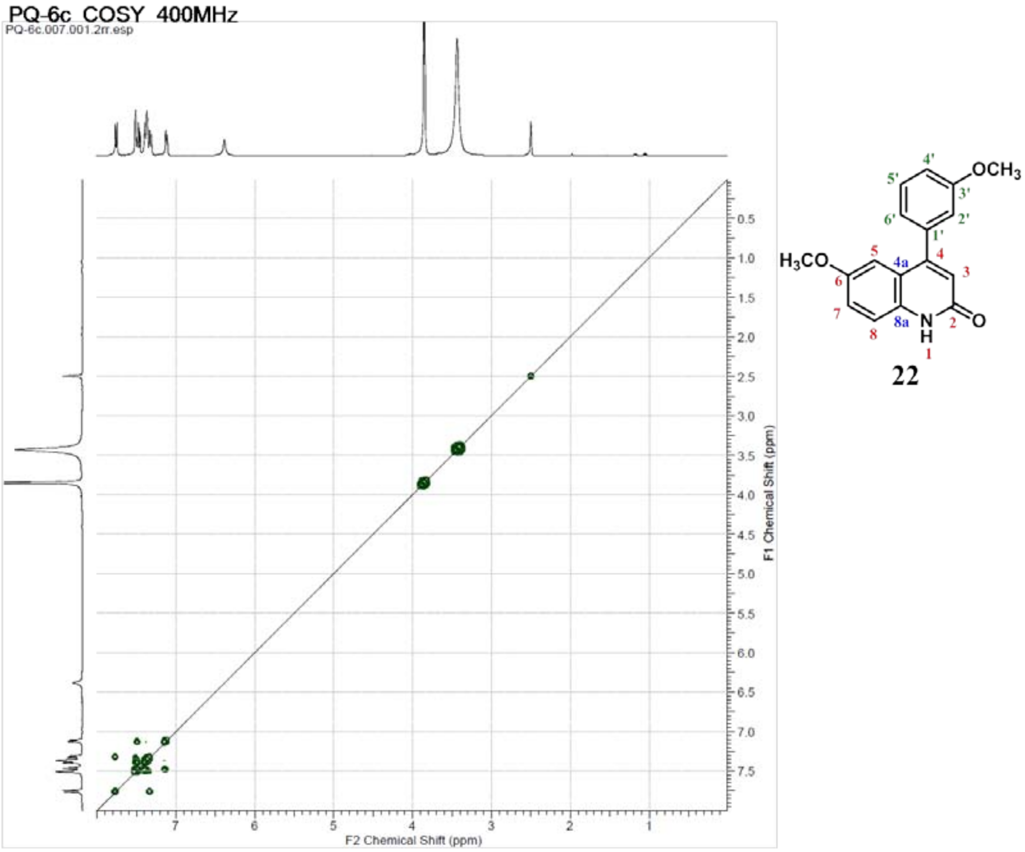


**Figure S15.** $^1$H-$^1$H COSY Spectra of compound **6-Methoxy-4-(3-methoxyphenyl)quinolin-2(1*H*)-one (22)** was recorded in DMSO-*d*$_6$ (400MHz)

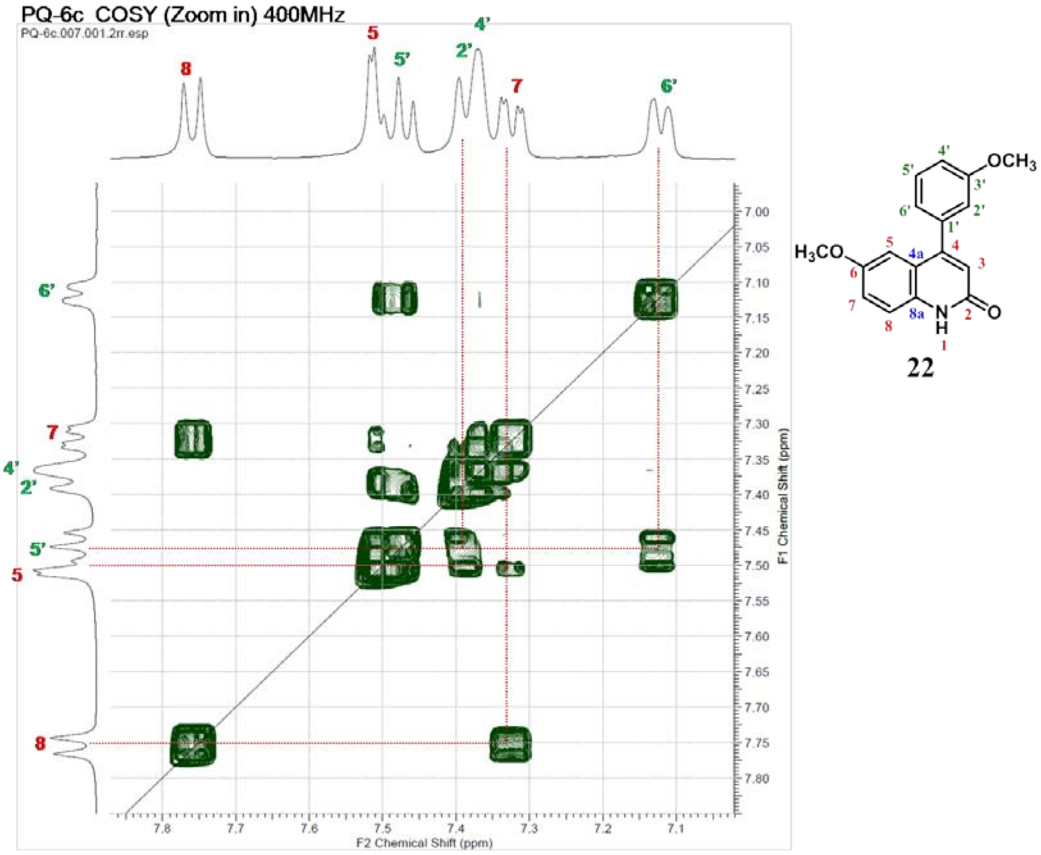


**Figure S16.** $^1$H-$^1$H COSY Spectra of compound **6-Methoxy-4-(3-methoxyphenyl)quinolin-2(1*H*)-one (22)** was recorded in DMSO-*d*$_6$ (400MHz Zoom in)

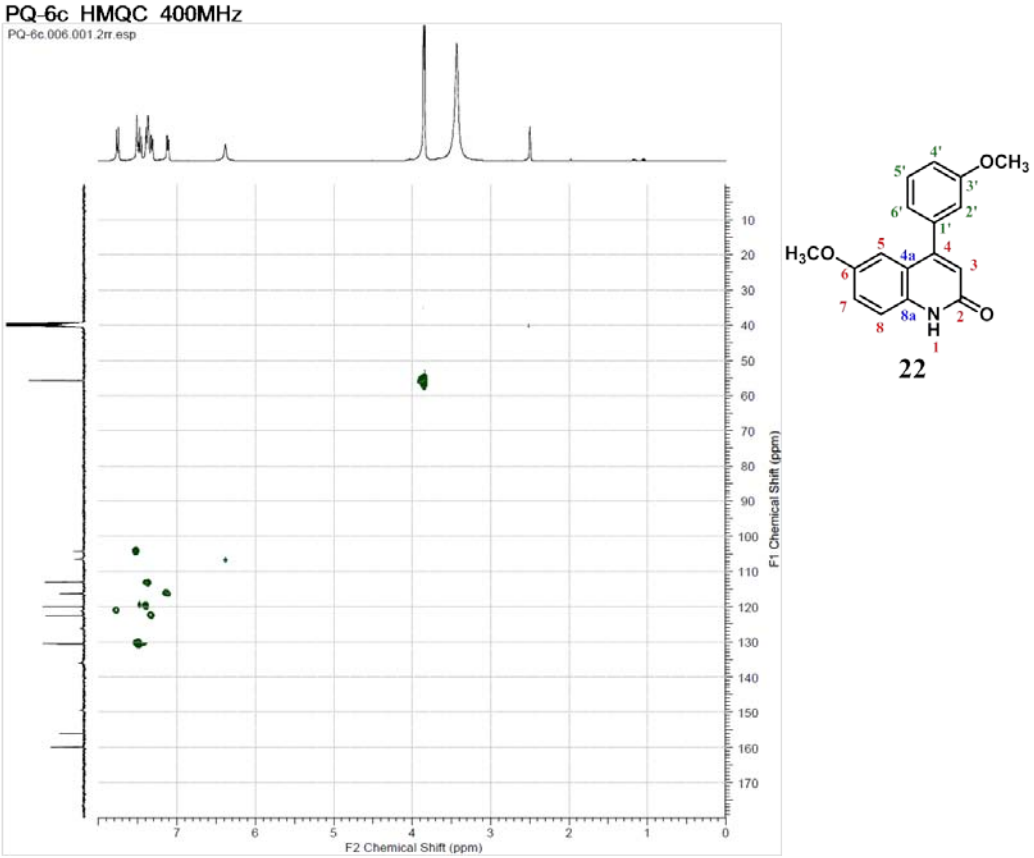


**Figure S17.** HMQC Spectra of compound **6-Methoxy-4-(3-methoxyphenyl)quinolin-2(1*H*)-one (22)** was recorded in DMSO-$d_6$ (400MHz)

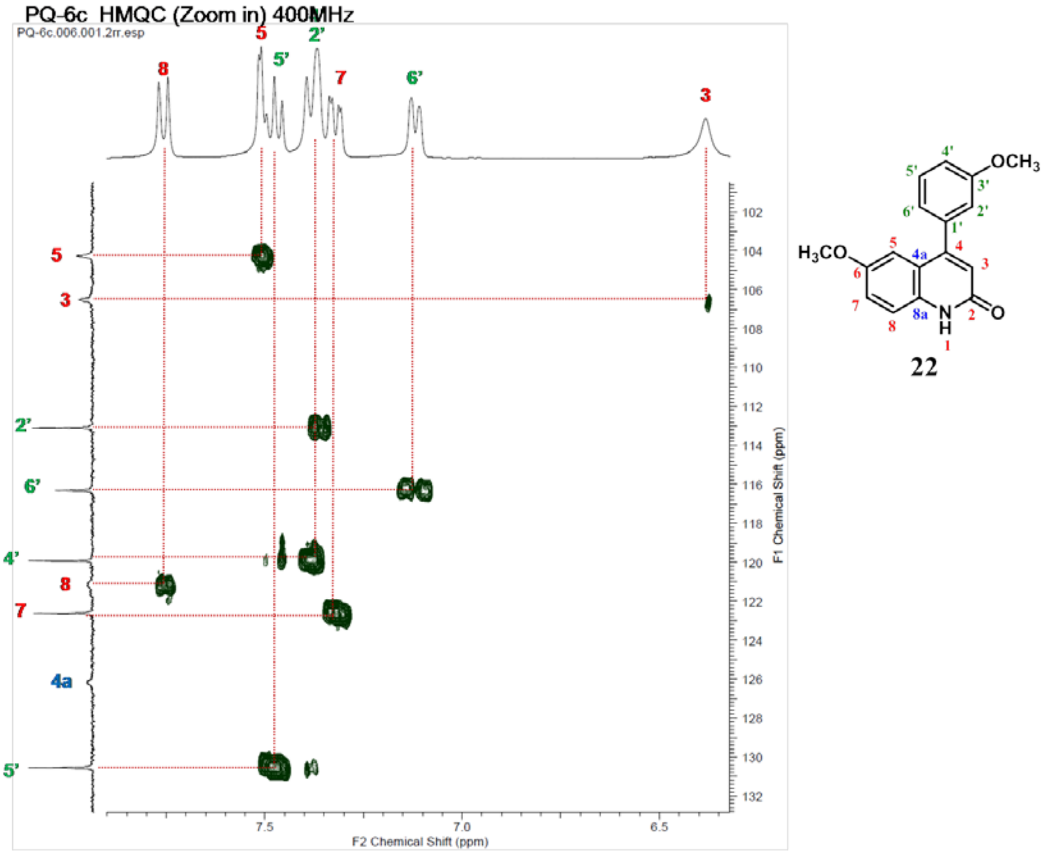


**Figure S18.** HMQC Spectra of compound **6-Methoxy-4-(3-methoxyphenyl)quinolin-2(1*H*)-one (22)** was recorded in DMSO-$d_6$ (400MHz Zoom in)

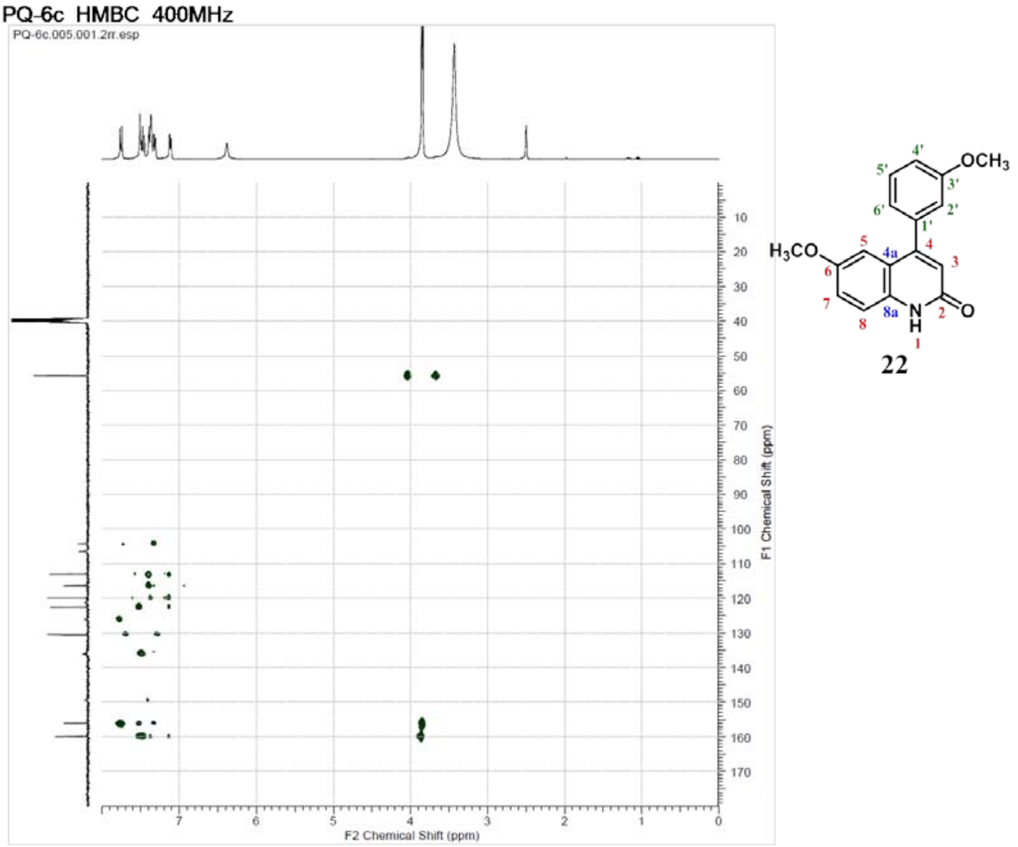


**Figure S19.** HMBC Spectra of compound **6-Methoxy-4-(3-methoxyphenyl)quinolin-2(1*H*)-one (22)** was recorded in DMSO-*d*6 (400MHz)

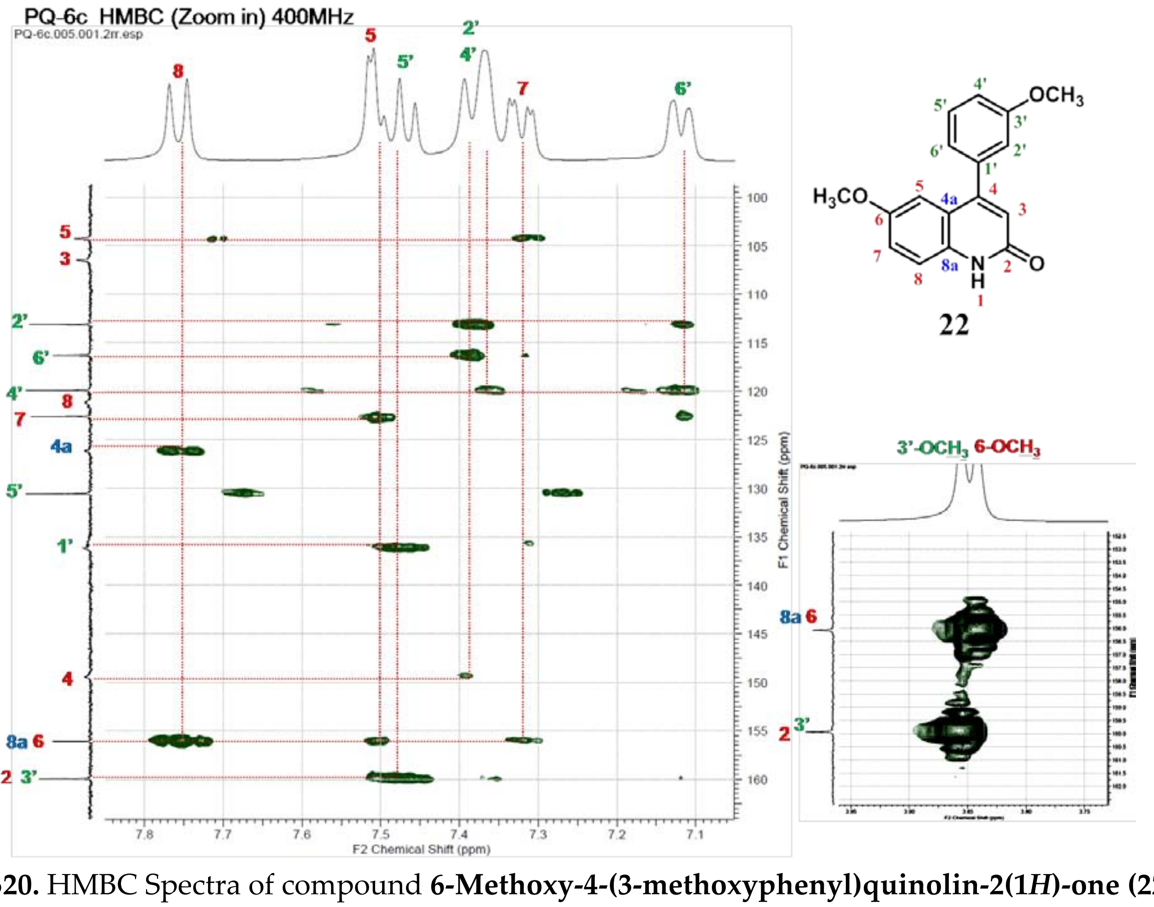


**Figure S20.** HMBC Spectra of compound **6-Methoxy-4-(3-methoxyphenyl)quinolin-2(1*H*)-one (22)** was recorded in DMSO-*d*6 (400MHz Zoom in)

792, 840, 1028, 1085, 1205, 1224, 1269, 1294, 1382, 1462, 1506, 1537, 1581, 1598 (C=O), 2763-3248 (C-H).

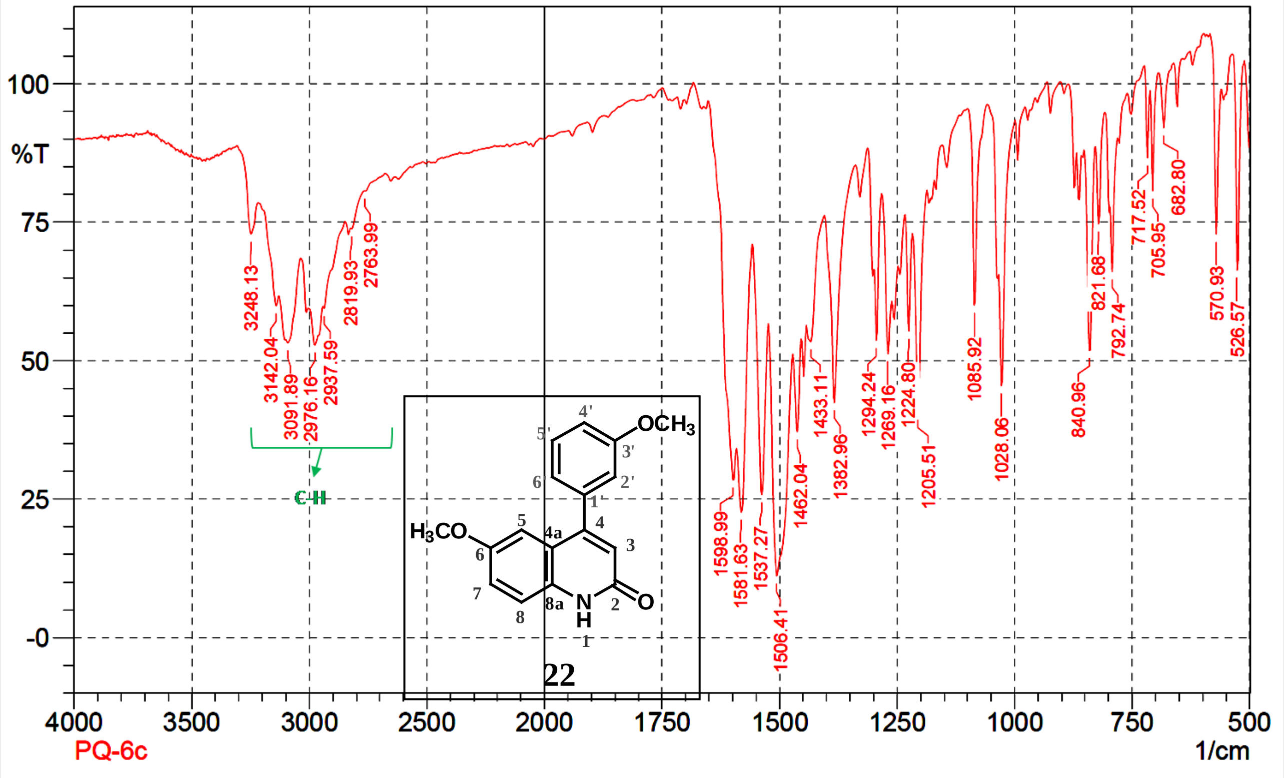


**Figure S21.** IR Spectra of compound **6-Methoxy-4-(3-methoxyphenyl)quinolin-2(1*H*)-one (22)**

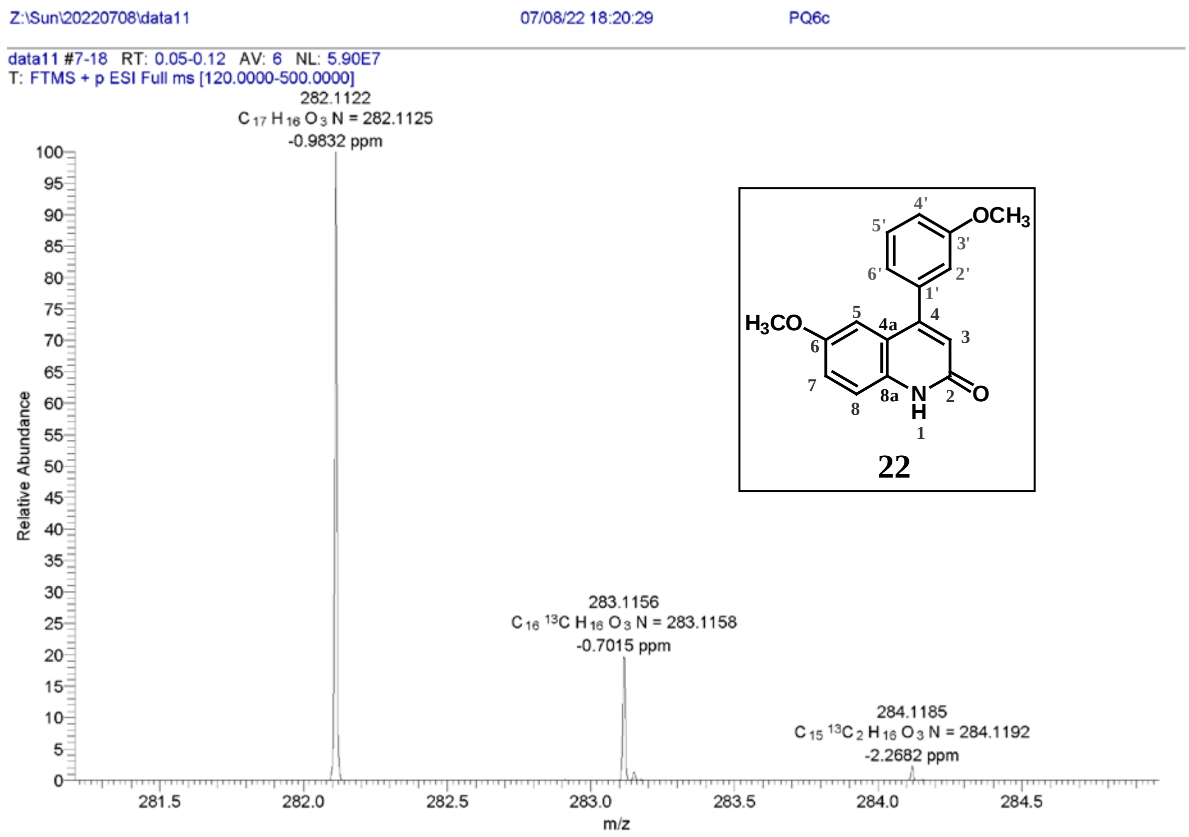


**Figure S22.** Mass Spectra of compound **6-Methoxy-4-(3-methoxyphenyl)quinolin-2(1*H*)-one (22)**

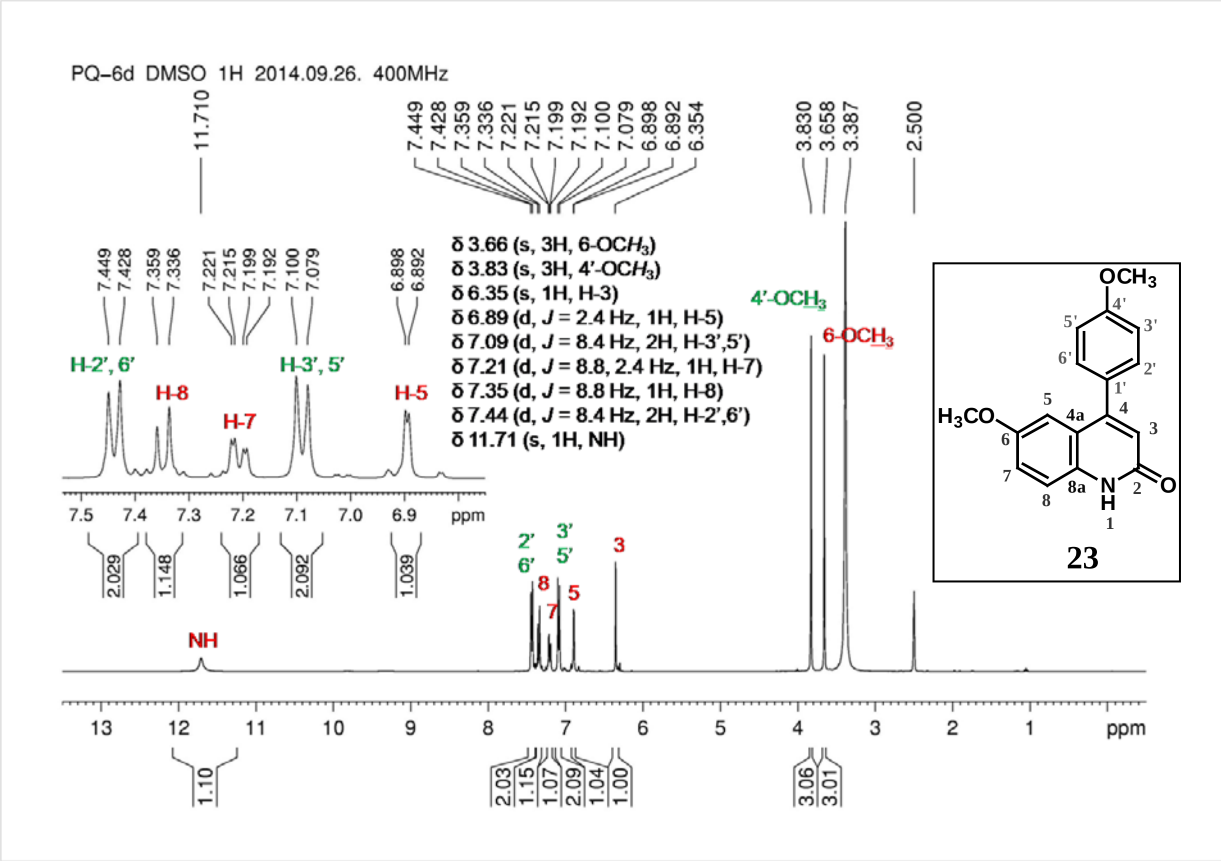


**Figure S23.** $^{1}$H-NMR Spectra of compound **6-Methoxy-4-(4-methoxyphenyl)quinolin-2(1*H*)-one (23)** was recorded in DMSO-d6 (400MHz)

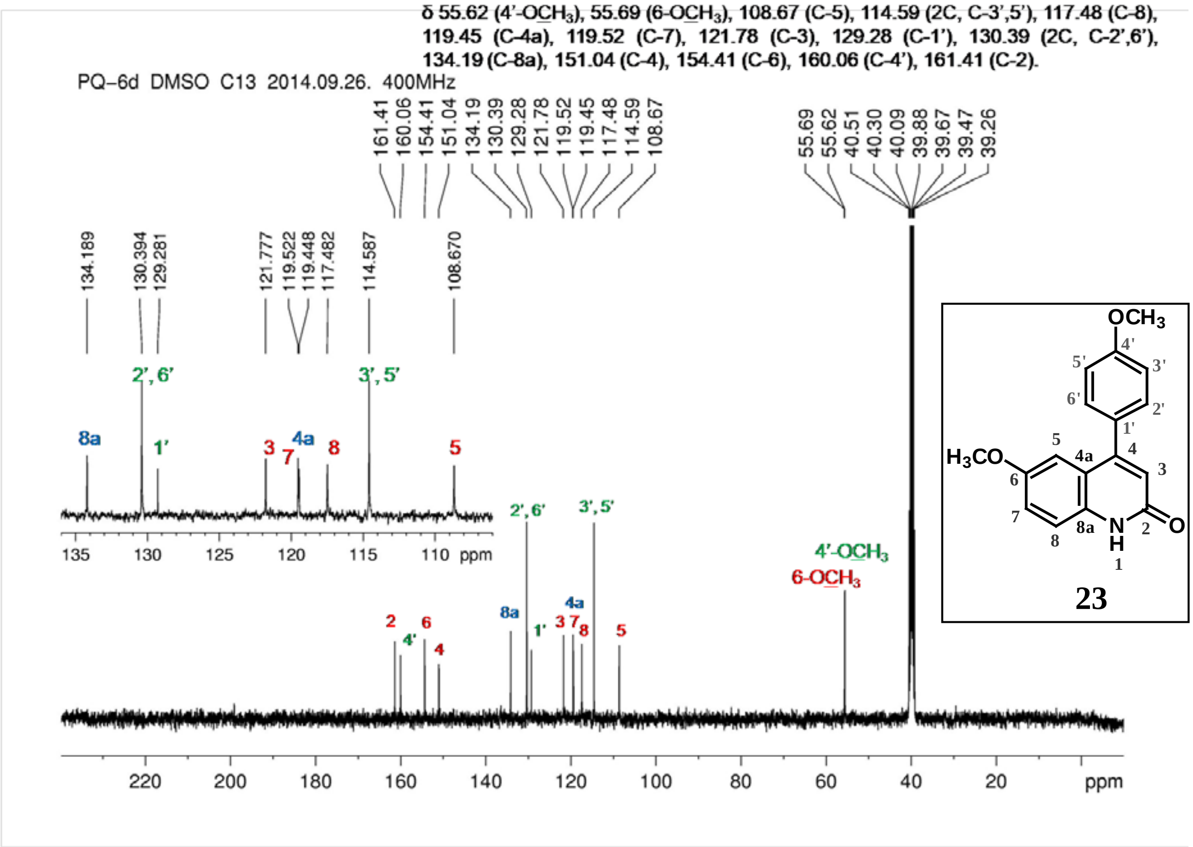


**Figure S24.**$^{13}$C-NMR Spectra of compound **6-Methoxy-4-(4-methoxyphenyl)quinolin-2(1*H*)-one (23)** was recorded in DMSO-*d6* (100MHz)

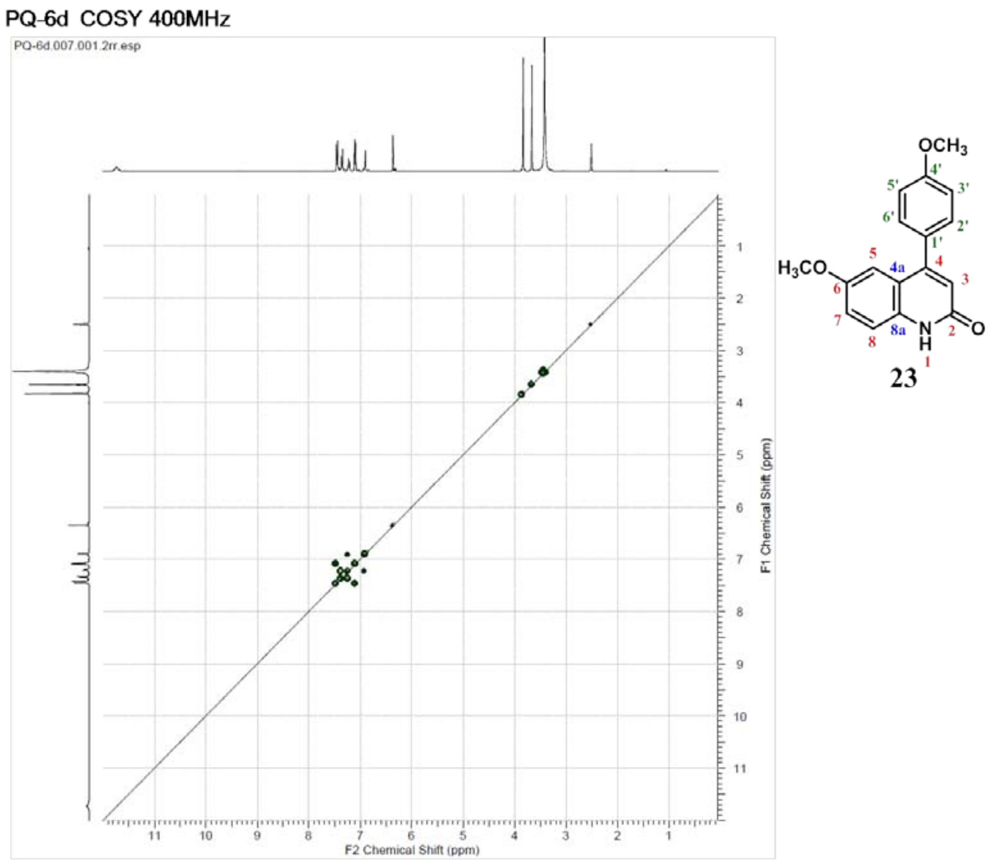


**Figure S25.** $^1H$-$^1H$ COSY Spectra of compound **6-Methoxy-4-(4-methoxyphenyl)quinolin-2(1*H*)-one (23)** was recorded in DMSO-$d_6$ (400MHz)

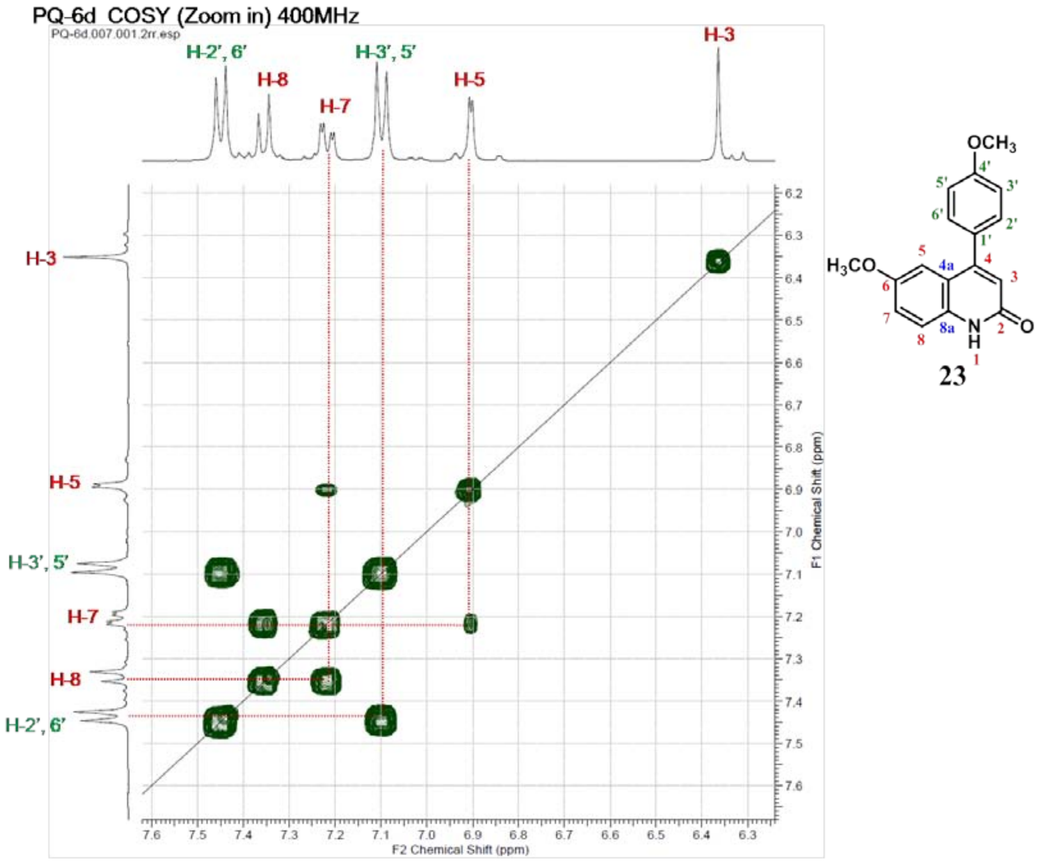


**Figure S26.** $^1H$-$^1H$ COSY Spectra of compound **6-Methoxy-4-(4-methoxyphenyl)quinolin-2(1*H*)-one (23)** was recorded in DMSO-$d_6$ (400MHz Zoom in)

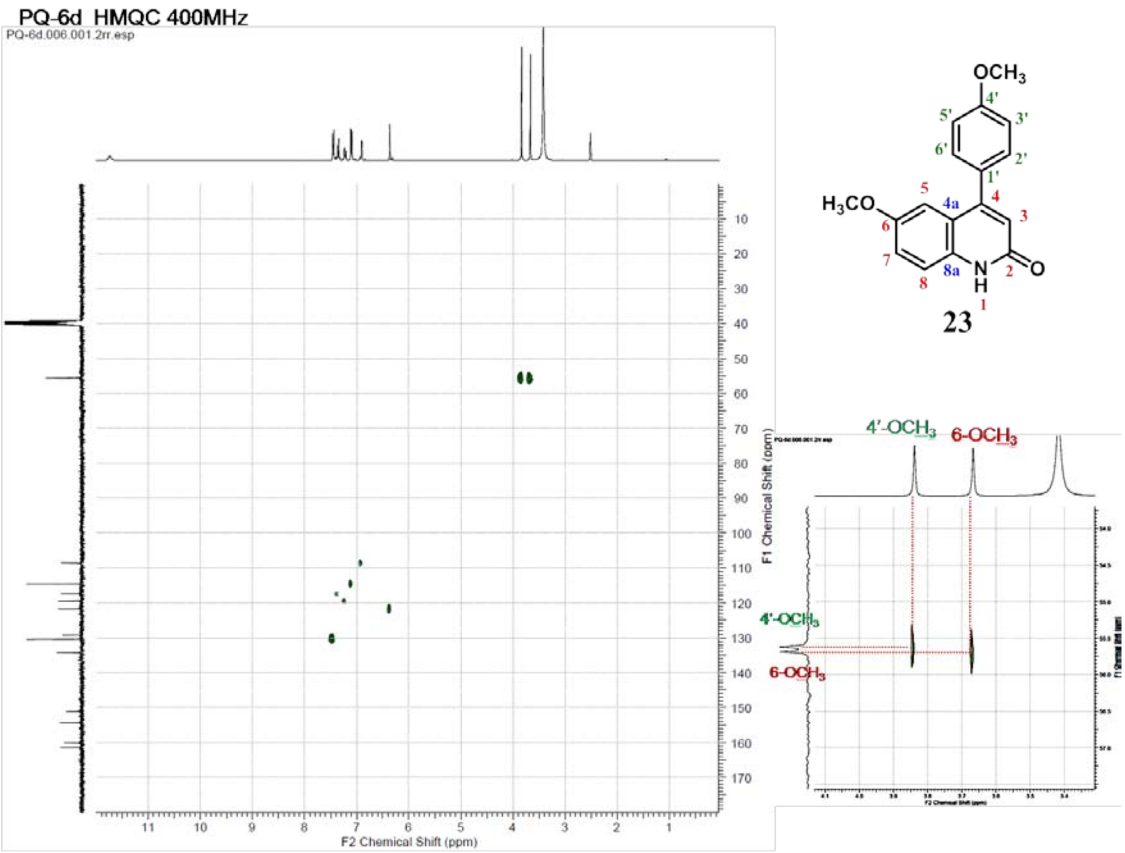


**Figure S27.** HMQC Spectra of compound **6-Methoxy-4-(4-methoxyphenyl)quinolin-2(1*H*)-one (23)** was recorded in DMSO-*d*6 (400MHz)

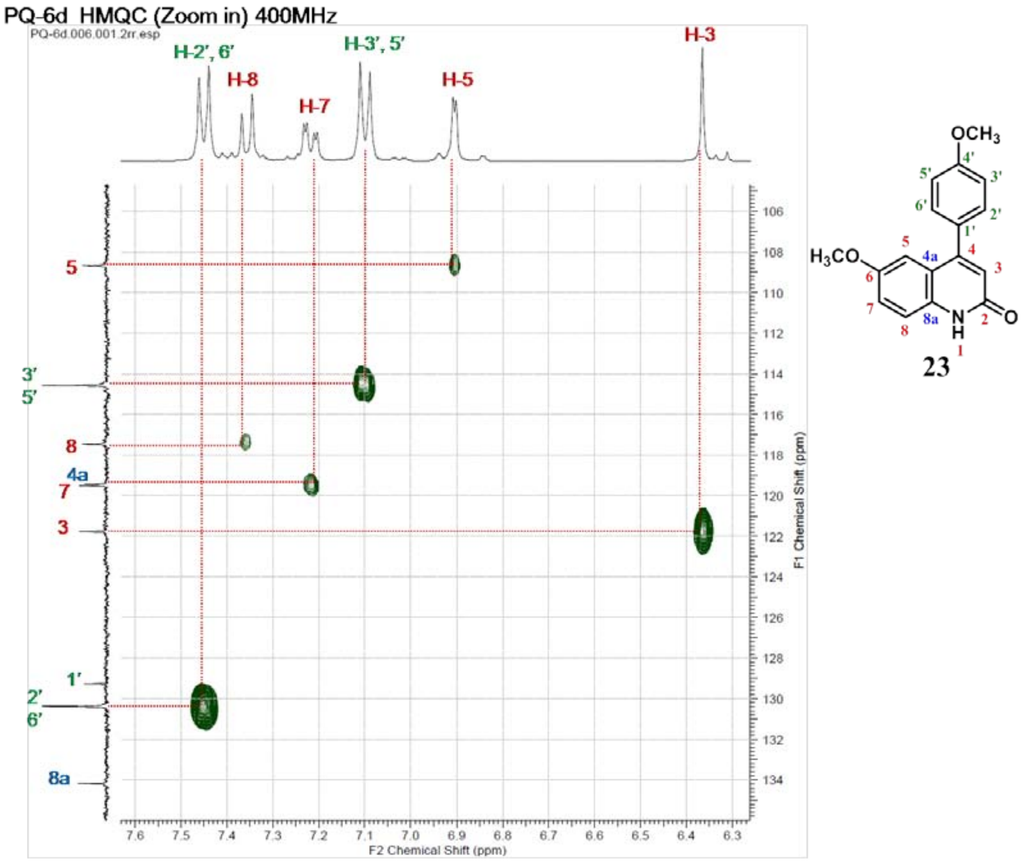


**Figure S28.** HMQC Spectra of compound **6-Methoxy-4-(4-methoxyphenyl)quinolin-2(1*H*)-one (23)** was recorded in DMSO-*d*6 (400MHz Zoom in)

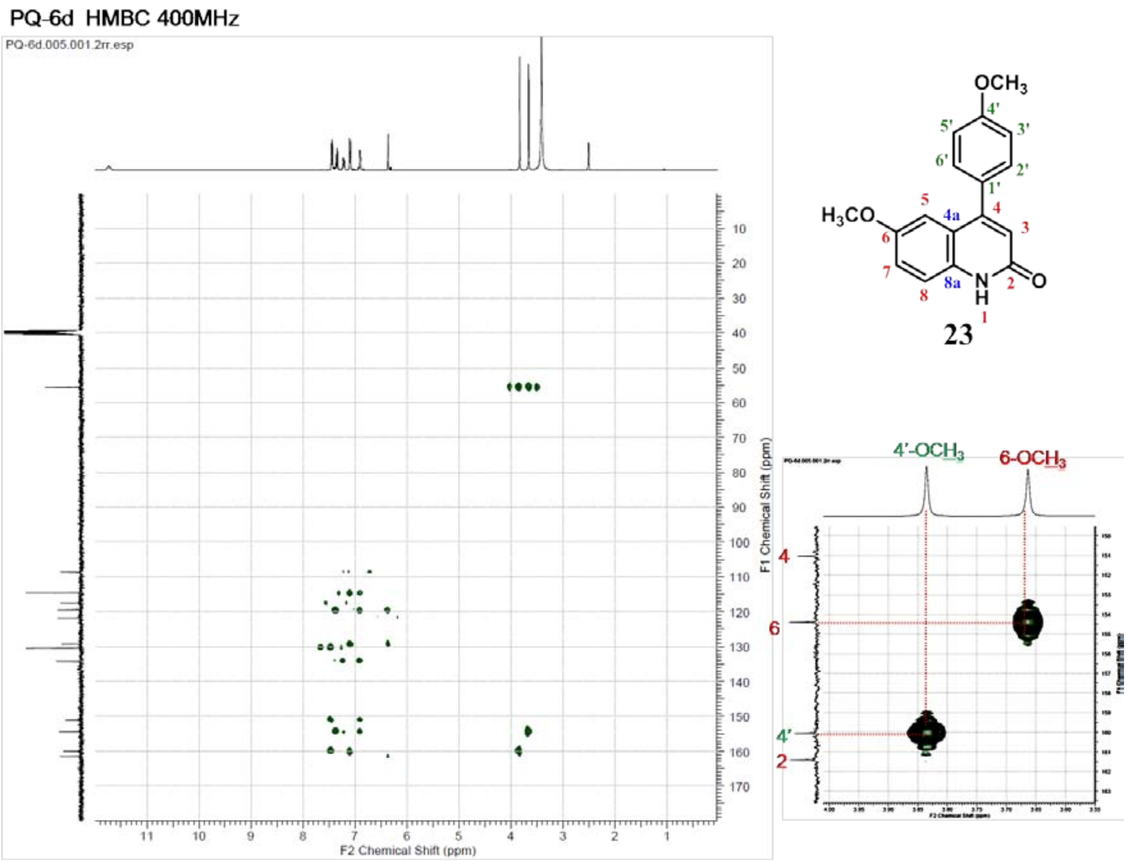


**Figure S29.** HMBC Spectra of compound **6-Methoxy-4-(4-methoxyphenyl)quinolin-2(1*H*)-one (23)** was recorded in DMSO-*d*6 (400MHz)

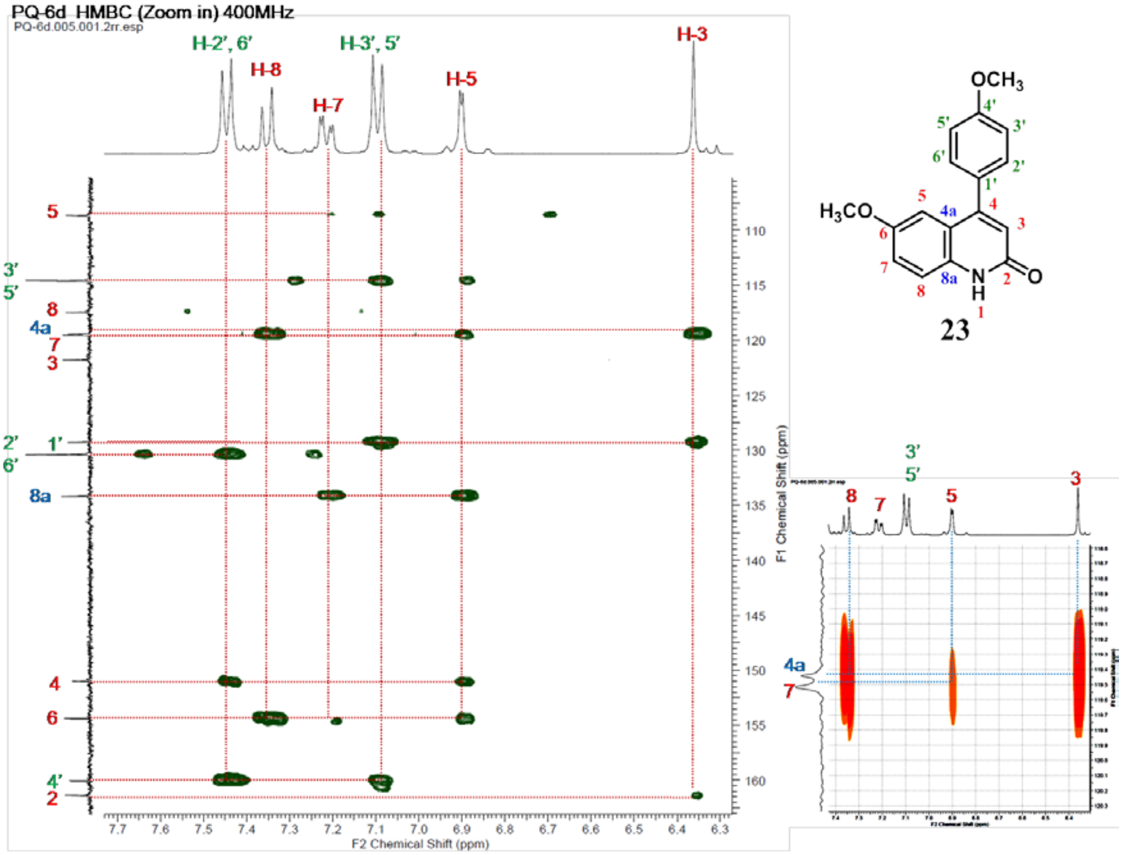


**Figure S30.** HMBC Spectra of compound **6-Methoxy-4-(4-methoxyphenyl)quinolin-2(1*H*)-one (23)** was recorded in DMSO-*d*6 (400MHz Zoom in)

628, 717, 804, 842, 875, 1001, 1041, 1118, 1178, 1217, 1263, 1276, 1300, 1433, 1500, 1612, 1649 (C=O), 2735-3142 (C-H), 3415 (N-H).

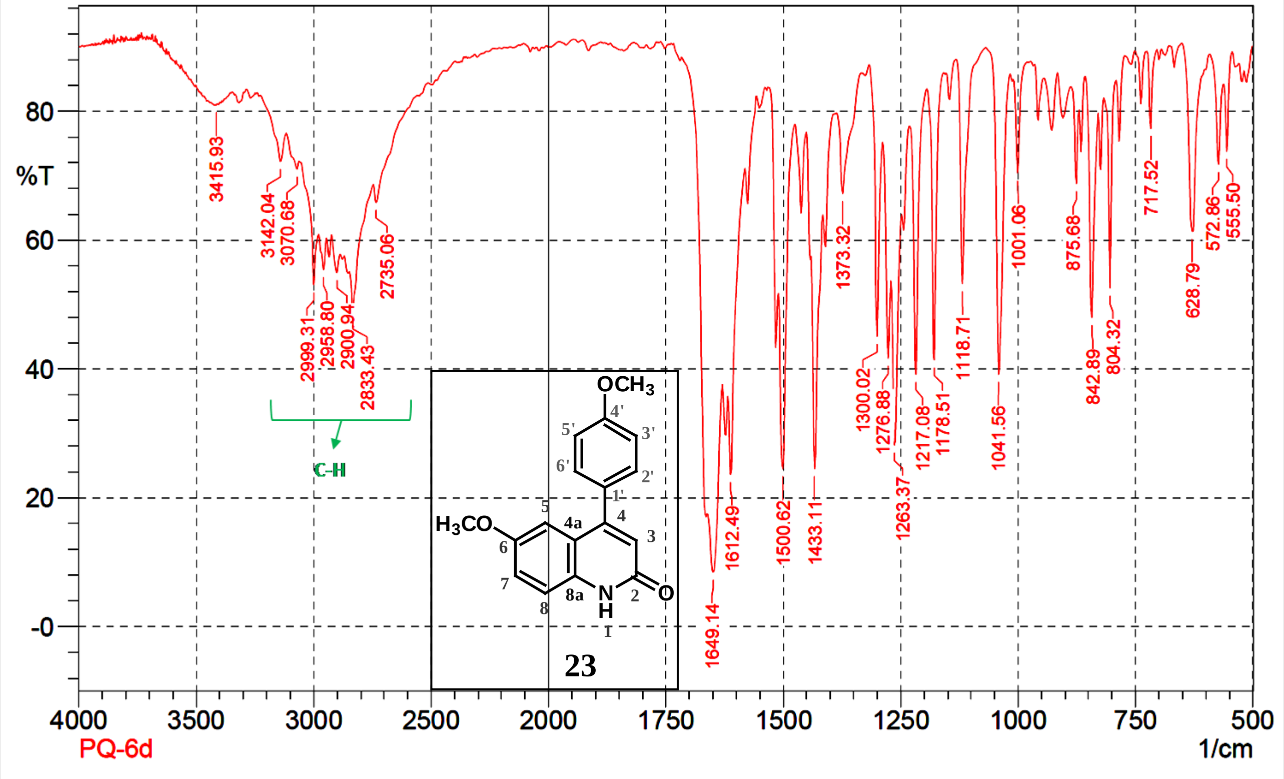


**Figure S31.** IR Spectra of compound **6-Methoxy-4-(4-methoxyphenyl)quinolin-2(1*H*)-one (23)**

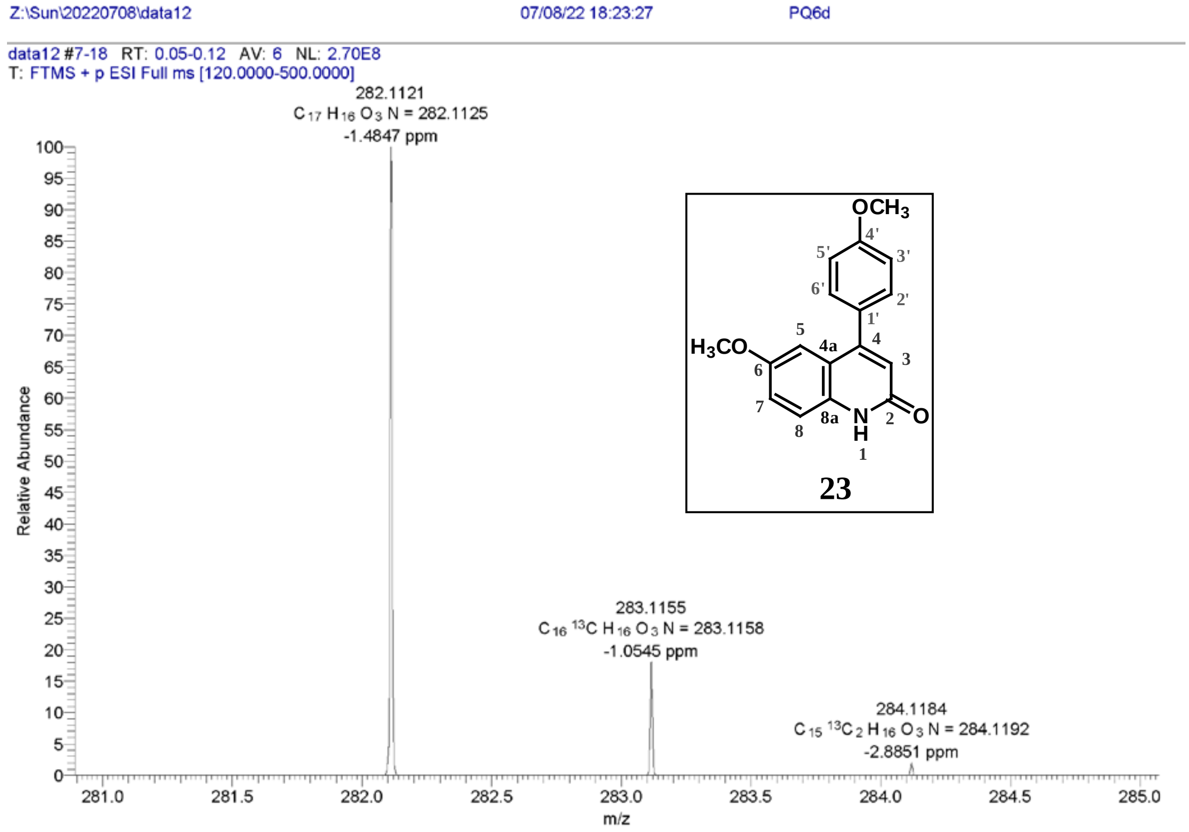


**Figure S32.** Mass Spectra of compound **6-Methoxy-4-(4-methoxyphenyl)quinolin-2(1*H*)-one (23)**

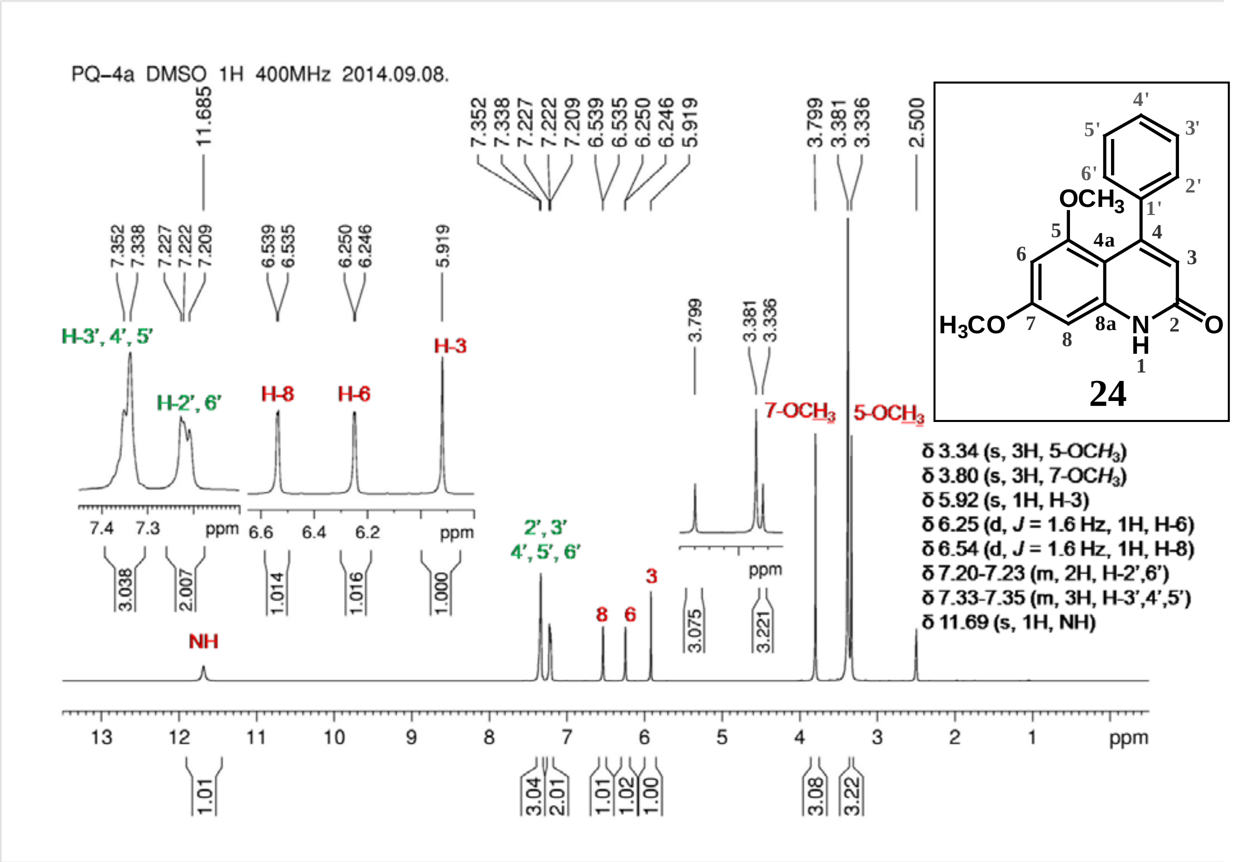


**Figure S33.** $^{1}$H-NMR Spectra of compound **5,7-Dimethoxy-4-phenylquinolin-2(1*H*)-one (24)** was recorded in DMSO-d6 (400MHz)

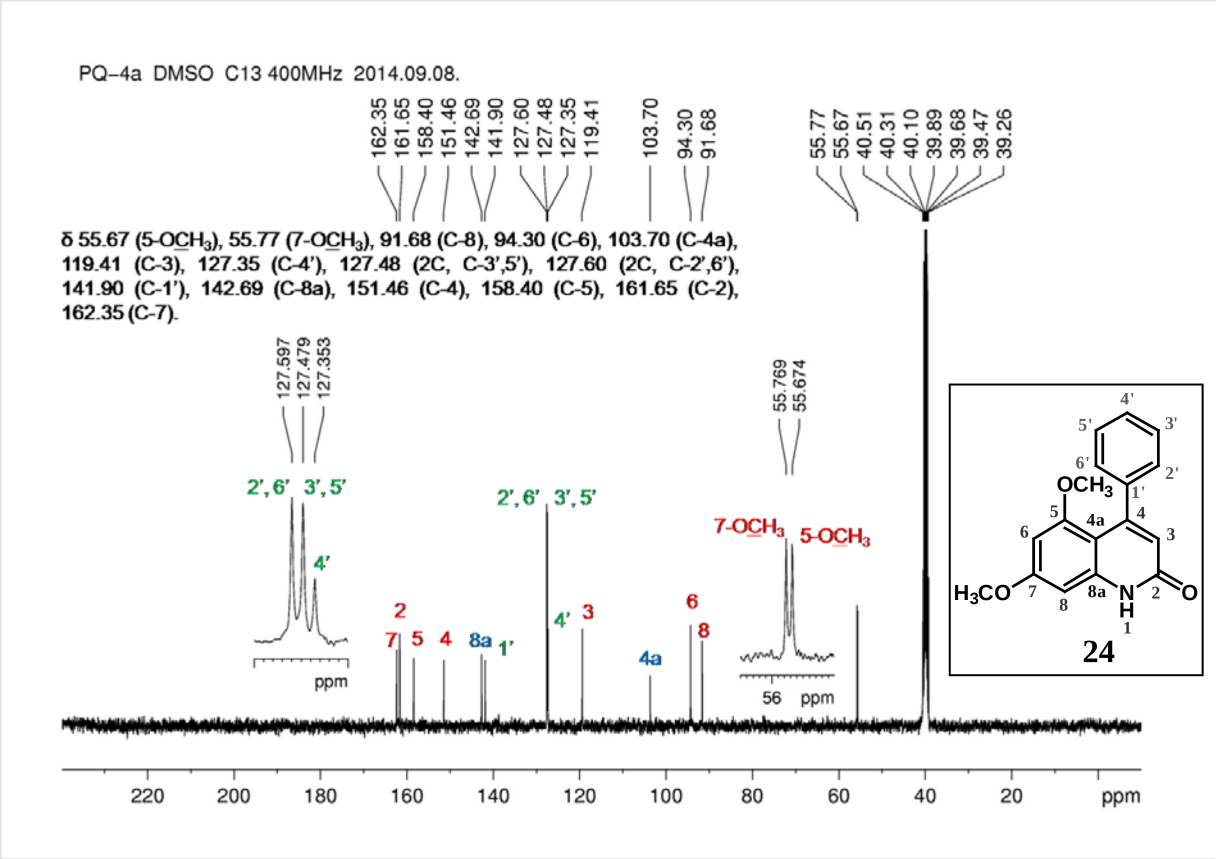


**Figure S34.** $^{13}$C-NMR Spectra of compound **5,7-Dimethoxy-4-phenylquinolin-2(1*H*)-one (24)** was recorded in DMSO-*d6* (100MHz)

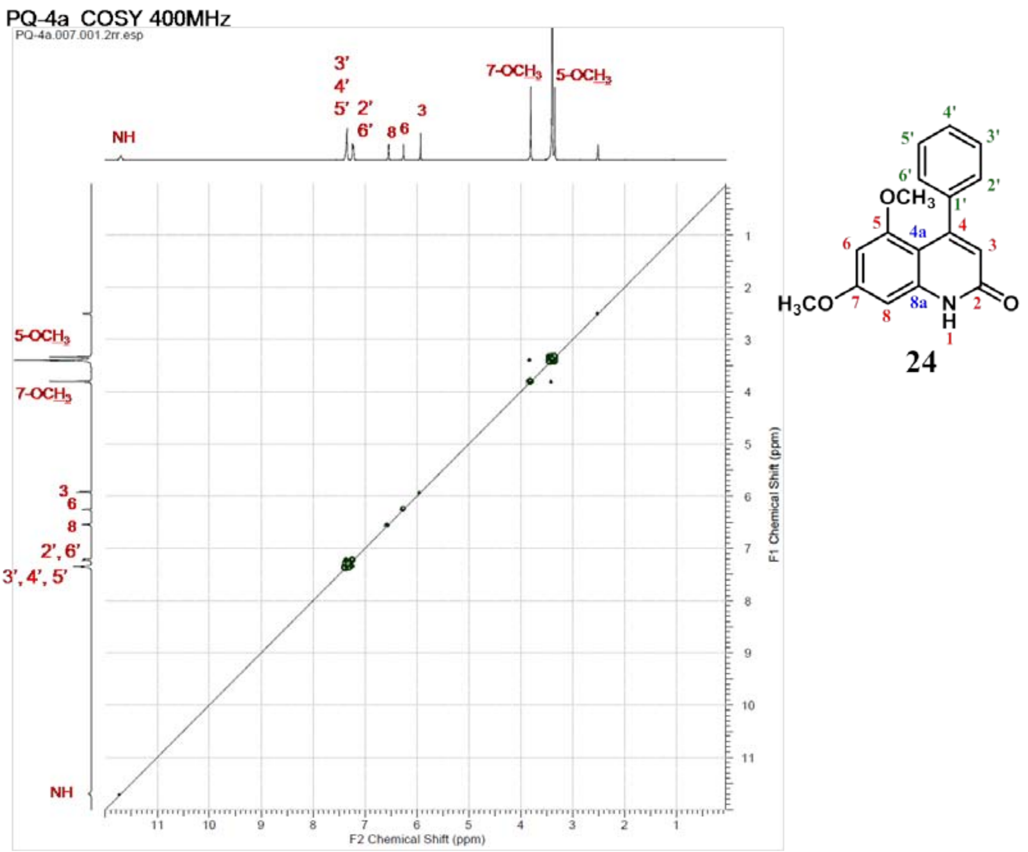


**Figure S35.** $^1$H-$^1$H COSY Spectra of compound **5,7-Dimethoxy-4-phenylquinolin-2(1*H*)-one (24)** was recorded in DMSO-$d_6$ (400MHz)

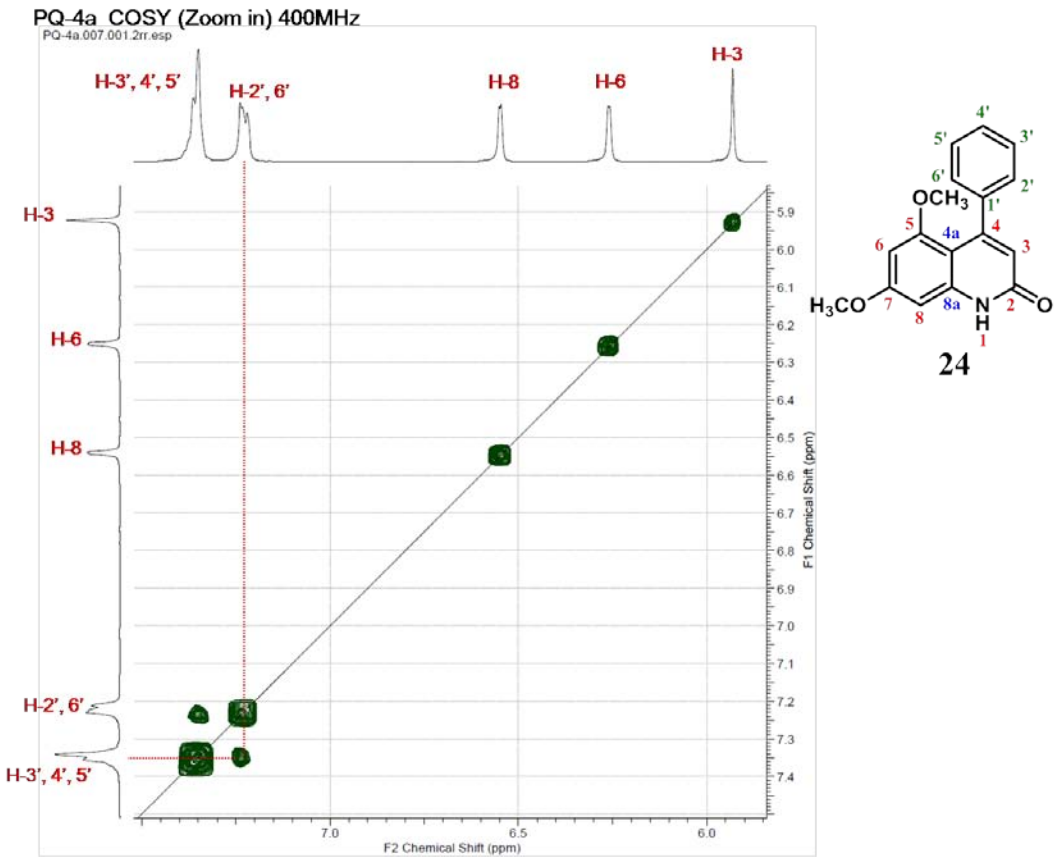


**Figure S36.** $^1$H-$^1$H COSY Spectra of compound **5,7-Dimethoxy-4-phenylquinolin-2(1*H*)-one (24)** was recorded in DMSO-$d_6$ (400MHz Zoom in)

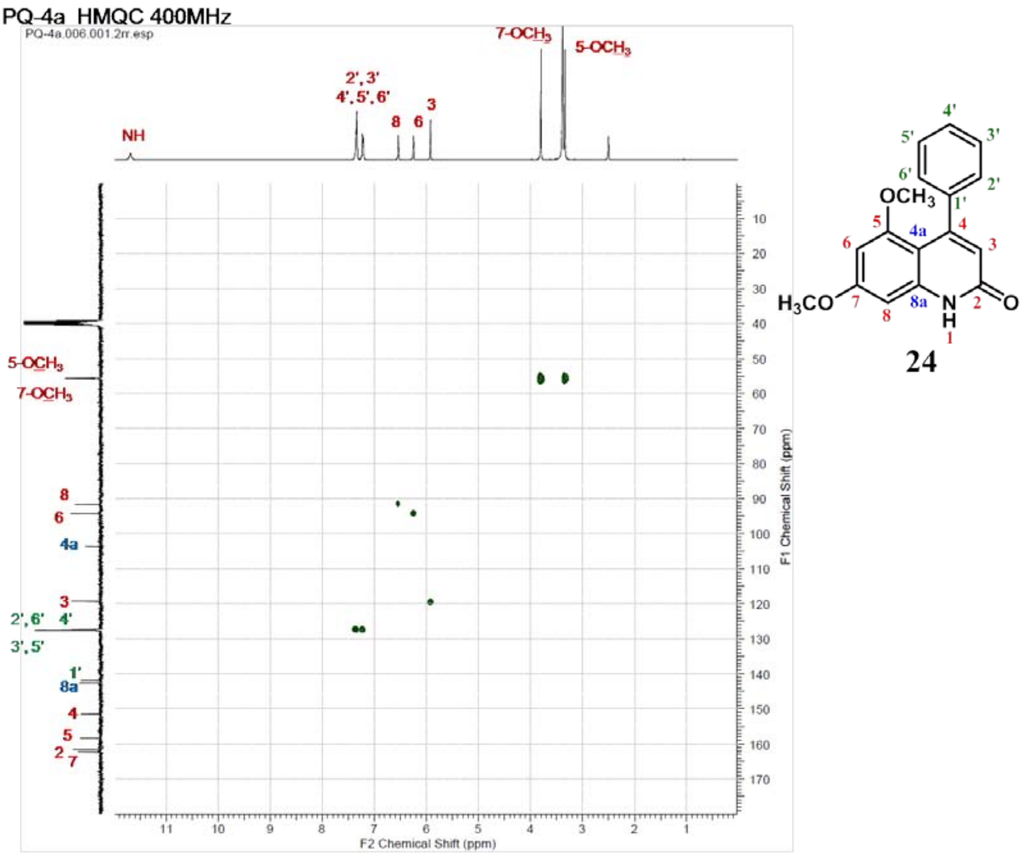


**Figure S37.** HMQC Spectra of compound **5,7-Dimethoxy-4-phenylquinolin-2(1*H*)-one (24)** was recorded in DMSO-$d_6$ (400MHz)

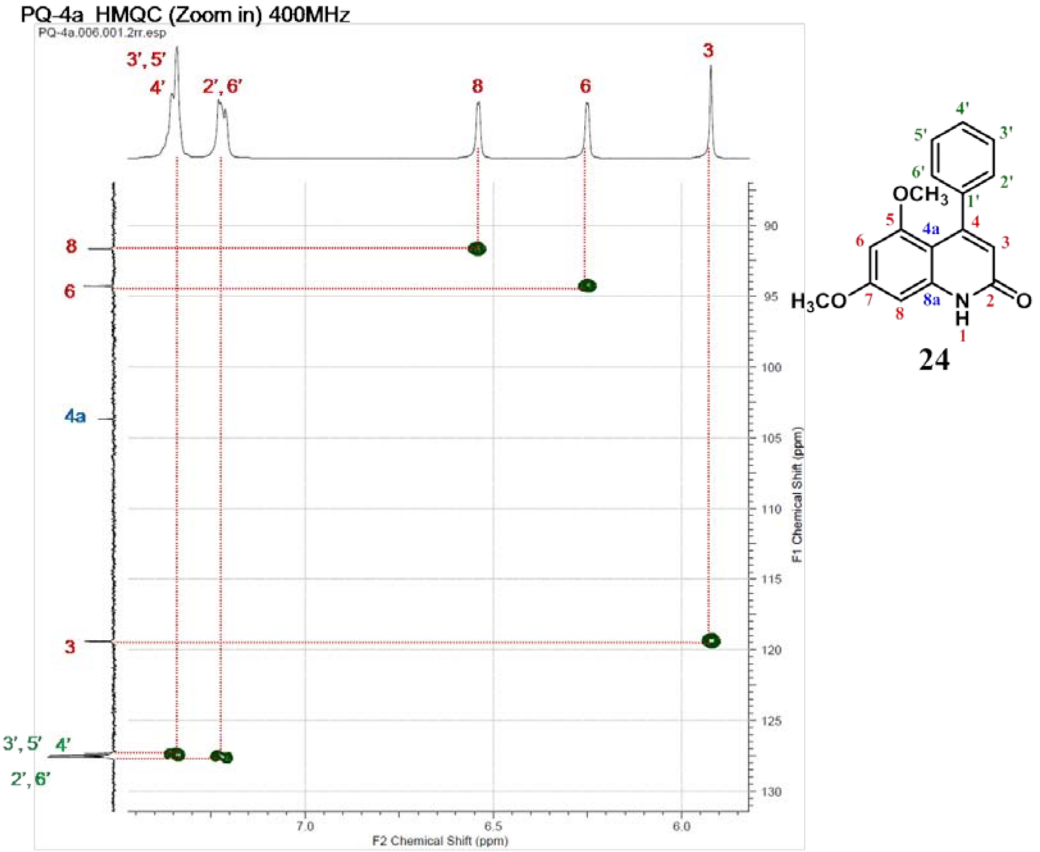


**Figure S38.** HMQC Spectra of compound **5,7-Dimethoxy-4-phenylquinolin-2(1*H*)-one (24)** was recorded in DMSO-$d_6$ (400MHz Zoom in)

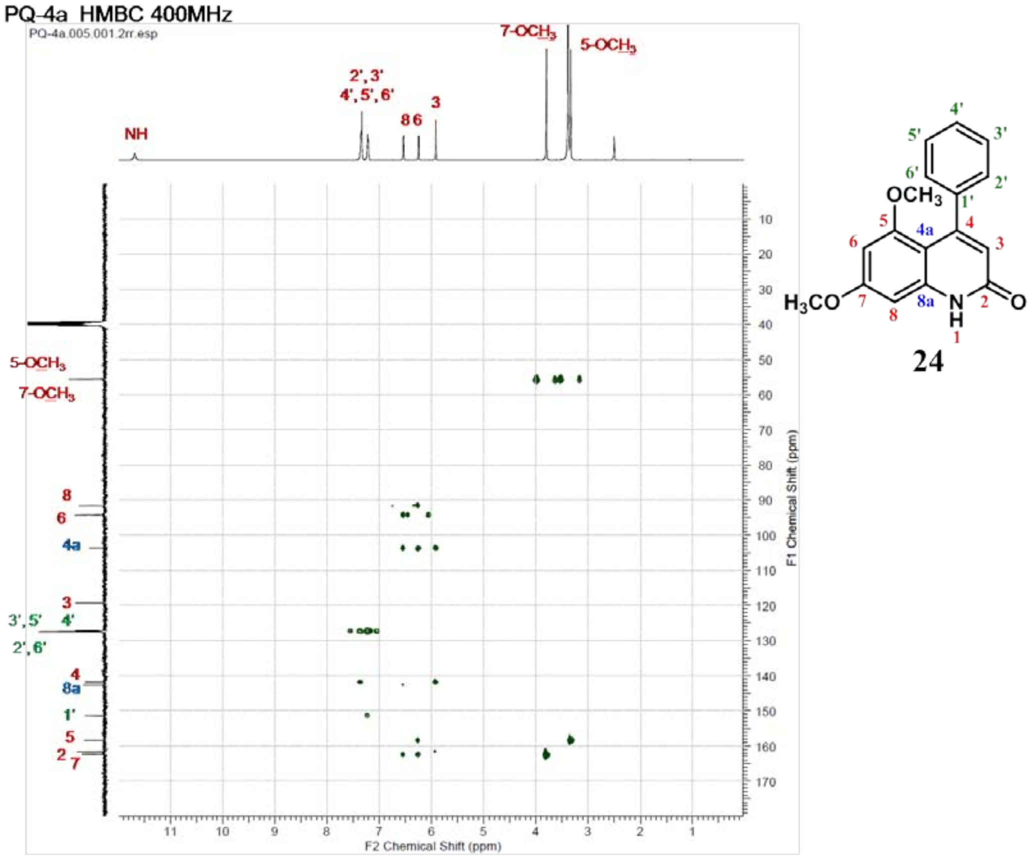

**Figure S39.** HMBC Spectra of compound **5,7-Dimethoxy-4-phenylquinolin-2(1*H*)-one (24)** was recorded in DMSO-$d_6$ (400MHz)

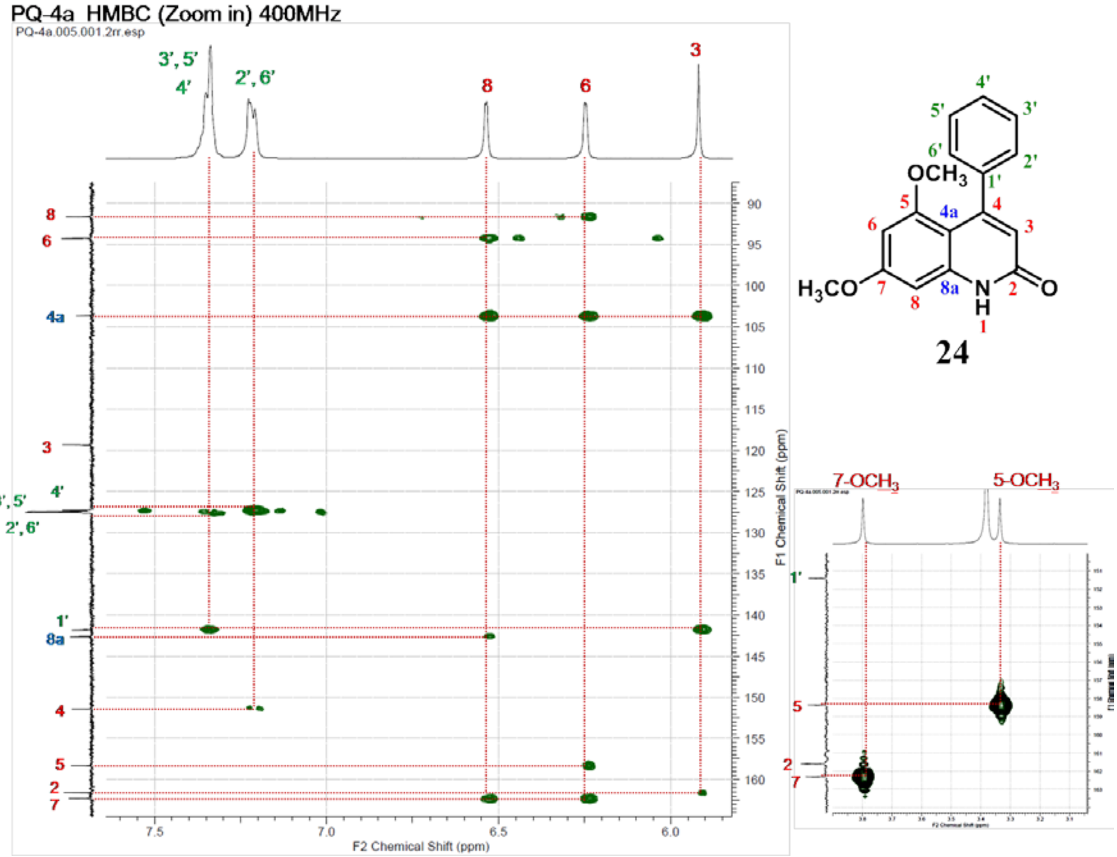

**Figure S40.** HMBC Spectra of compound **5,7-Dimethoxy-4-phenylquinolin-2(1*H*)-one (24)** was recorded in DMSO-$d_6$ (400MHz Zoom in)

698, 759, 817, 985, 1058, 1139, 1166, 1209, 1228, 1276, 1386, 1409, 1438, 1512, 1537, 1597, 1627, 1654 (C=O), 2812-3059 (C-H), 3383 (N-H), 3637.

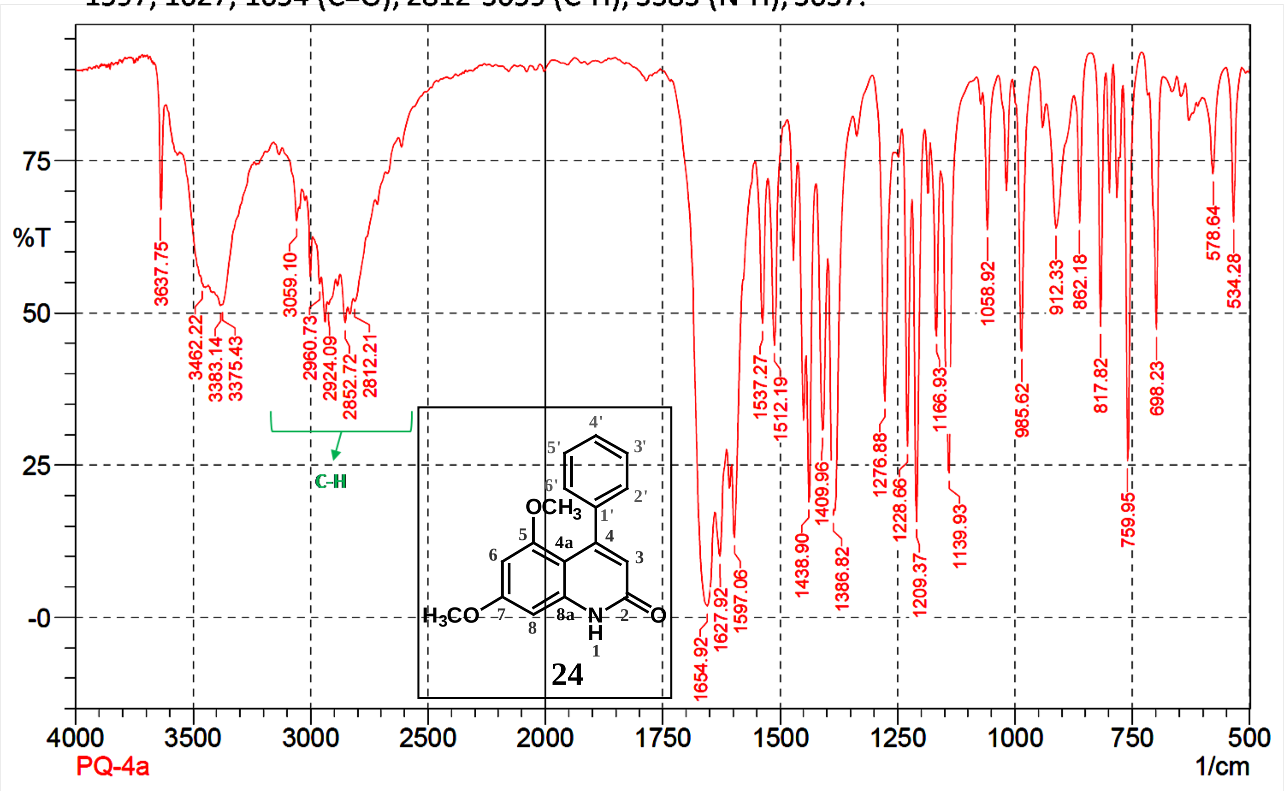


**Figure S41.** IR Spectra of compound **5,7-Dimethoxy-4-phenylquinolin-2(1*H*)-one (24)**

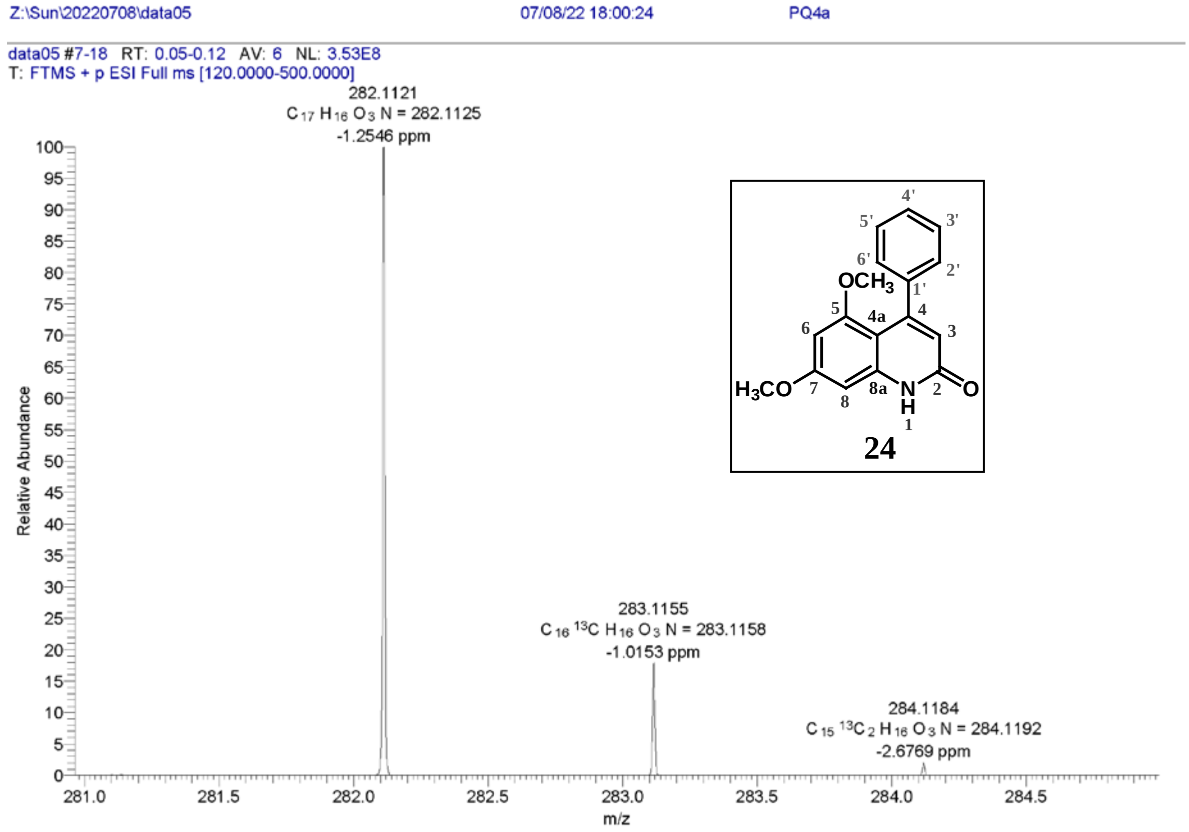


**Figure S42.** IR Spectra of compound **5,7-Dimethoxy-4-phenylquinolin-2(1*H*)-one (24)**

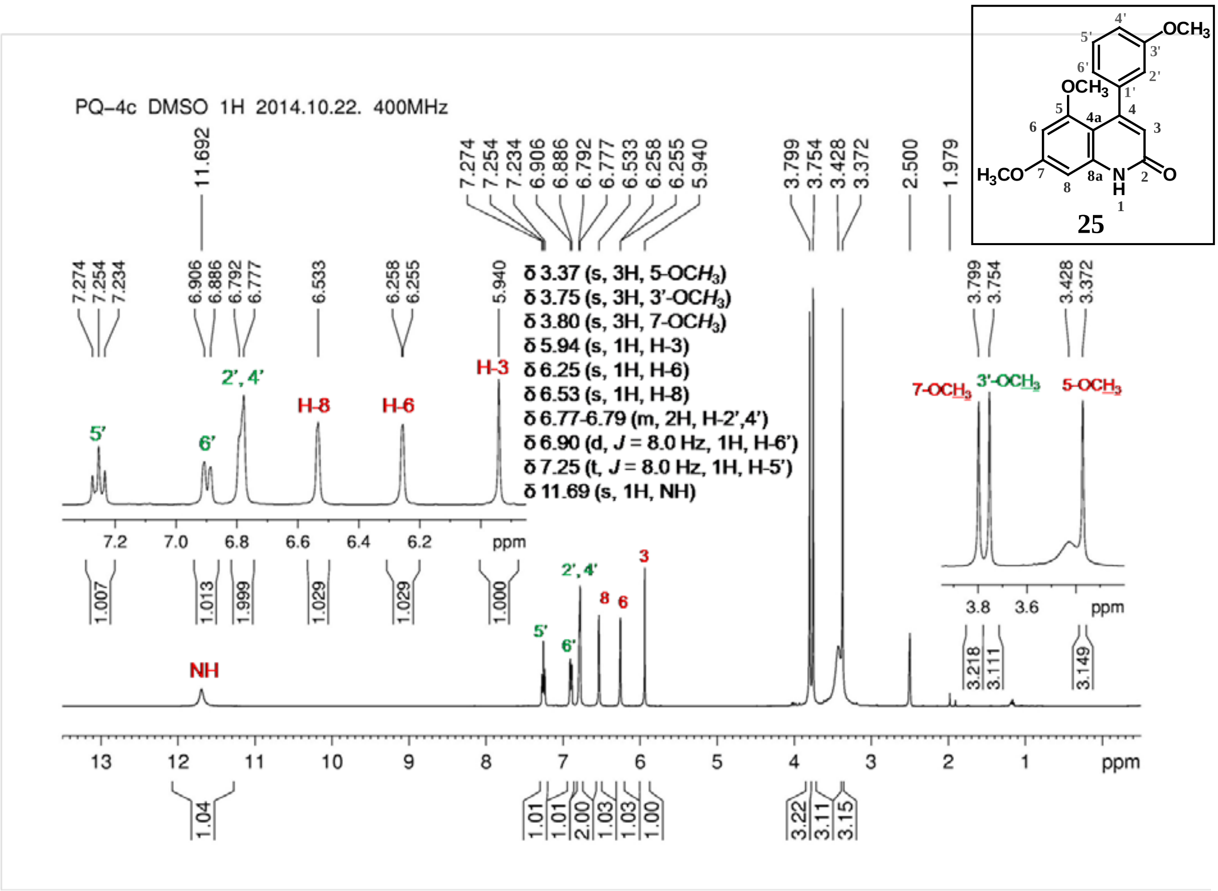


**Figure S43.** $^{1}$H-NMR Spectra of compound **5,7-Dimethoxy-4-(3-methoxyphenyl)quinolin-2(1*H*)-one (25)** was recorded in DMSO-d6 (400MHz)

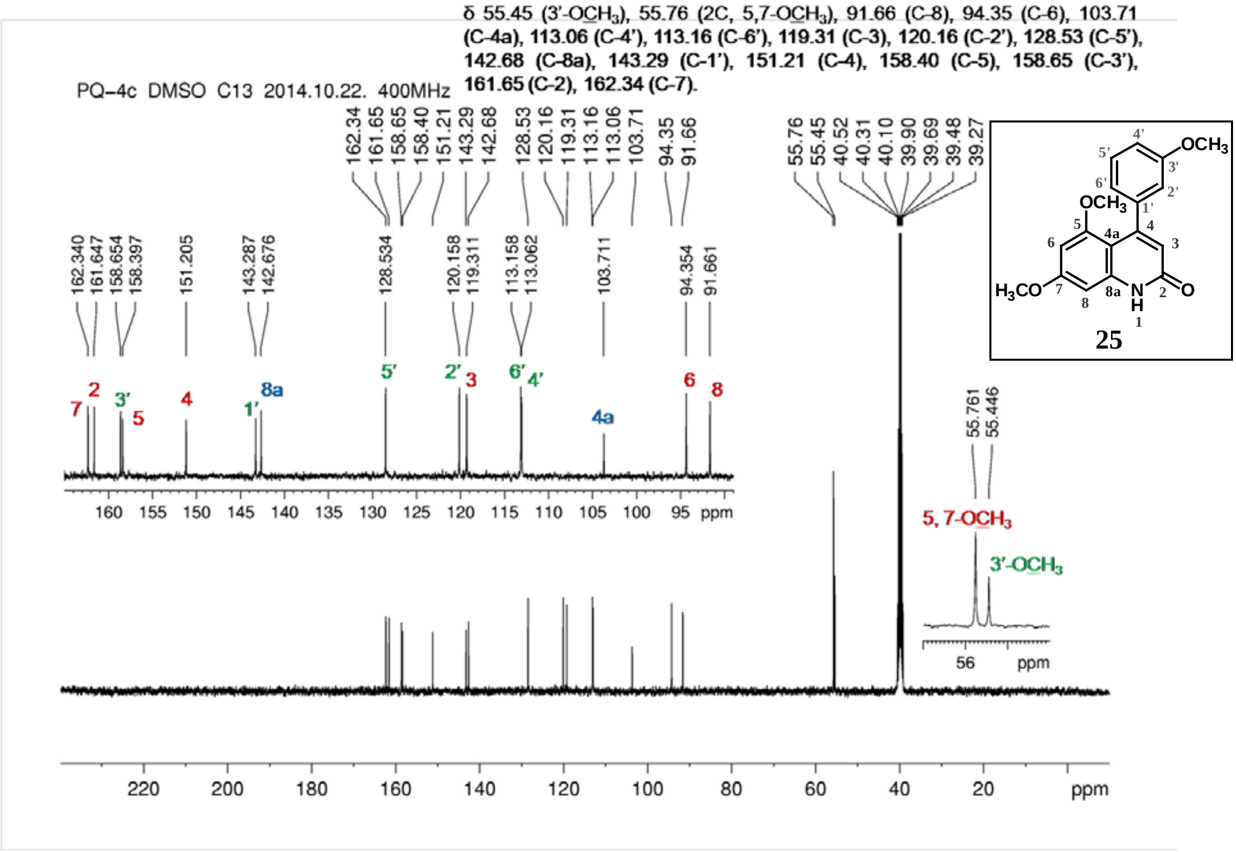


**Figure S44.**$^{13}$C-NMR Spectra of compound **5,7-Dimethoxy-4-(3-methoxyphenyl)quinolin-2(1*H*)-one (25)** was recorded in DMSO-*d6* (100MHz)

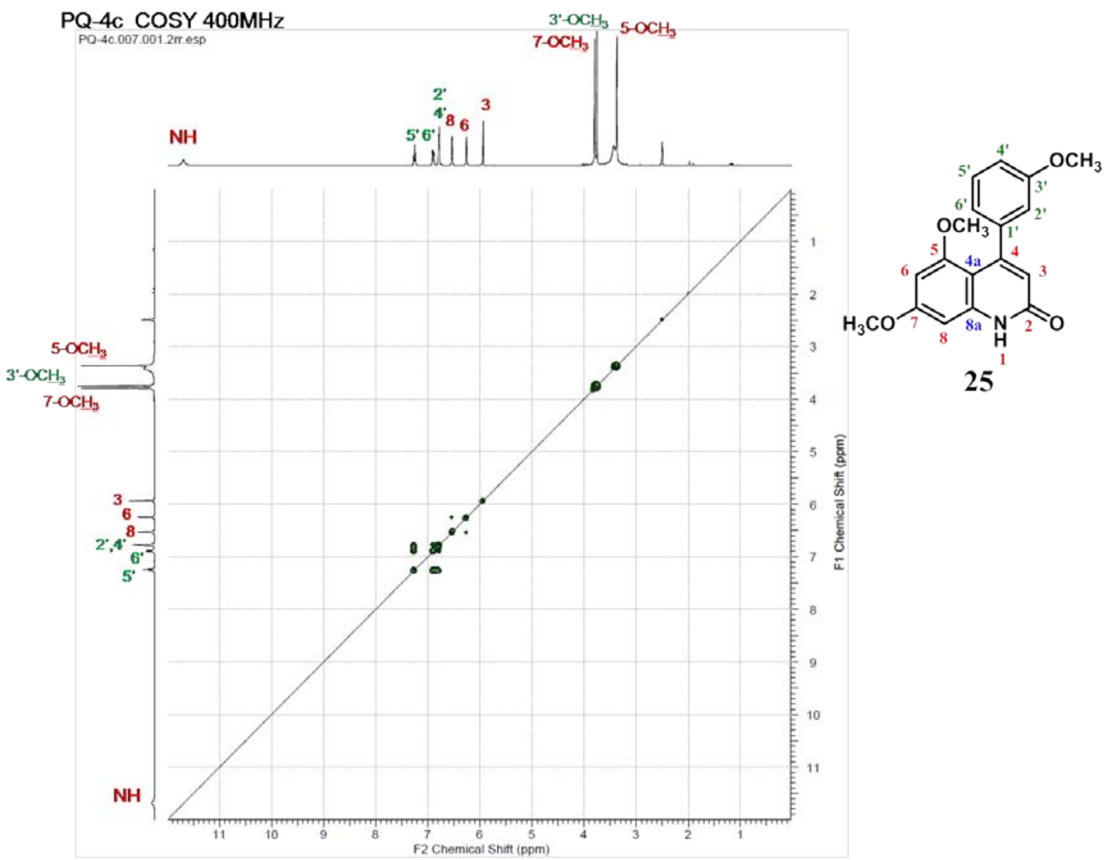


**Figure S45.** $^1$H-$^1$H COSY Spectra of compound **5,7-Dimethoxy-4-(3-methoxyphenyl)quinolin-2(1*H*)-one (25)** was recorded in DMSO-*d*$_6$ (400MHz)

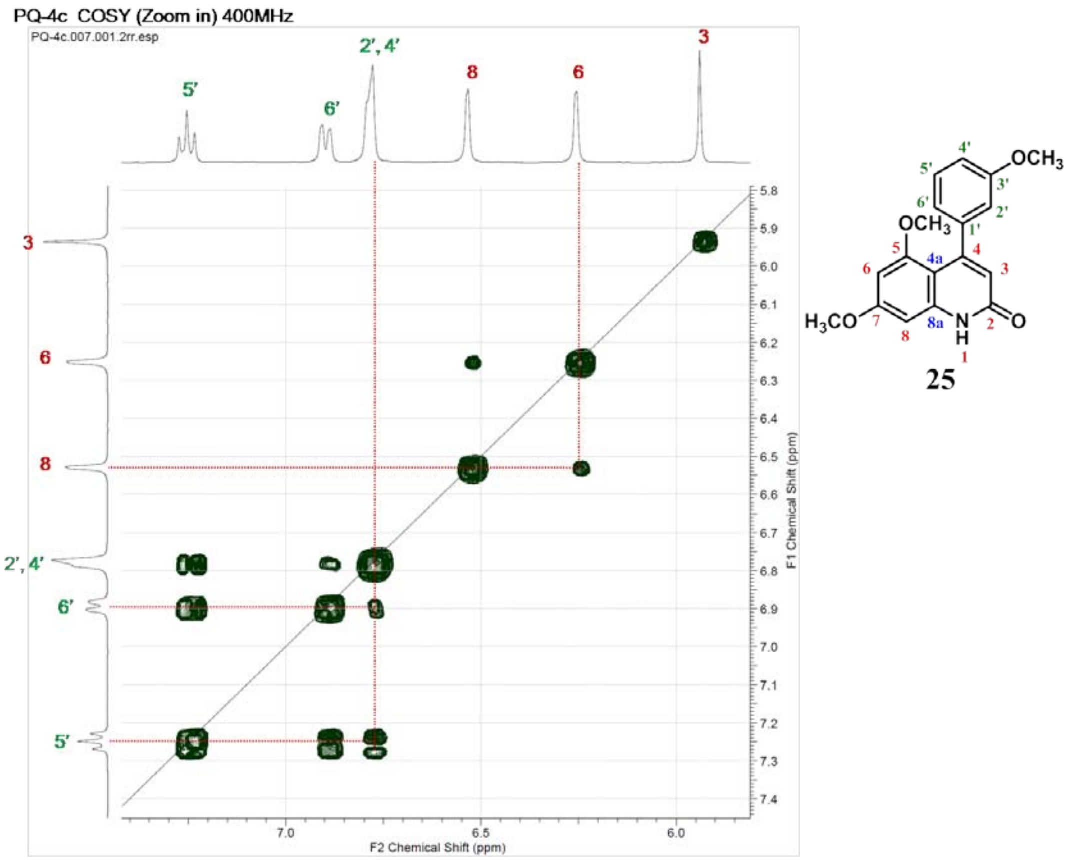


**Figure S46.** $^1$H-$^1$H COSY Spectra of compound **5,7-Dimethoxy-4-(3-methoxyphenyl)quinolin-2(1*H*)-one (25)** was recorded in DMSO-*d*$_6$ (400MHz Zoom in)

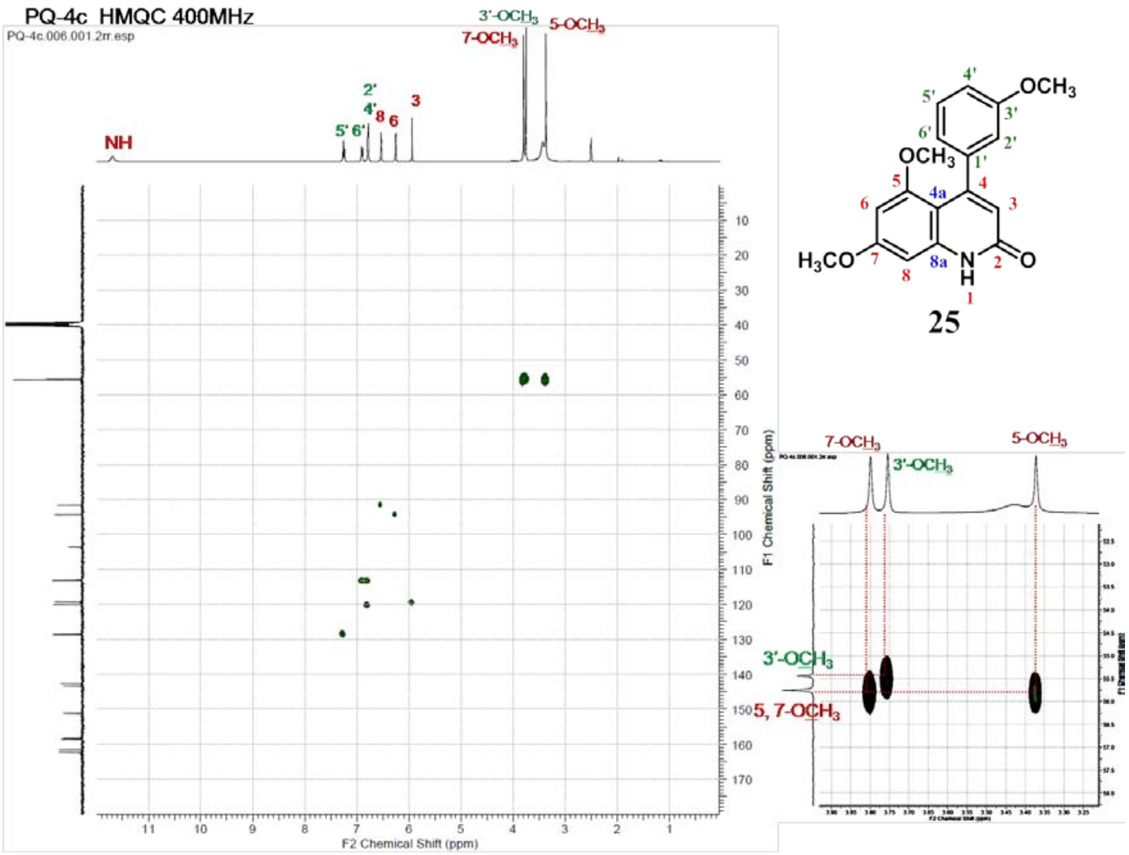


**Figure S47.** HMQC Spectra of compound **5,7-Dimethoxy-4-(3-methoxyphenyl)quinolin-2(1*H*)-one (25)** was recorded in DMSO-*d*6 (400MHz)

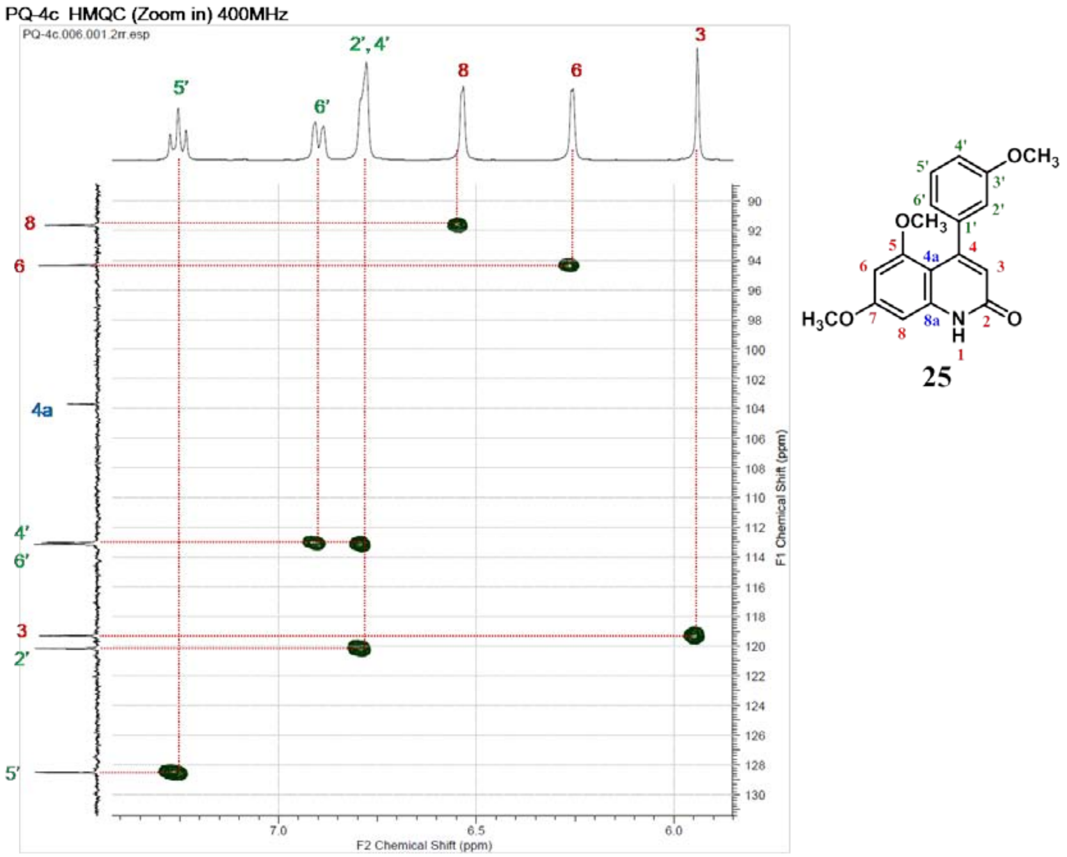


**Figure S48.** HMQC Spectra of compound **5,7-Dimethoxy-4-(3-methoxyphenyl)quinolin-2(1*H*)-one (25)** was recorded in DMSO-*d*6 (400MHz Zoom in)

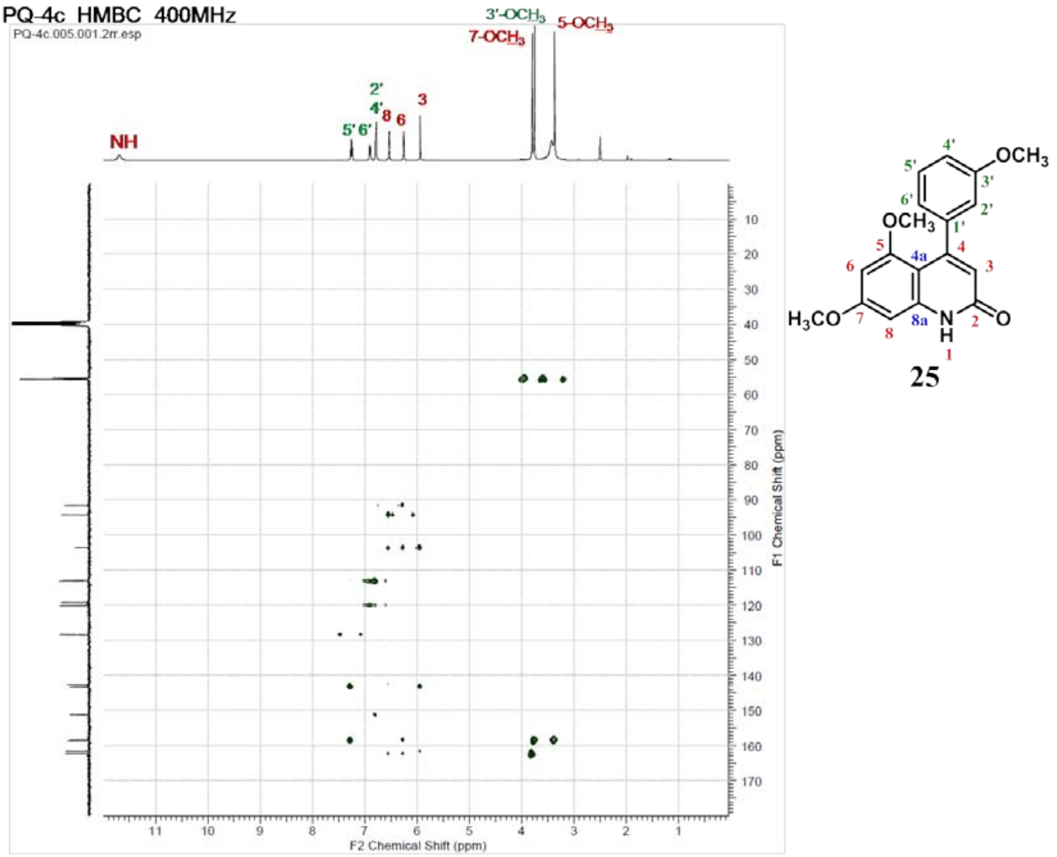


**Figure S49.** HMBC Spectra of compound **5,7-Dimethoxy-4-(3-methoxyphenyl)quinolin-2(1*H*)-one (25)** was recorded in DMSO-$d_6$ (400MHz)

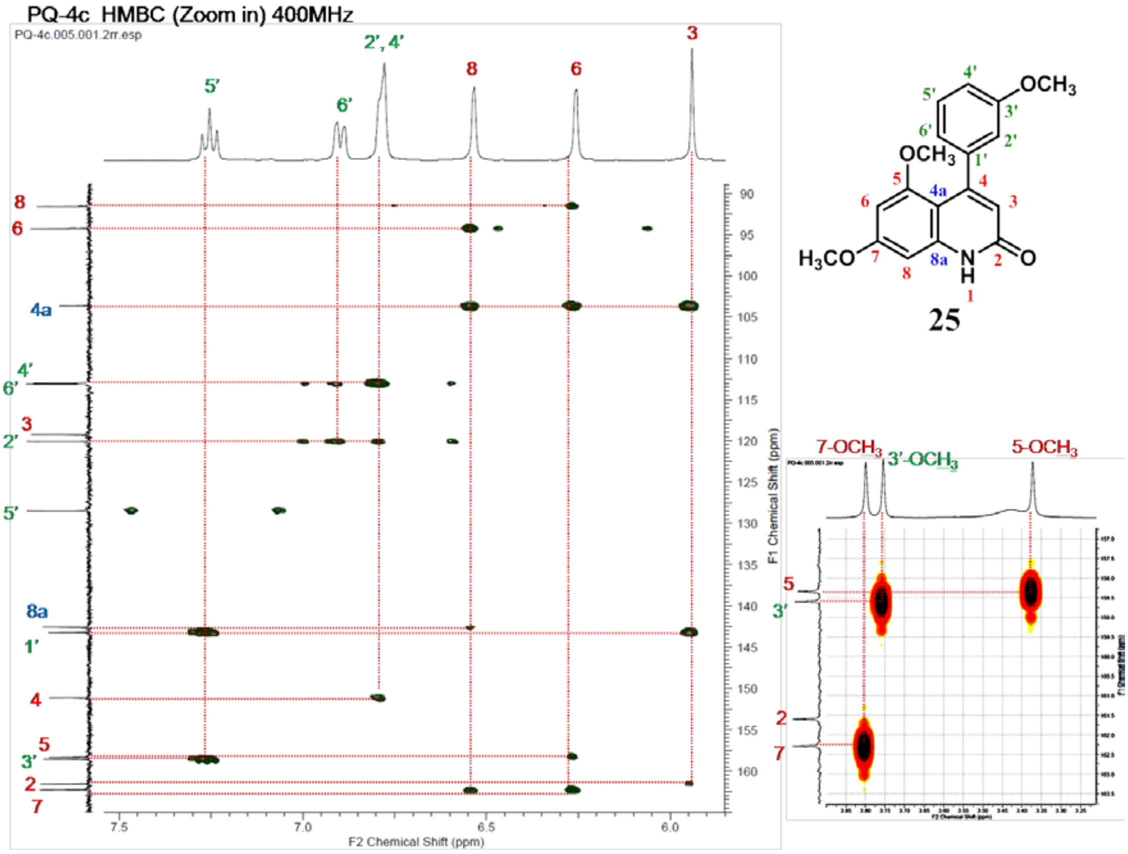


**Figure S50.** HMBC Spectra of compound **5,7-Dimethoxy-4-(3-methoxyphenyl)quinolin-2(1*H*)-one (25)** was recorded in DMSO-$d_6$ (400MHz Zoom in)

765, 806, 991, 1055, 1141, 1209, 1228, 1290, 1381, 1406, 1442, 1598, 1654 (C=O), 2615-3003 (C-H), 3338 (N-H).

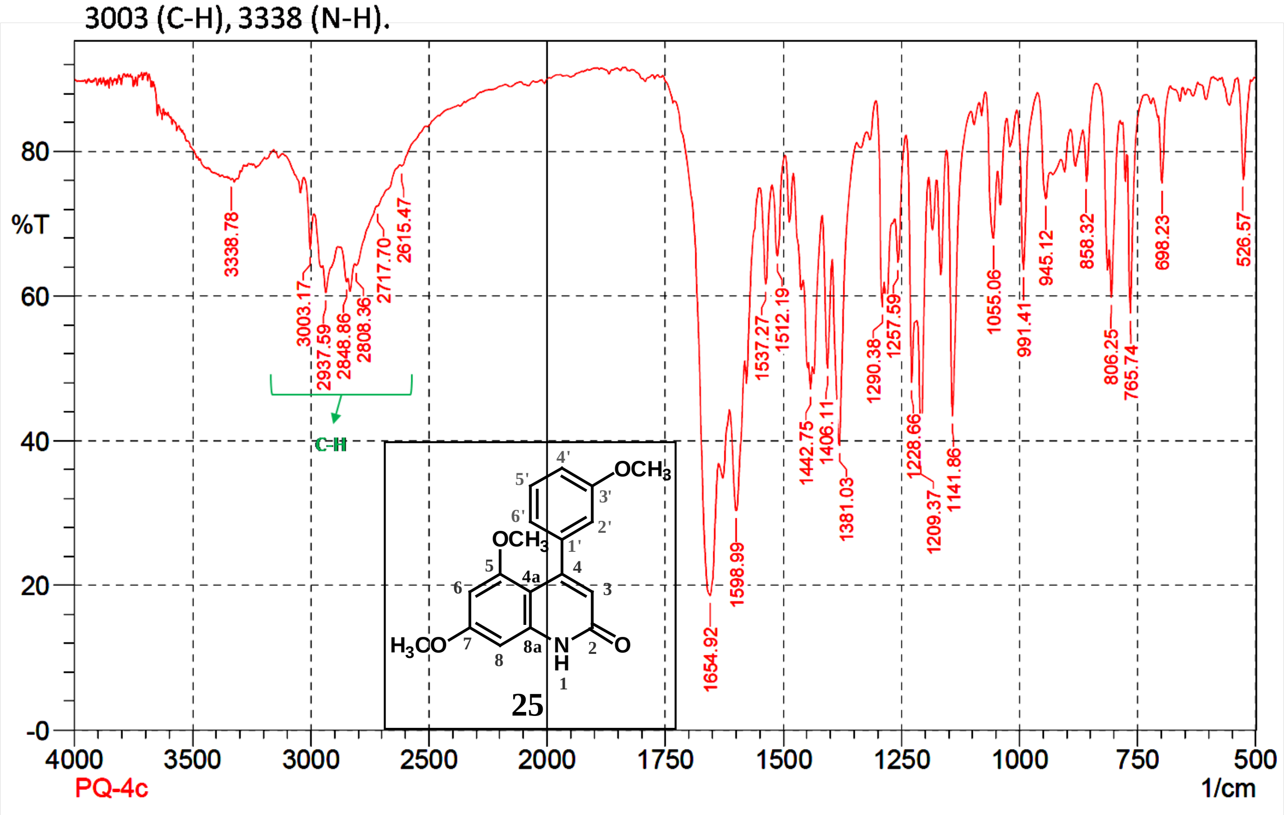


**Figure S51.** IR Spectra of compound **5,7-Dimethoxy-4-(3-methoxyphenyl)quinolin-2(1*H*)-one (25)**

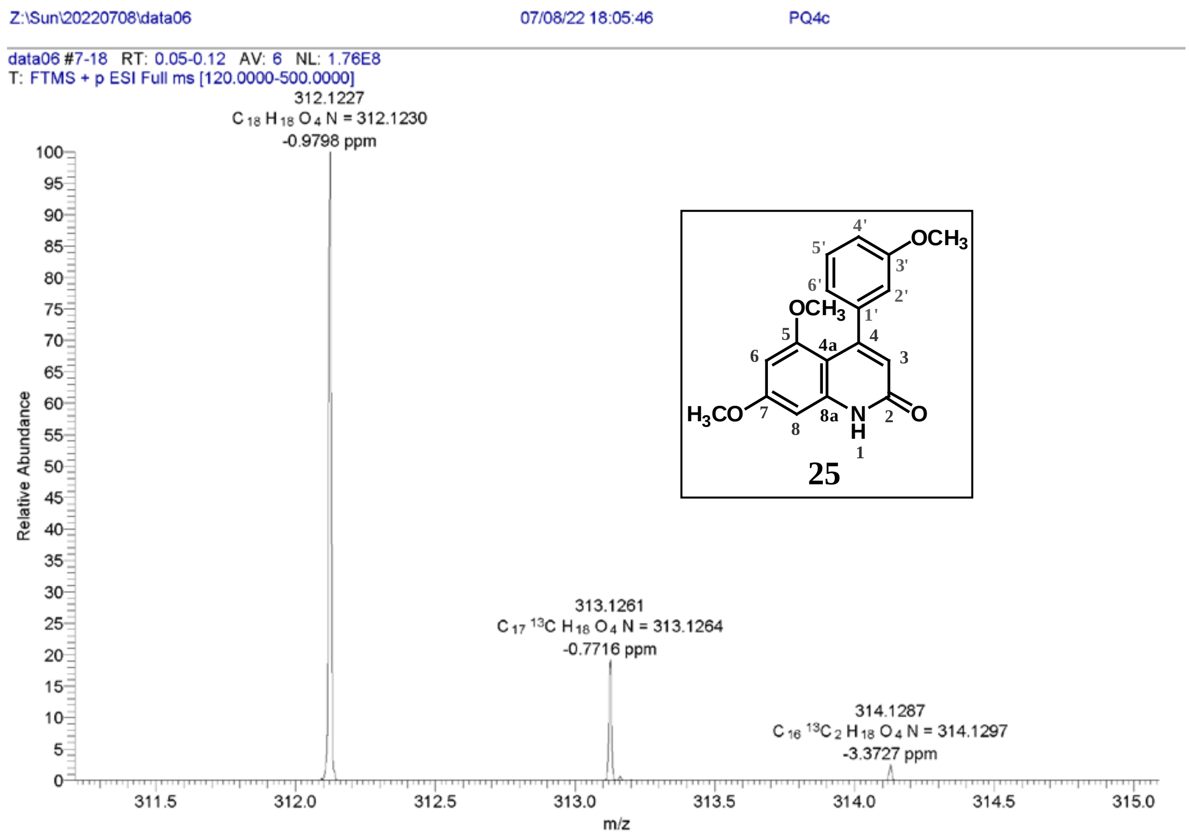


**Figure S52.** Mass Spectra of compound **5,7-Dimethoxy-4-(3-methoxyphenyl)quinolin-2(1*H*)-one (25)**

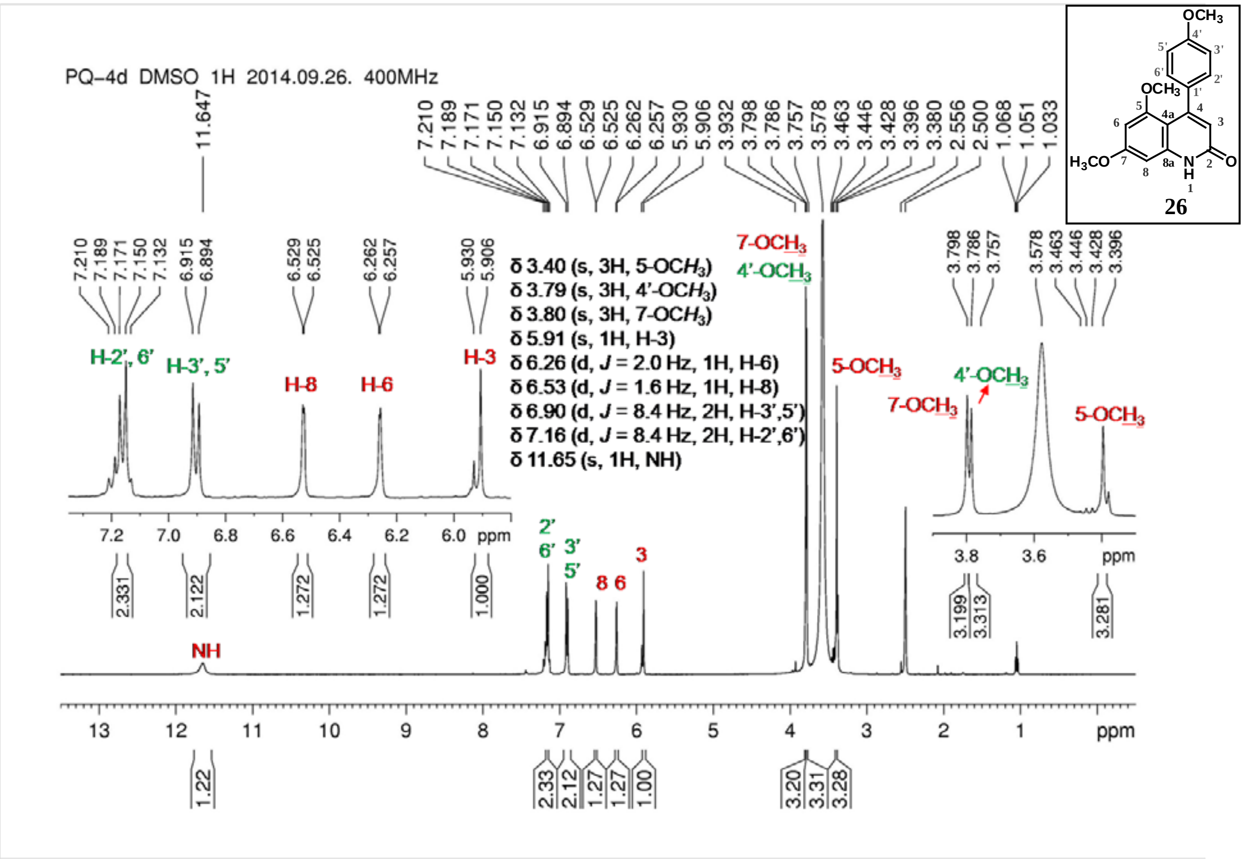


**Figure S53.** $^{1}$H-NMR Spectra of compound **5,7-Dimethoxy-4-(4-methoxyphenyl)quinolin-2(1*H*)-one (26)** was recorded in DMSO-d6 (400MHz)

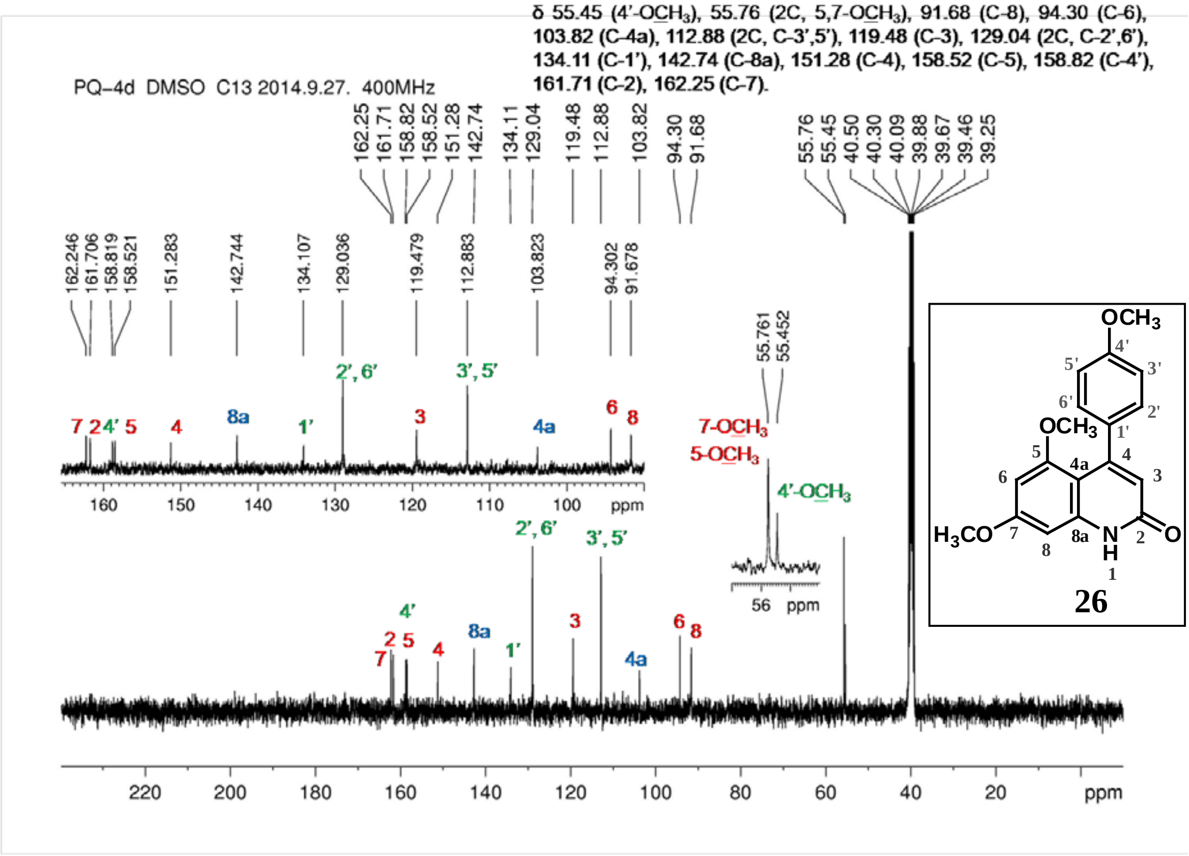


**Figure S54.**$^{13}$C-NMR Spectra of compound **5,7-Dimethoxy-4-(4-methoxyphenyl)quinolin-2(1*H*)-one (26)** was recorded in DMSO-*d6* (100MHz)

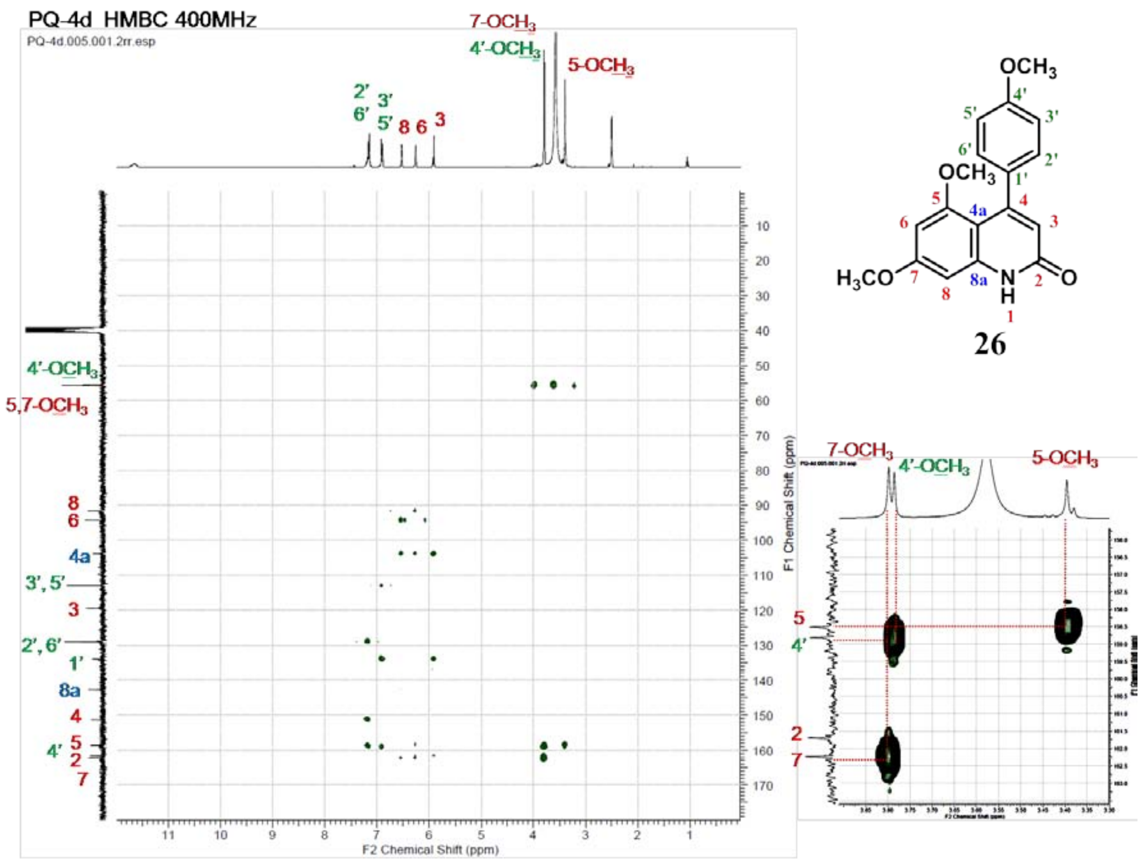


**Figure S55.** HMBC Spectra of compound **5,7-Dimethoxy-4-(4-methoxyphenyl)quinolin-2(1*H*)-one (26)** was recorded in DMSO-*d*₆ (400MHz)

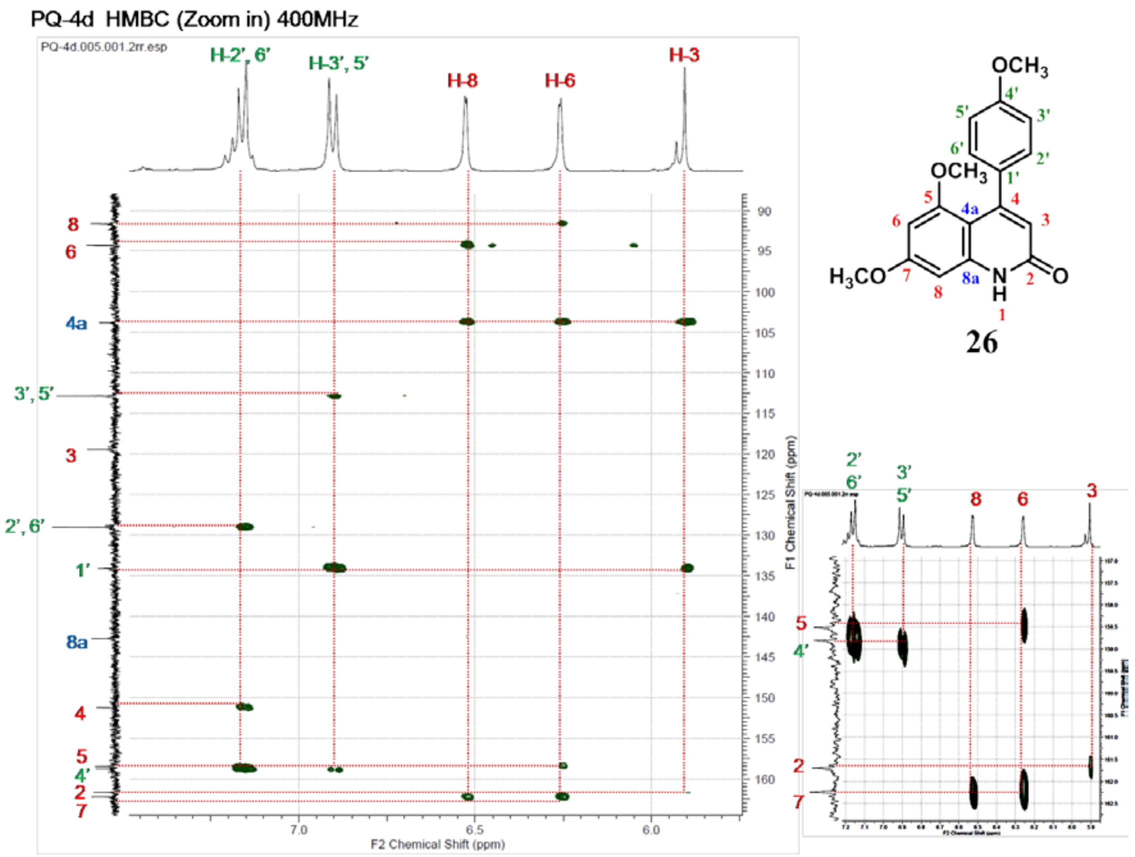


**Figure S56.** HMBC Spectra of compound **5,7-Dimethoxy-4-(4-methoxyphenyl)quinolin-2(1*H*)-one (26)** was recorded in DMSO-*d*₆ (400MHz Zoom in)

769, 813, 837, 983, 1033, 1060, 1139, 1168, 1184, 1209, 1244, 1379, 1435, 1519, 1600, 1631, 1660 (C=O), 2611-2945 (C-H), 3421 (N-H).

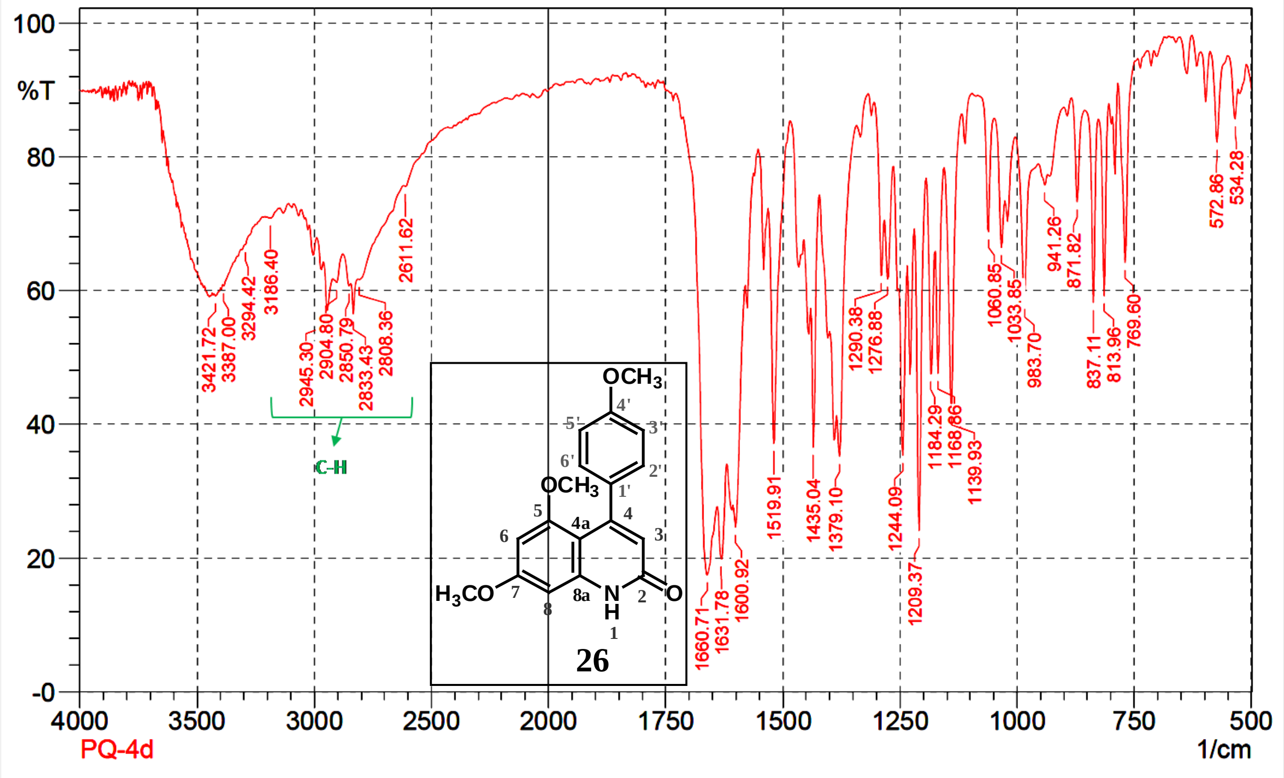


**Figure S57.** IR Spectra of compound **5,7-Dimethoxy-4-(4-methoxyphenyl)quinolin-2(1*H*)-one (26)**

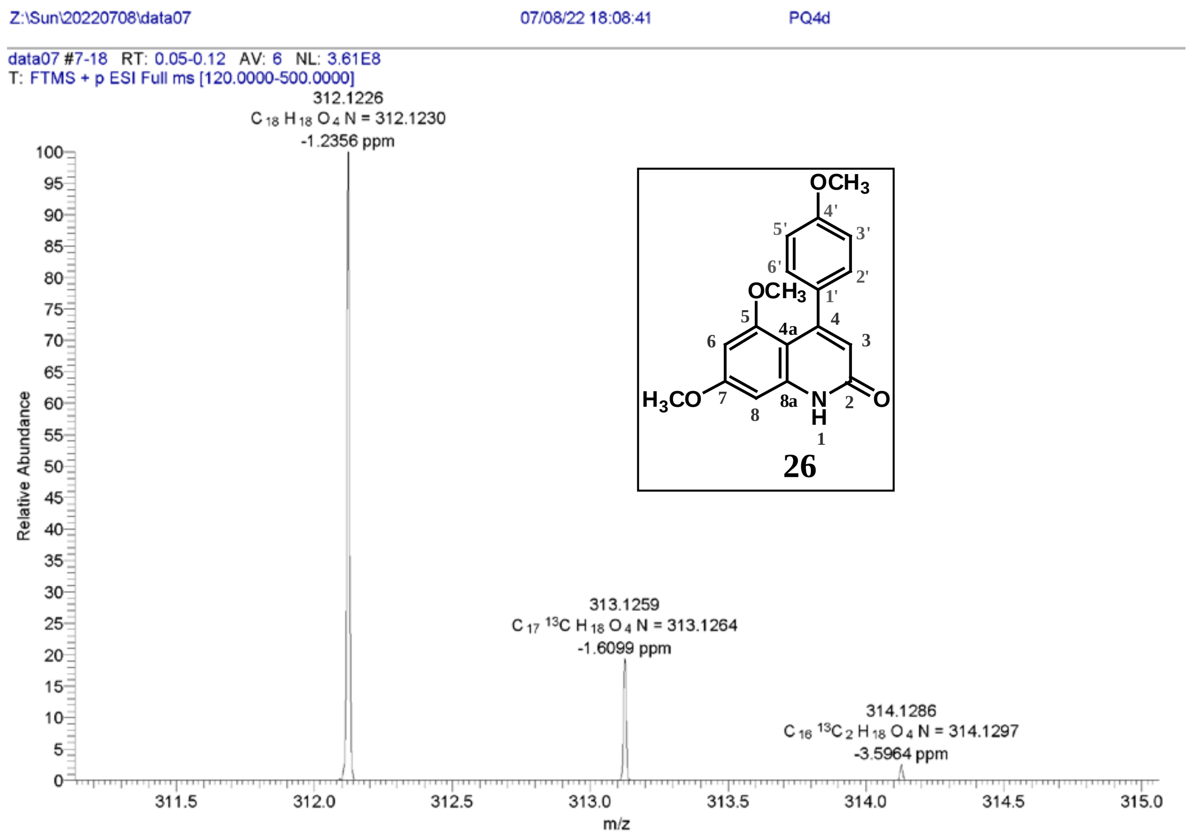


**Figure S58.** Mass Spectra of compound **5,7-Dimethoxy-4-(4-methoxyphenyl)quinolin-2(1*H*)-one (26)**

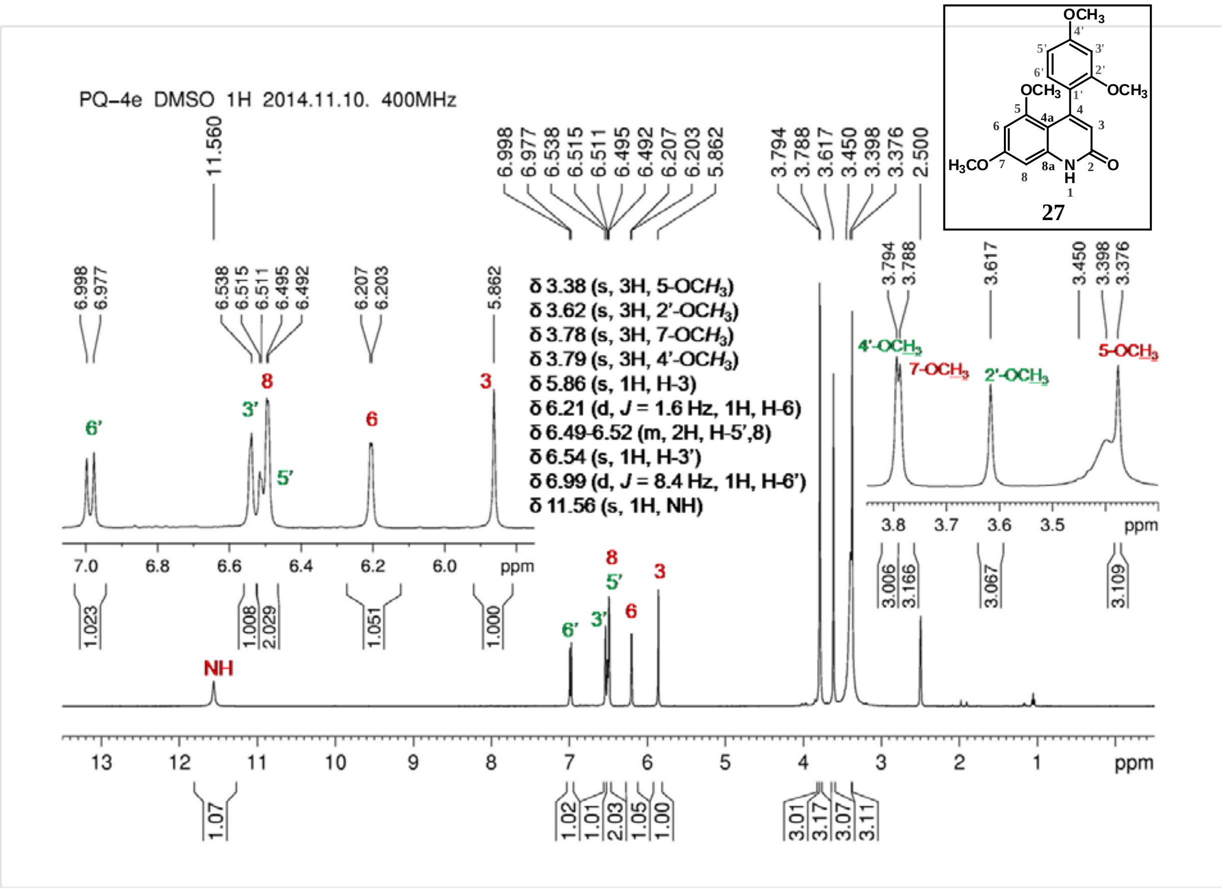


**Figure S59.** $^{1}$H-NMR Spectra of compound **4-(2,4-Dimethoxyphenyl)-5,7-dimethoxyquinolin-2(1*H*)-one (27)** was recorded in DMSO-d$_6$ (400MHz)

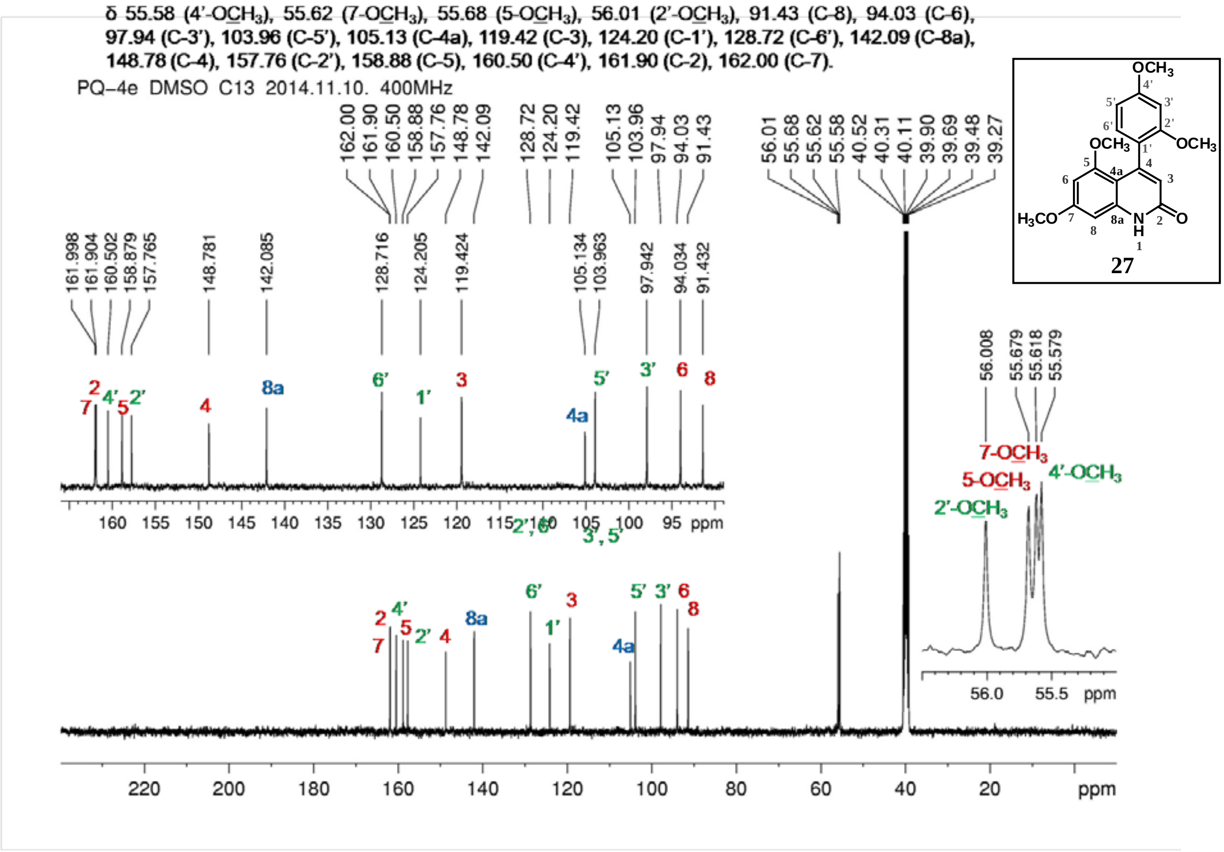


**Figure S60.** $^{13}$C-NMR Spectra of compound **4-(2,4-Dimethoxyphenyl)-5,7-dimethoxyquinolin-2(1*H*)-one (27)** was recorded in DMSO-*d*$_6$ (100MHz)

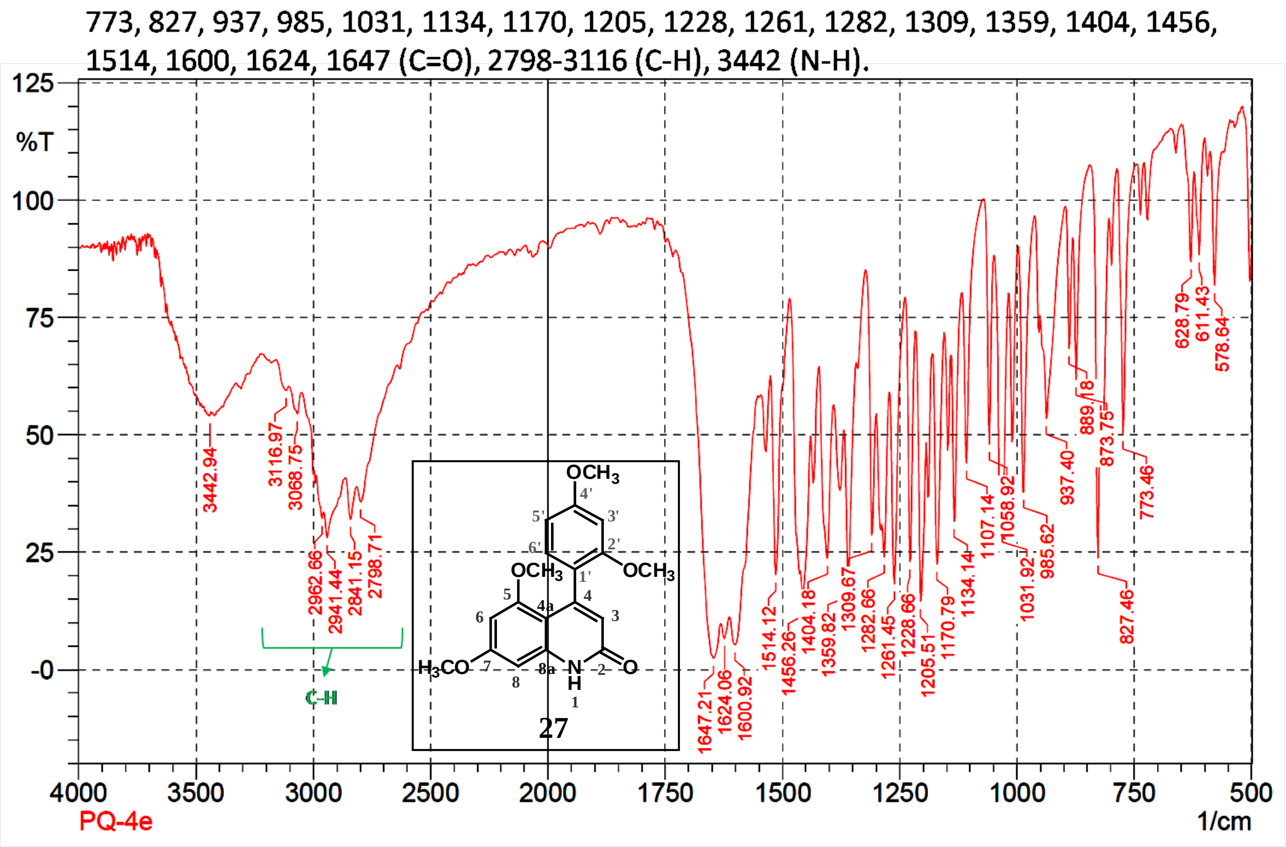


**Figure S61.** IR Spectra of compound **4-(2,4-Dimethoxyphenyl)-5,7-dimethoxyquinolin-2(1*H*)-one (27)**

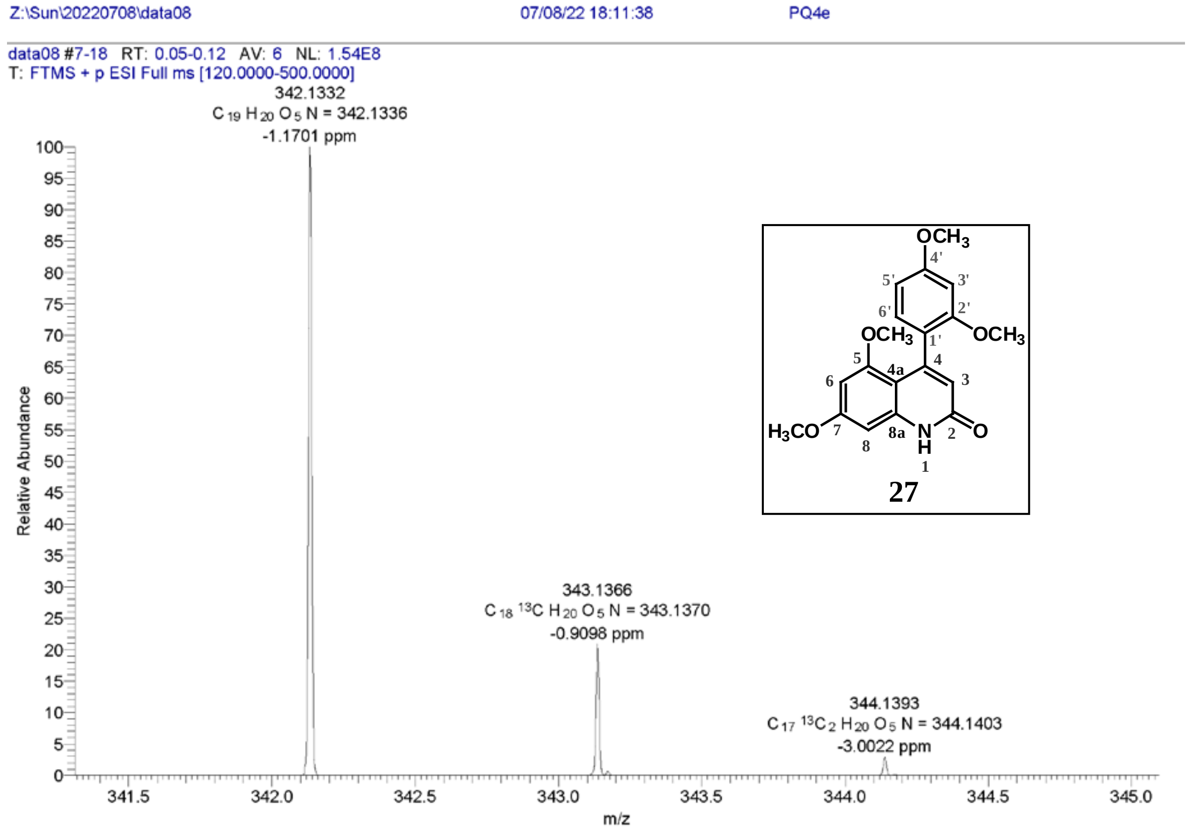


**Figure S62.** Mass Spectra of compound **4-(2,4-Dimethoxyphenyl)-5,7-dimethoxyquinolin-2(1*H*)-one (27)**

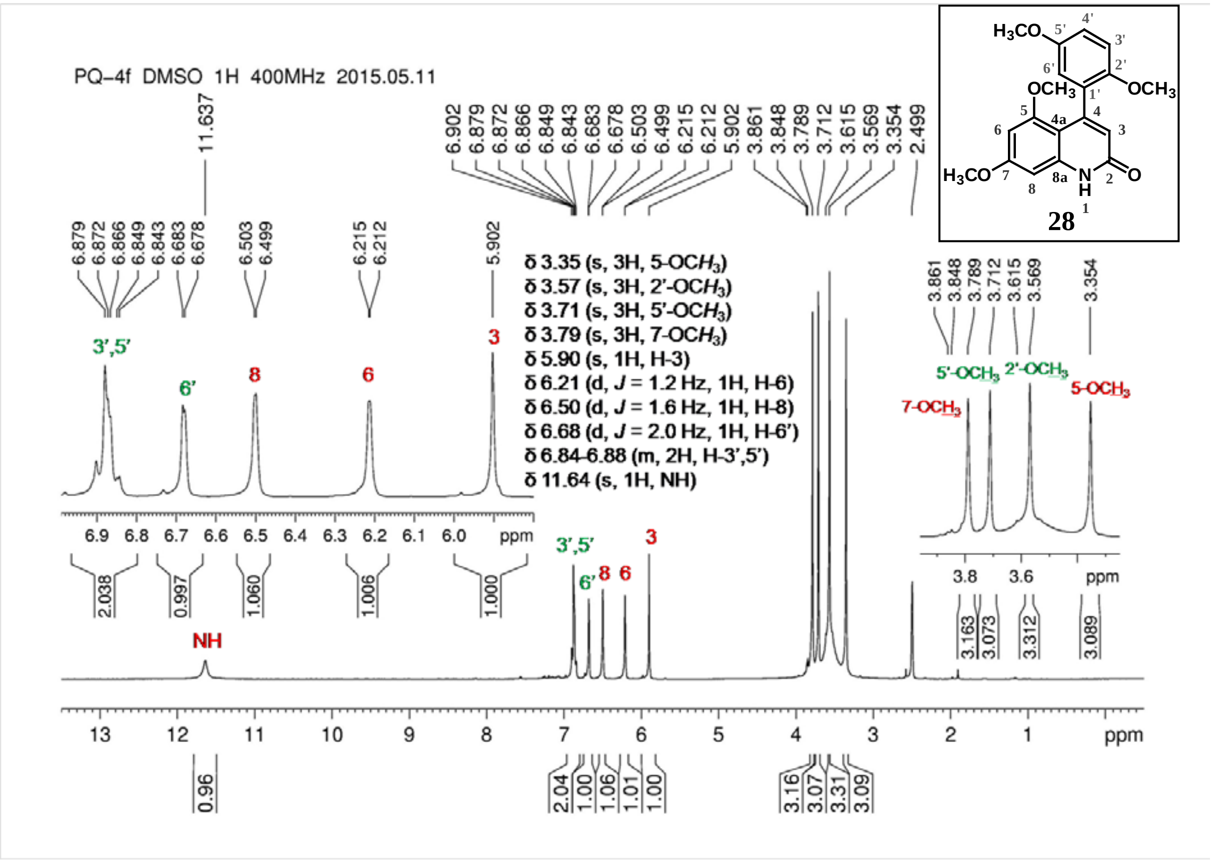


**Figure S63.** ¹H-NMR Spectra of compound **4-(2,5-Dimethoxyphenyl)-5,7-dimethoxyquinolin-2(1*H*)-one (28)** was recorded in DMSO-d6 (400MHz)

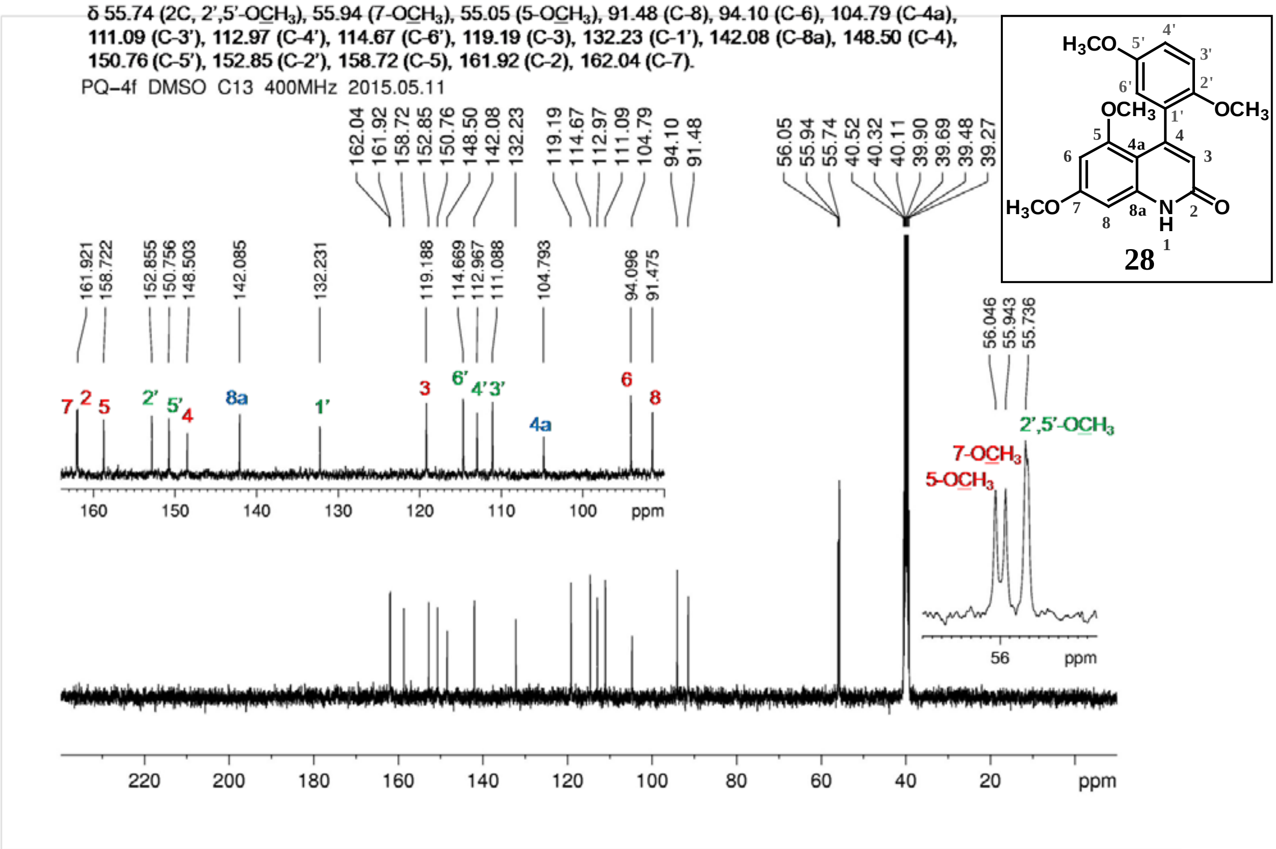


**Figure S64.** ¹³C-NMR Spectra of compound **4-(2,5-Dimethoxyphenyl)-5,7-dimethoxyquinolin-2(1*H*)-one (28)** was recorded in DMSO-*d6* (100MHz)

773, 798, 821, 948, 991, 1047, 1143, 1168, 1207, 1228, 1273, 1375, 1404, 1452, 1496, 1514, 1606, 1624, 1664 (C=O), 2607-2995 (C-H).

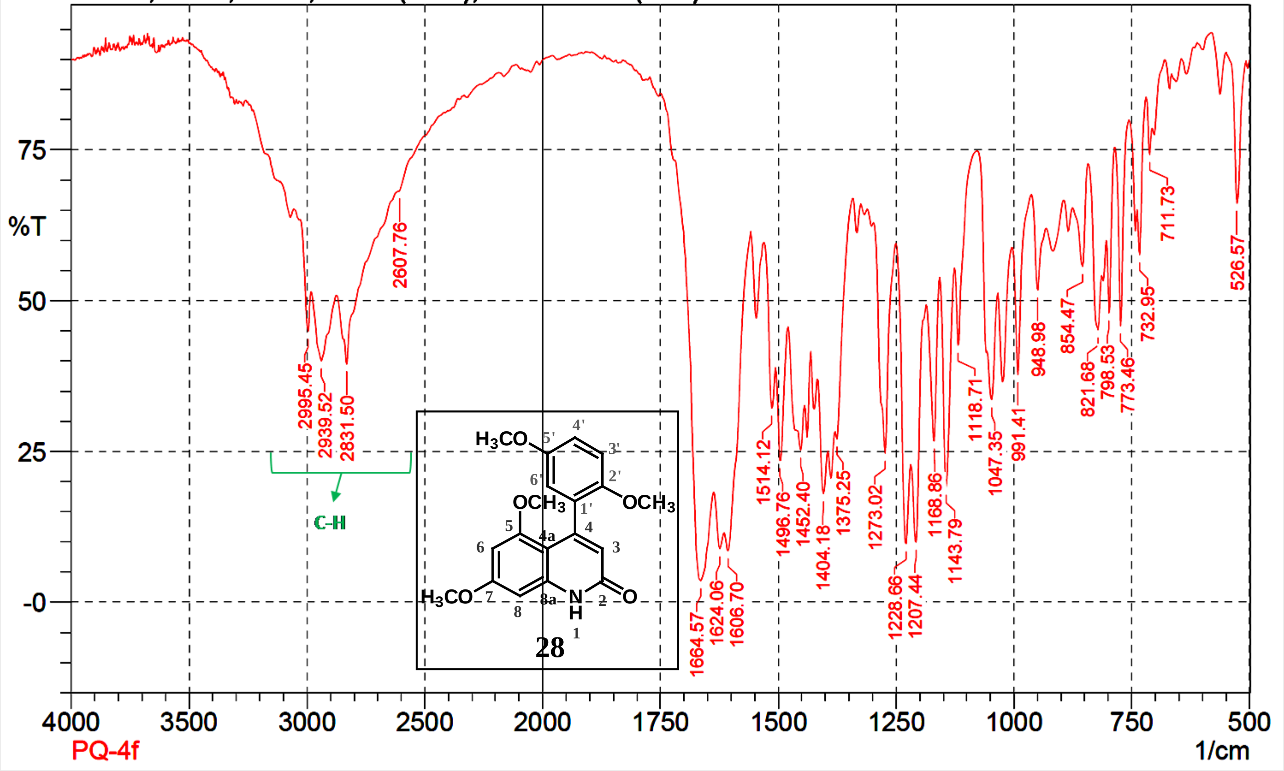


**Figure S65.** IR Spectra of compound **4-(2,5-Dimethoxyphenyl)-5,7-dimethoxyquinolin-2(1*H*)-one (28)**

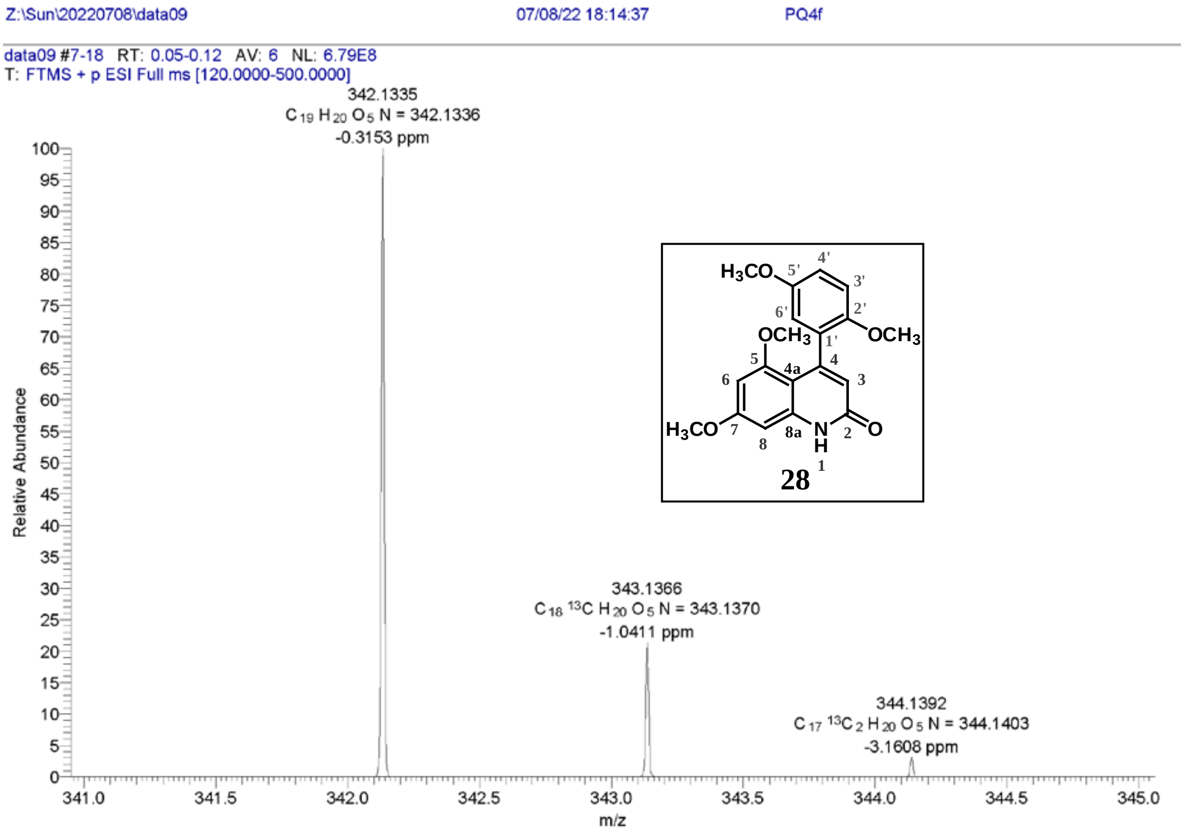


**Figure S66.** IR Spectra of compound **4-(2,5-Dimethoxyphenyl)-5,7-dimethoxyquinolin-2(1*H*)-one (28)**

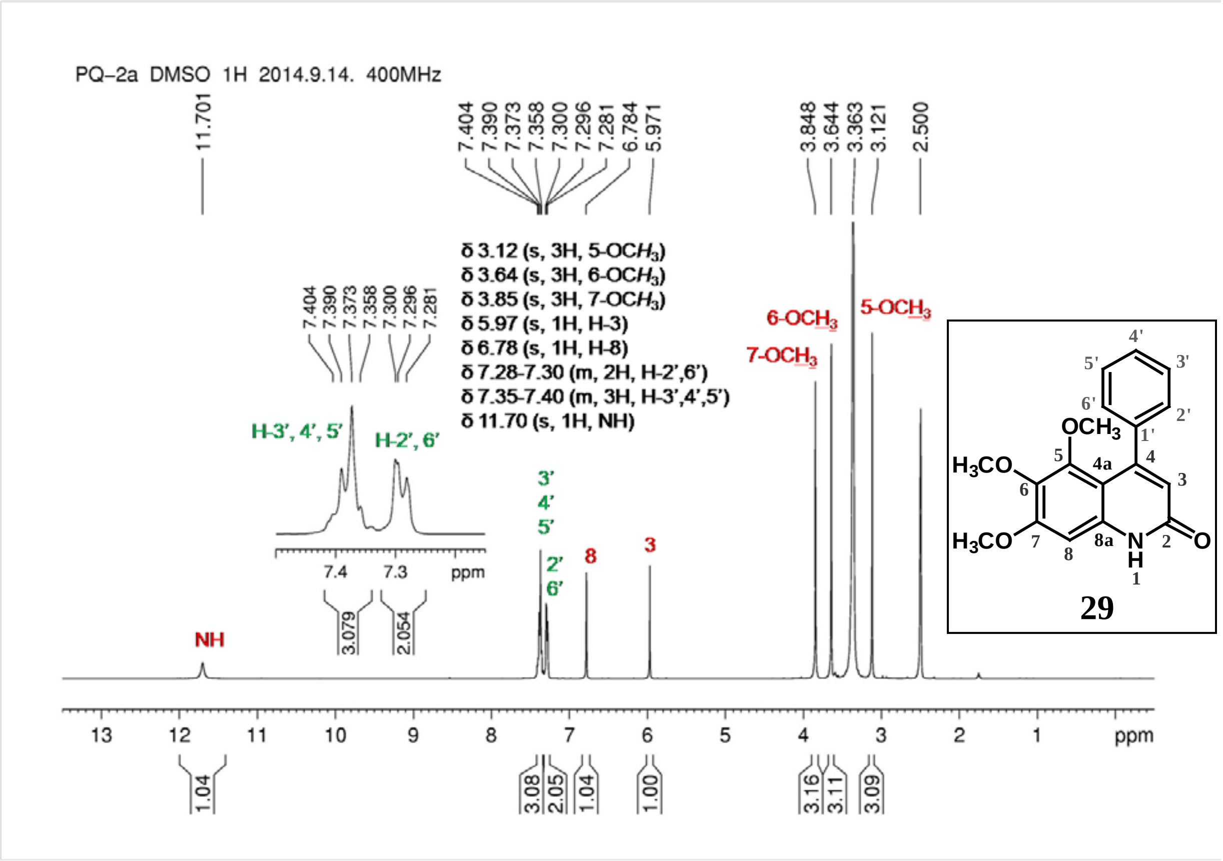


**Figure S67.** $^{1}$H-NMR Spectra of compound **5,6,7-Trimethoxy-4-phenylquinolin-2(1*H*)-one (29)** was recorded in DMSO-d6 (400MHz)

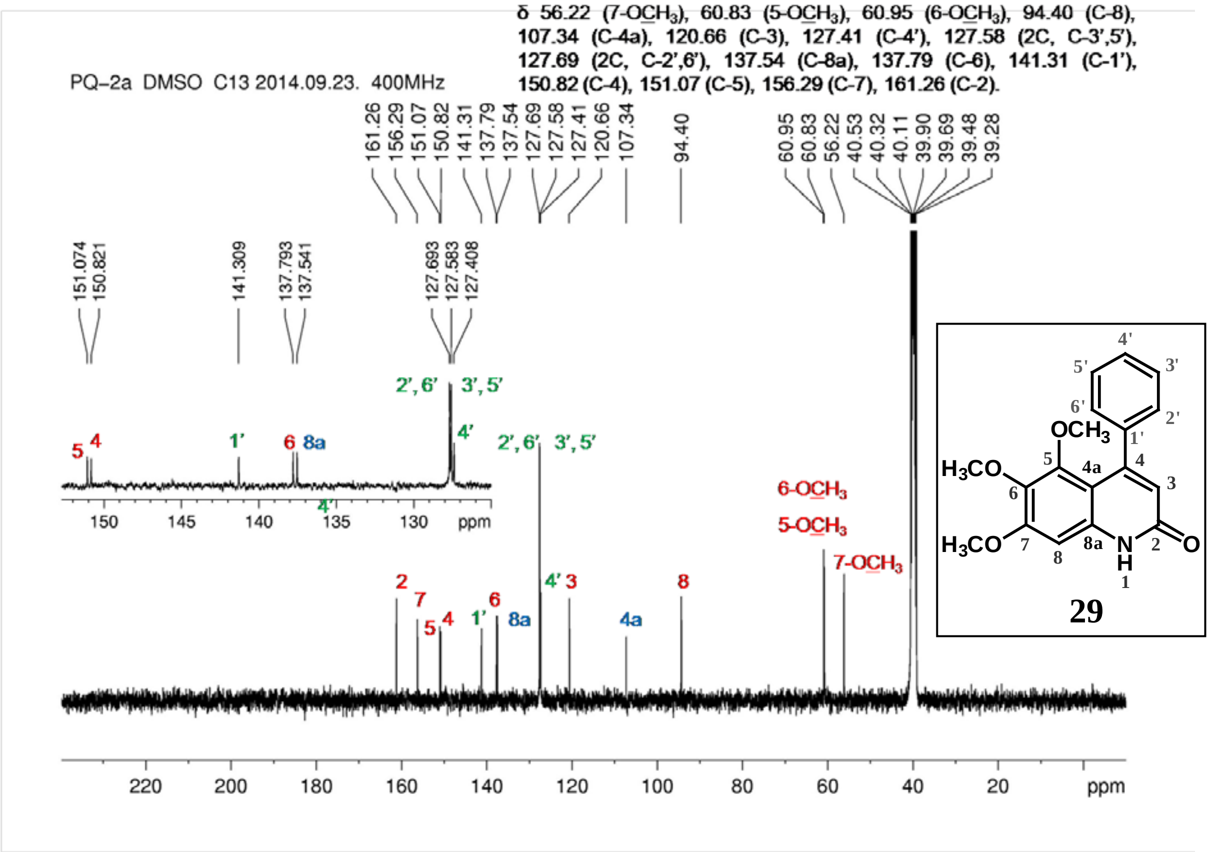


**Figure S68.** $^{13}$C-NMR Spectra of compound **5,6,7-Trimethoxy-4-phenylquinolin-2(1*H*)-one (29)** was recorded in DMSO-*d6* (100MHz)

750, 869, 943, 1047, 1118, 1195, 1213, 1240, 1271, 1359, 1386, 1409, 1442, 1471, 1490, 1614, 1654 (C=O), 2601-3138 (C-H), 3431 (N-H).

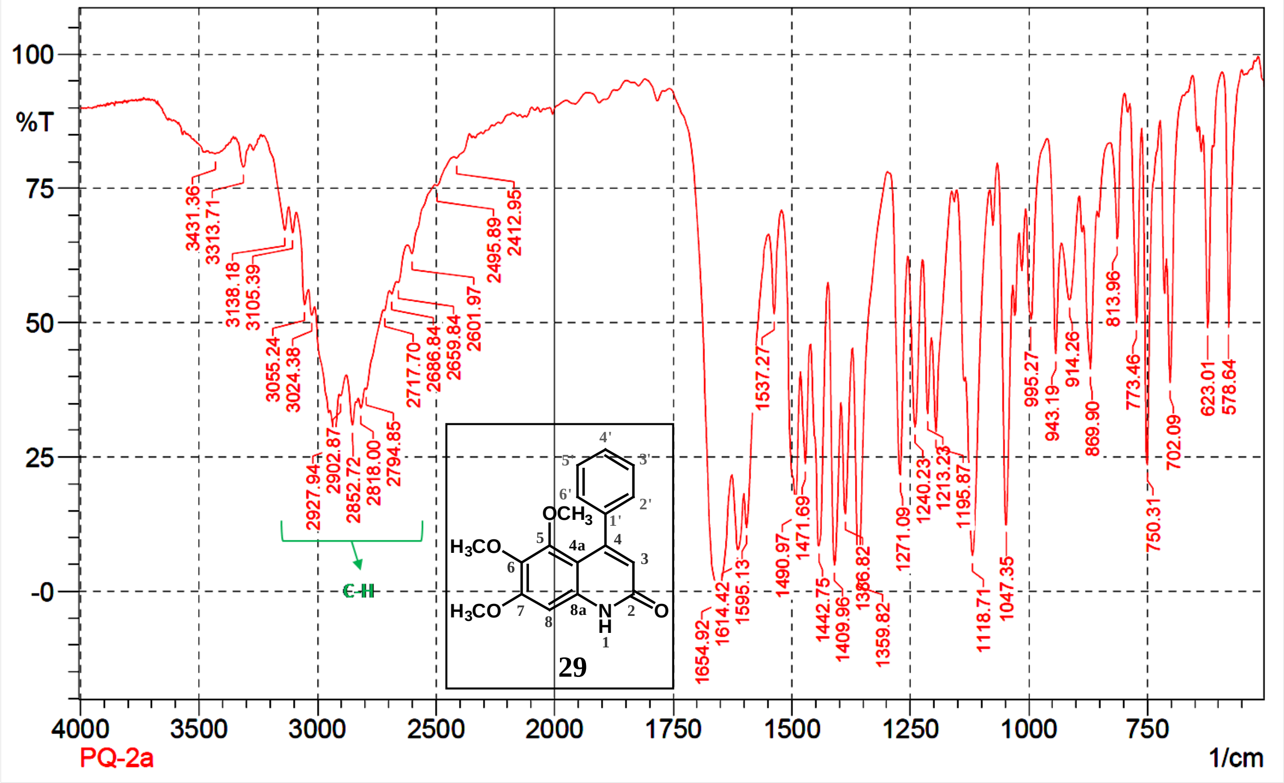


**Figure S69.** IR Spectra of compound **5,6,7-Trimethoxy-4-phenylquinolin-2(1*H*)-one (29)**

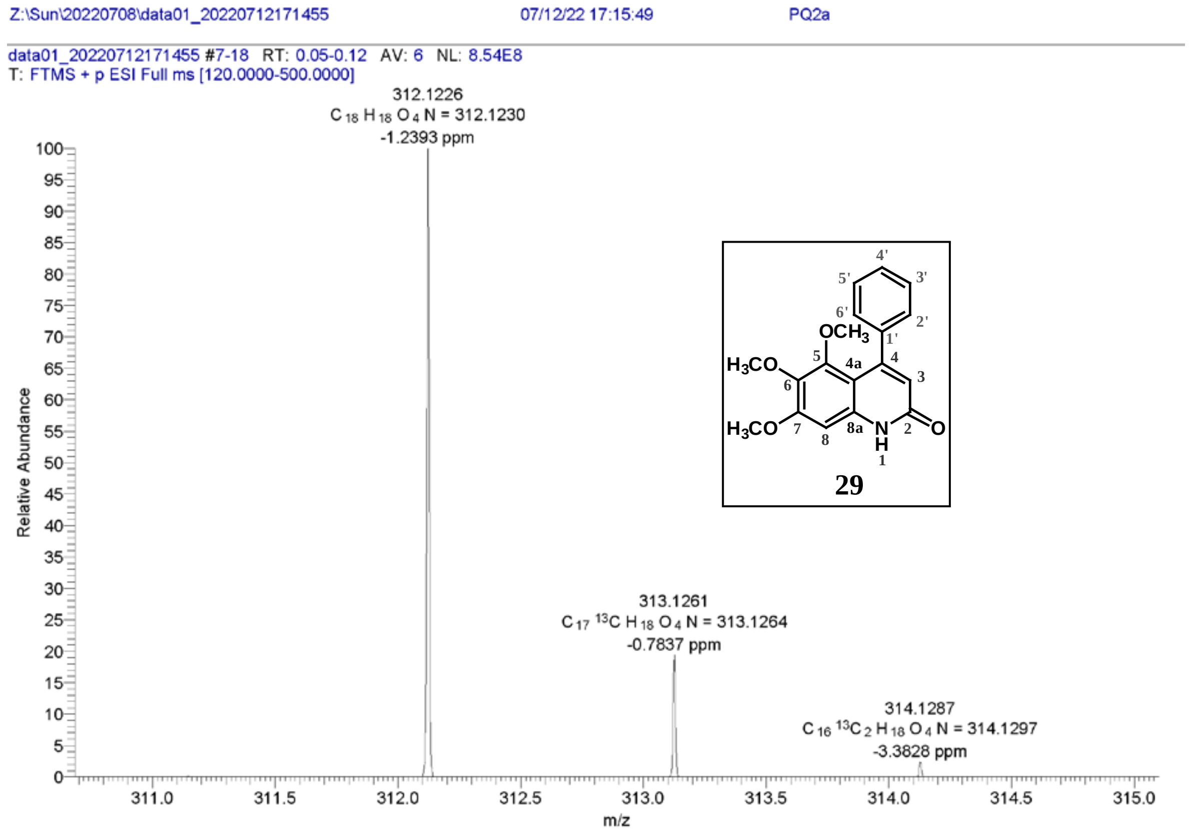


**Figure S70.** IR Spectra of compound **5,6,7-Trimethoxy-4-phenylquinolin-2(1*H*)-one (29)**

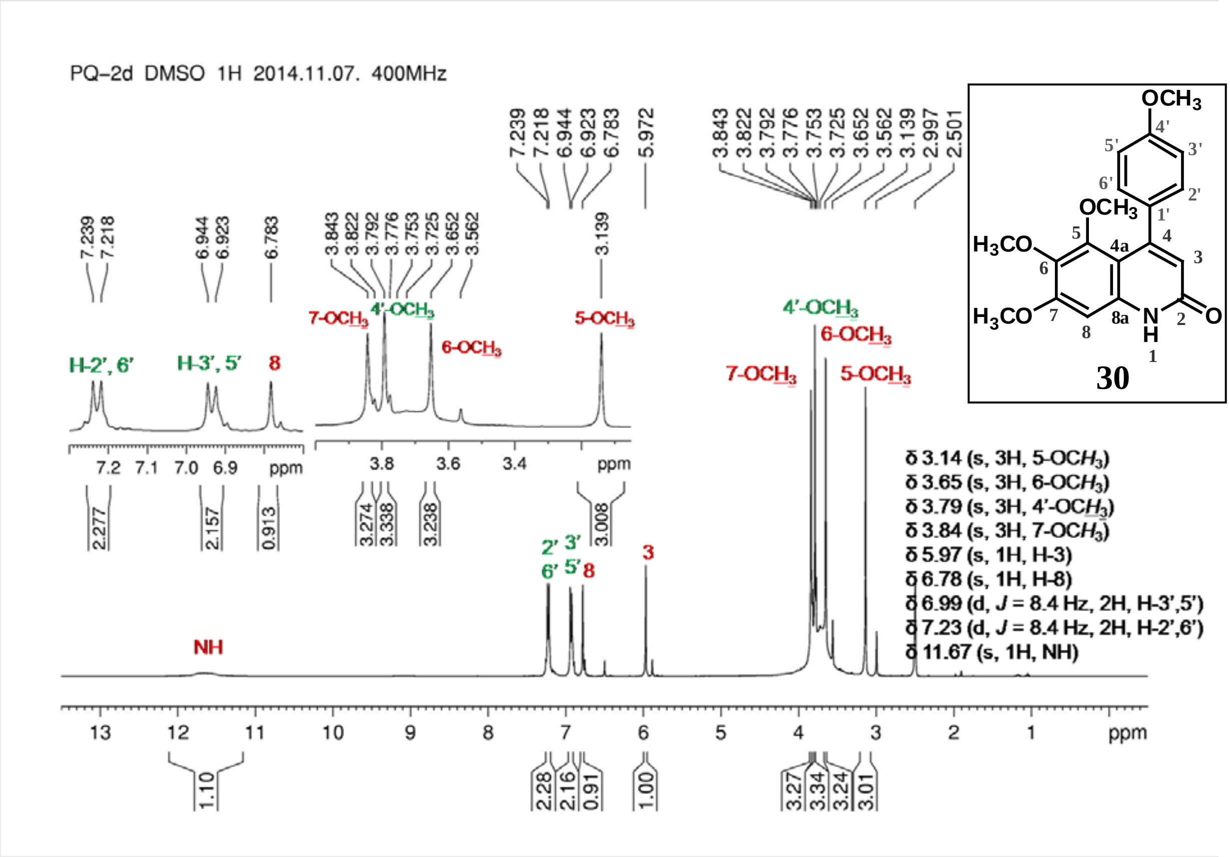


**Figure S71.** $^{1}$H-NMR Spectra of compound **5,6,7-Trimethoxy-4-(4-methoxyphenyl)quinolin-2(1*H*)-one (30)** was recorded in DMSO-d6 (400MHz)

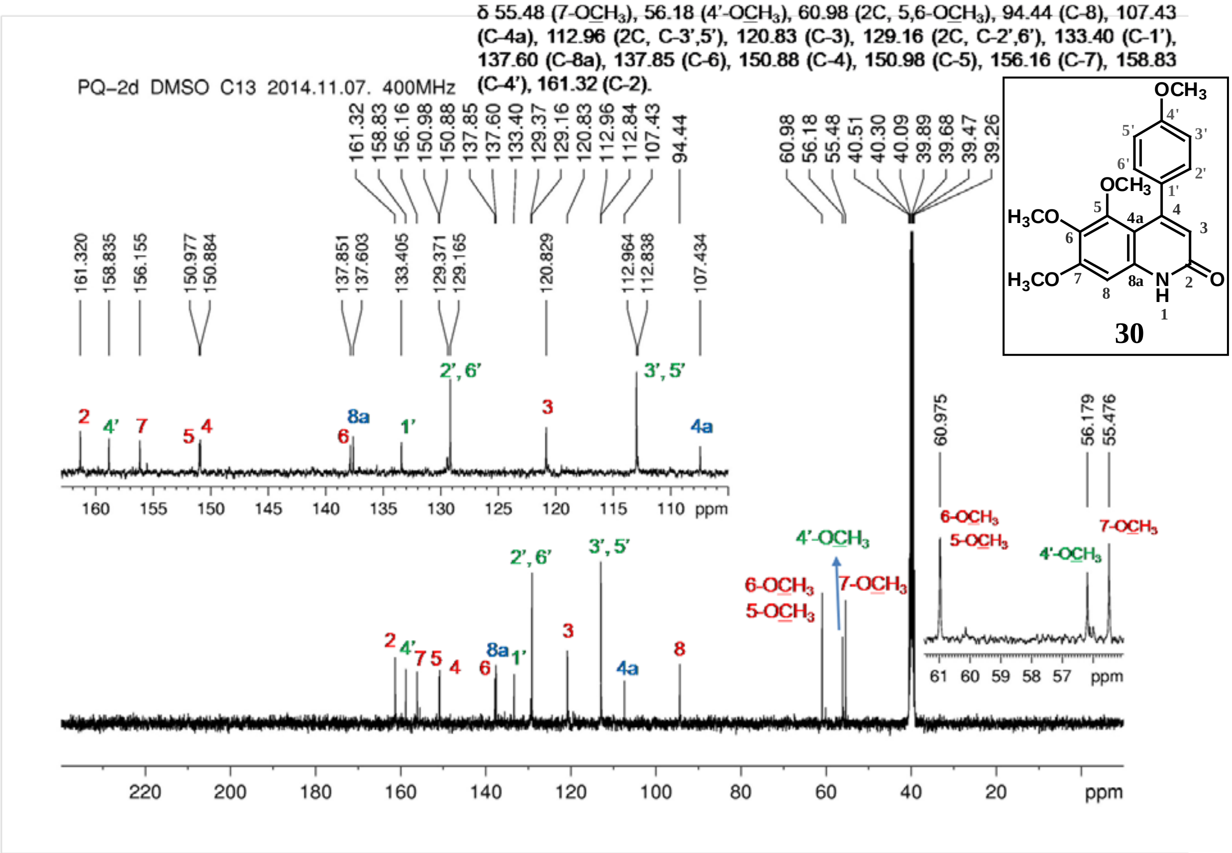


**Figure S72.** $^{13}$C-NMR Spectra of compound **5,6,7-Trimethoxy-4-(4-methoxyphenyl)quinolin-2(1*H*)-one (30)** was recorded in DMSO-*d6* (100MHz)

750, 833, 1047, 1116, 1176, 1244, 1359, 1408, 1442, 1494, 1510, 1612, 1654 (C=O), 2603-3136 (C-H), 3387 (N-H).

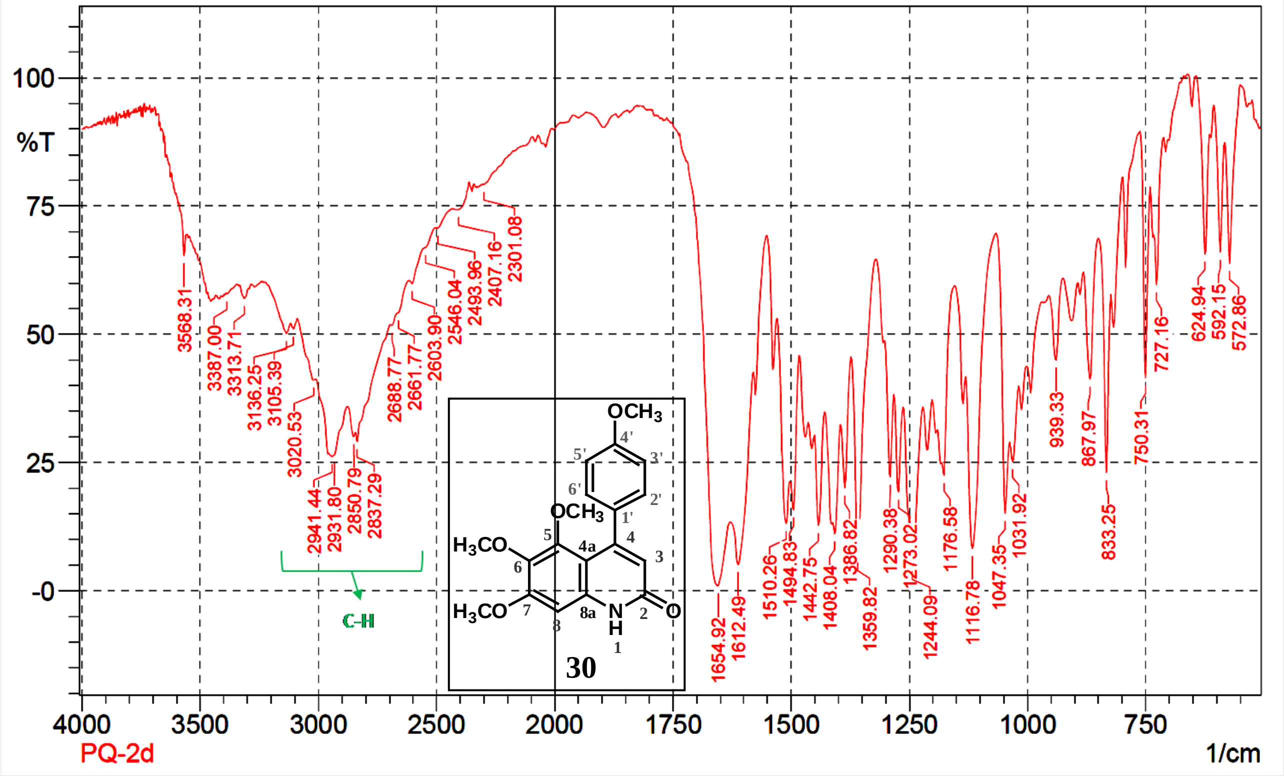


**Figure S73.** IR Spectra of compound **5,6,7-Trimethoxy-4-(4-methoxyphenyl)quinolin-2(1*H*)-one (30)**

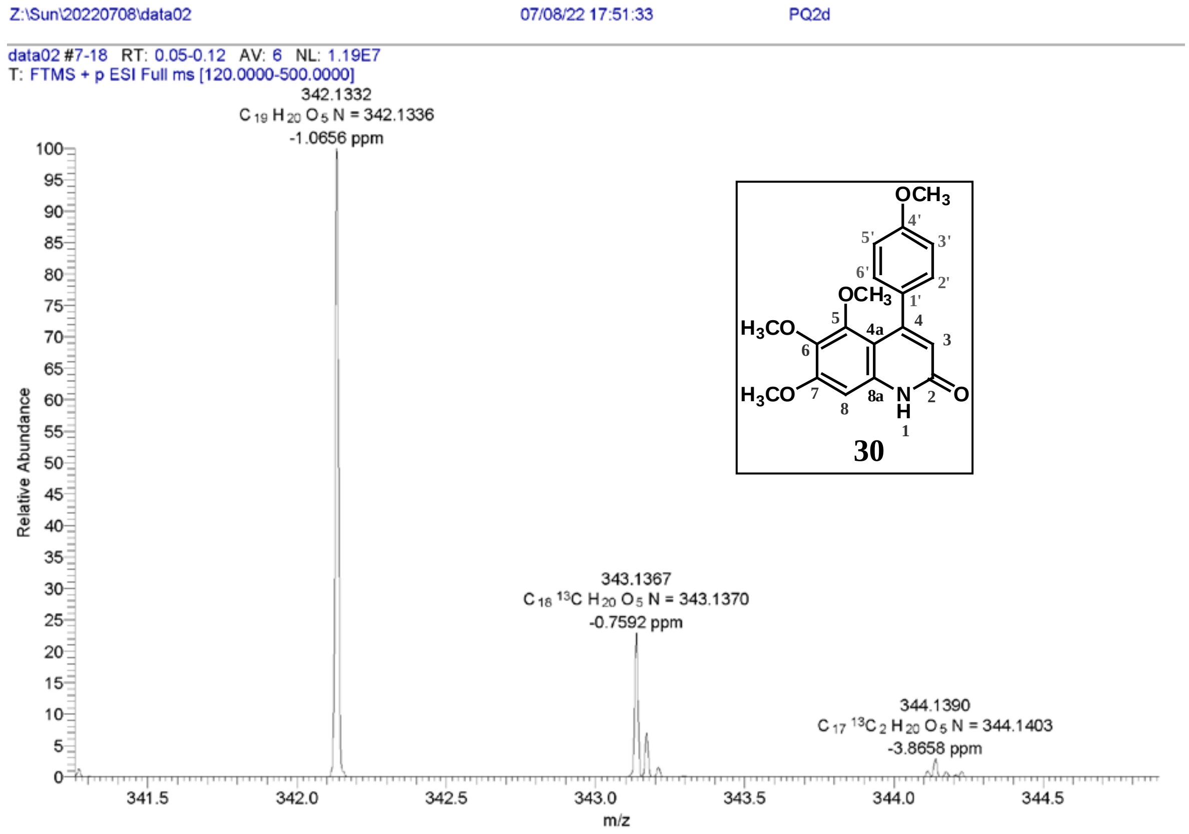


**Figure S74.** IR Spectra of compound **5,6,7-Trimethoxy-4-(4-methoxyphenyl)quinolin-2(1*H*)-one (30)**

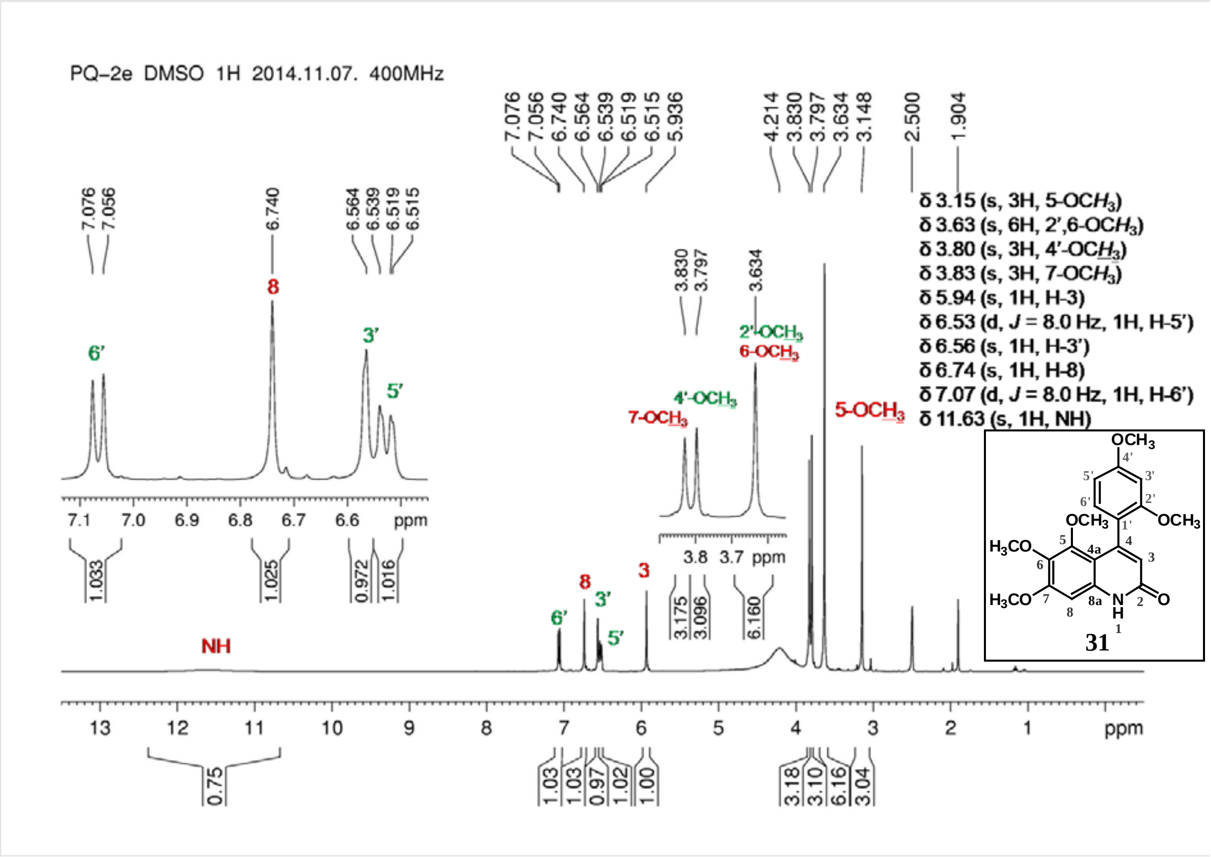


**Figure S75.** $^{1}$H-NMR Spectra of compound **4-(2,4-Dimethoxyphenyl)-5,6,7-trimethoxyquinolin-2(1*H*)-one (31)** was recorded in DMSO-d6 (400MHz)

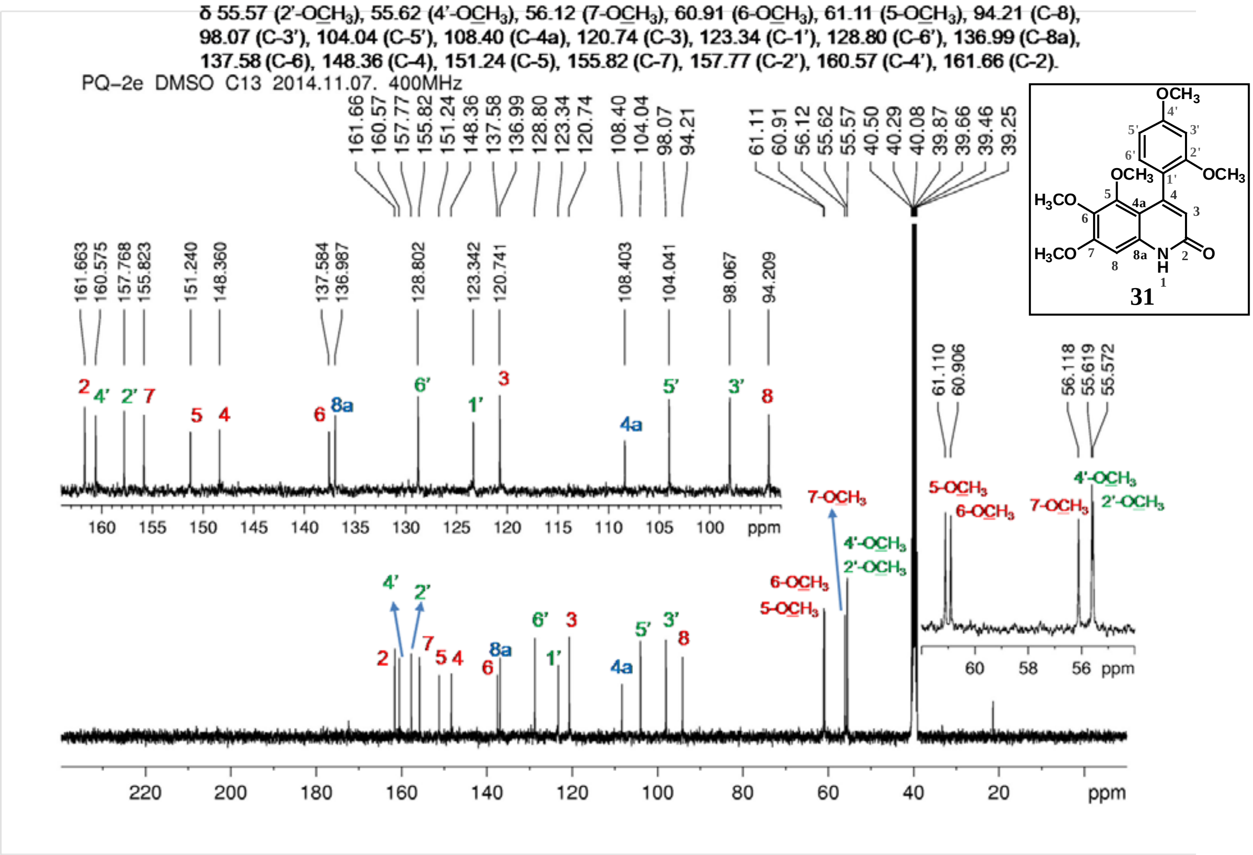


**Figure S76.** $^{13}$C-NMR Spectra of compound **4-(2,4-Dimethoxyphenyl)-5,6,7-trimethoxyquinolin-2(1*H*)-one (31)** was recorded in DMSO-*d6* (100MHz)

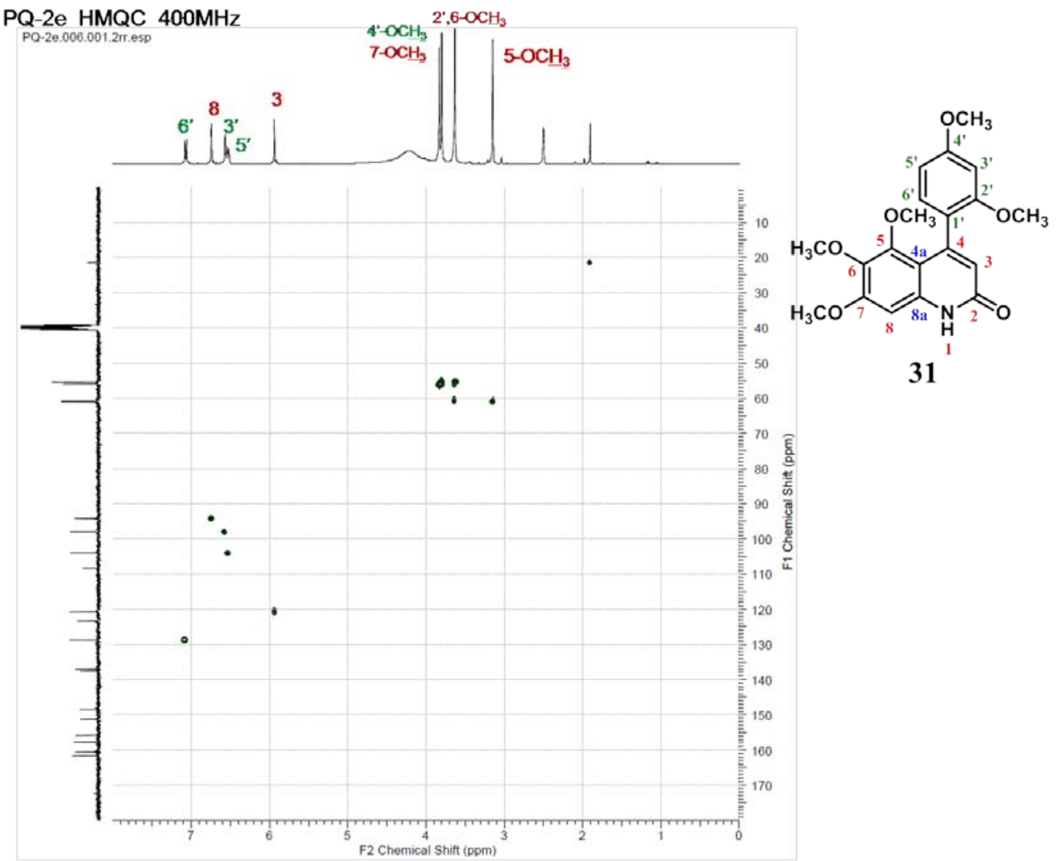


**Figure S77.** HMQC Spectra of compound **4-(2,4-Dimethoxyphenyl)-5,6,7-trimethoxyquinolin-2(1*H*)-one (31)** was recorded in DMSO-$d_6$ (400MHz)

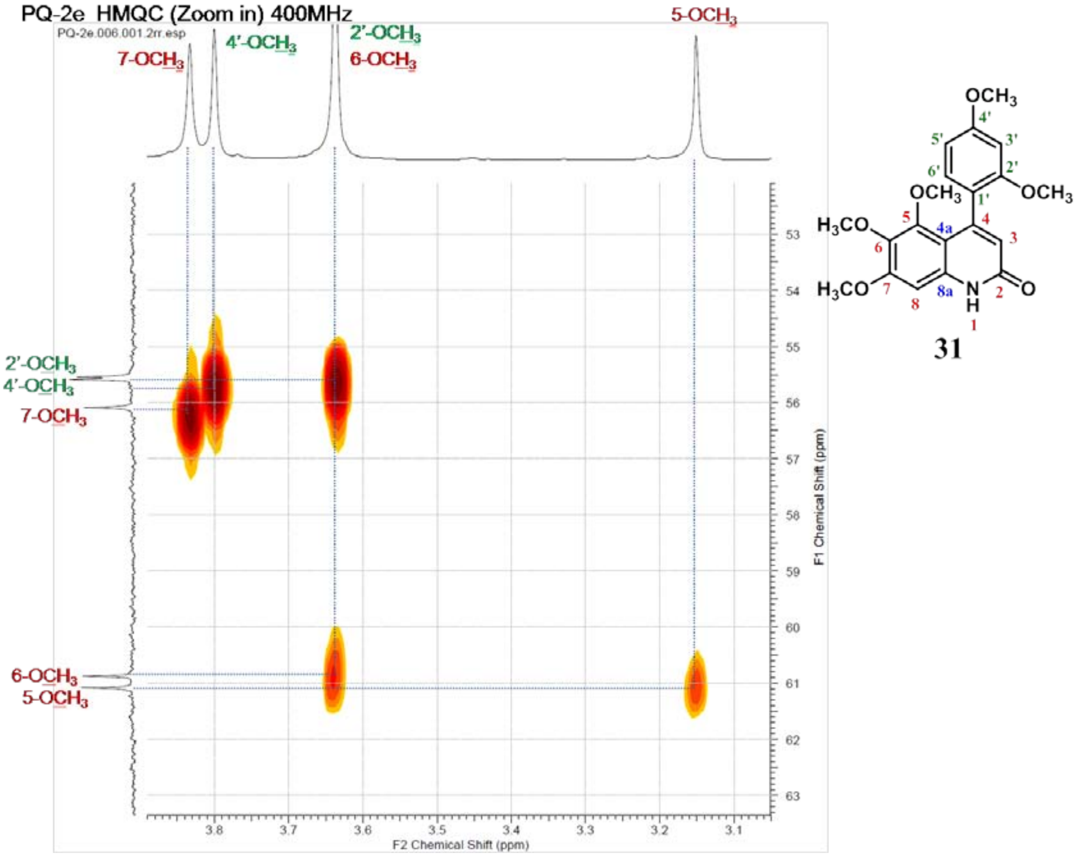


**Figure S78.** HMQC Spectra of compound **4-(2,4-Dimethoxyphenyl)-5,6,7-trimethoxyquinolin-2(1*H*)-one (31)** was recorded in DMSO-$d_6$ (400MHz Zoom in)

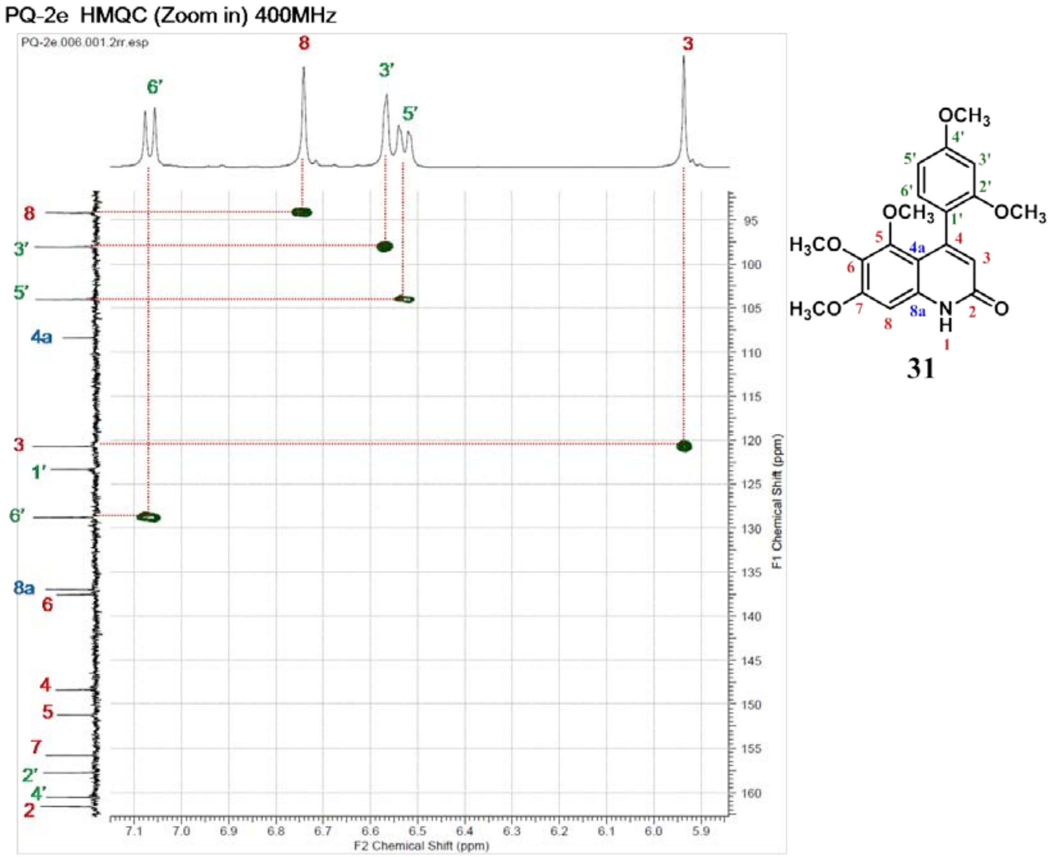


**Figure S79.** HMQC Spectra of compound **4-(2,4-Dimethoxyphenyl)-5,6,7-trimethoxyquinolin-2(1*H*)-one (31)** was recorded in DMSO-$d_6$ (400MHz Zoom in)

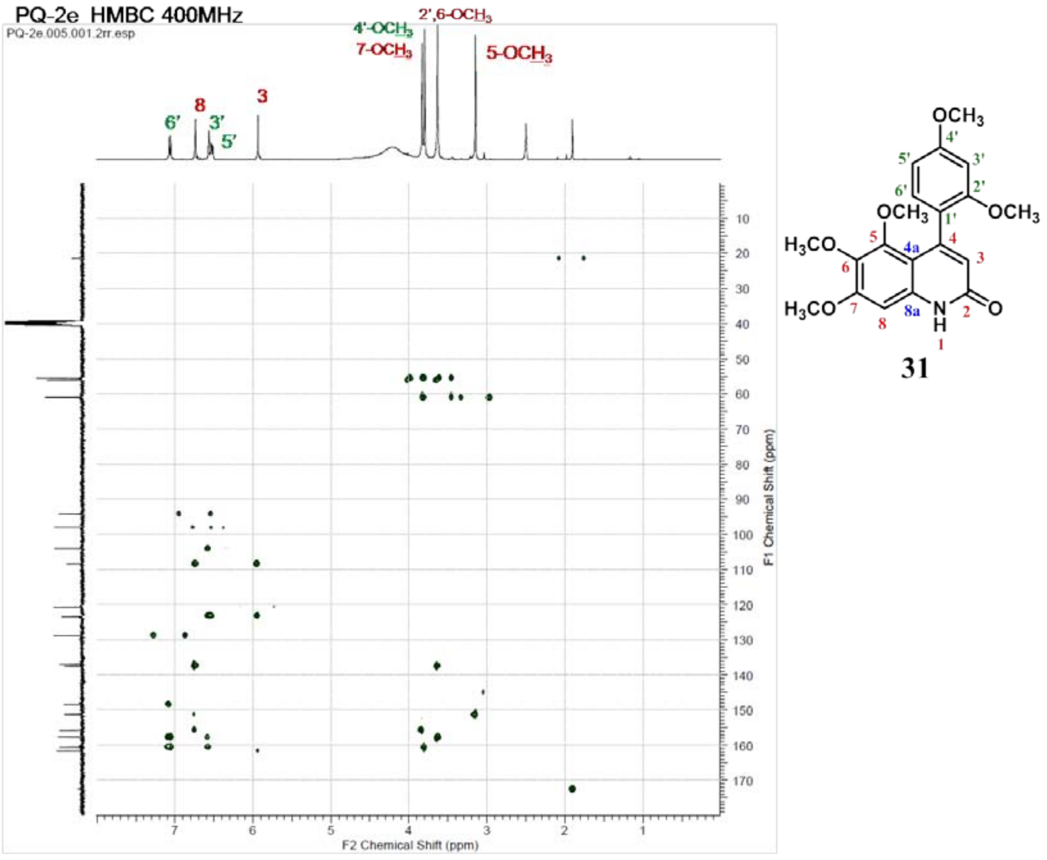


**Figure S80.** HMBC Spectra of compound **4-(2,4-Dimethoxyphenyl)-5,6,7-trimethoxyquinolin-2(1*H*)-one (31)** was recorded in DMSO-$d_6$ (400MHz)

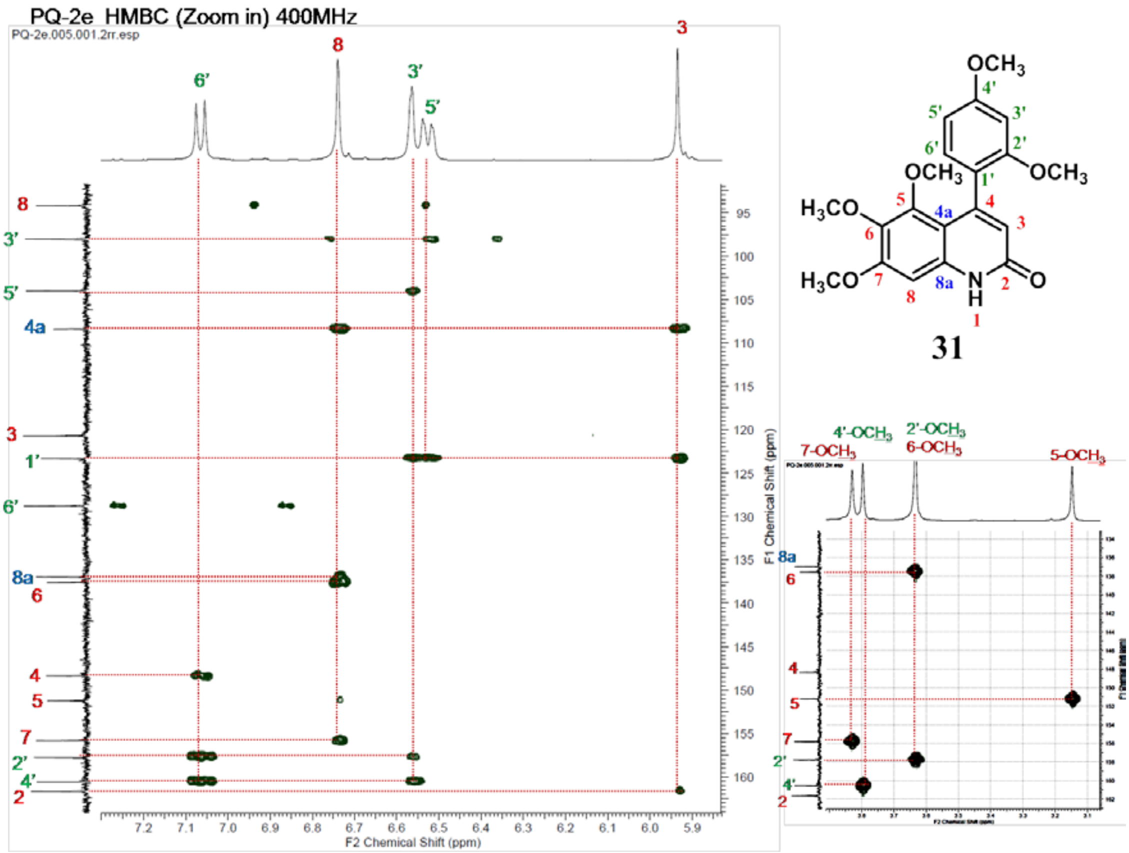


**Figure S81.** HMBC Spectra of compound **4-(2,4-Dimethoxyphenyl)-5,6,7-trimethoxyquinolin-2(1*H*)-one (31)** was recorded in DMSO-$d_6$ (400MHz Zoom in)

831, 866, 943, 1039, 1114, 1159, 1211, 1273, 1357, 1406, 1436, 1469, 1504, 1612, 1651 (C=O), 2611-3136 (C-H), 3394 (N-H).

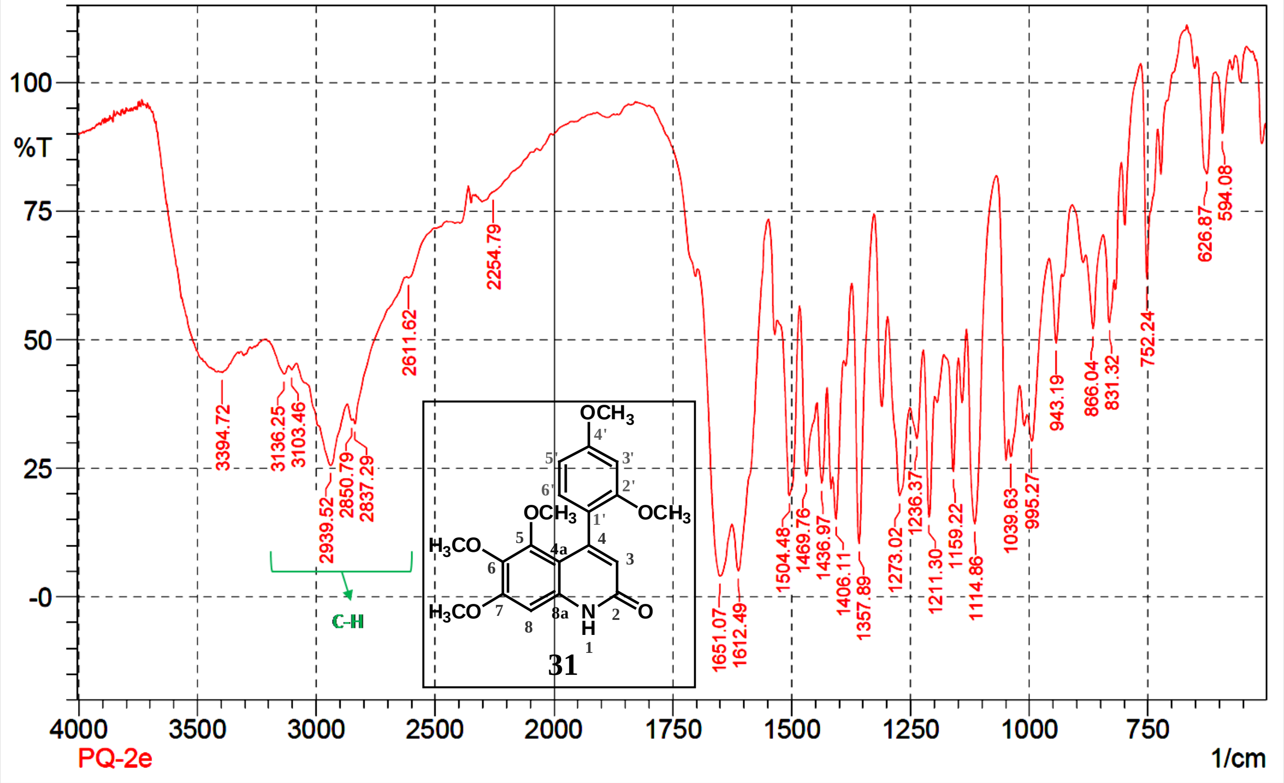


**Figure S82.** IR Spectra of compound **4-(2,4-Dimethoxyphenyl)-5,6,7-trimethoxyquinolin-2(1*H*)-one (31)**

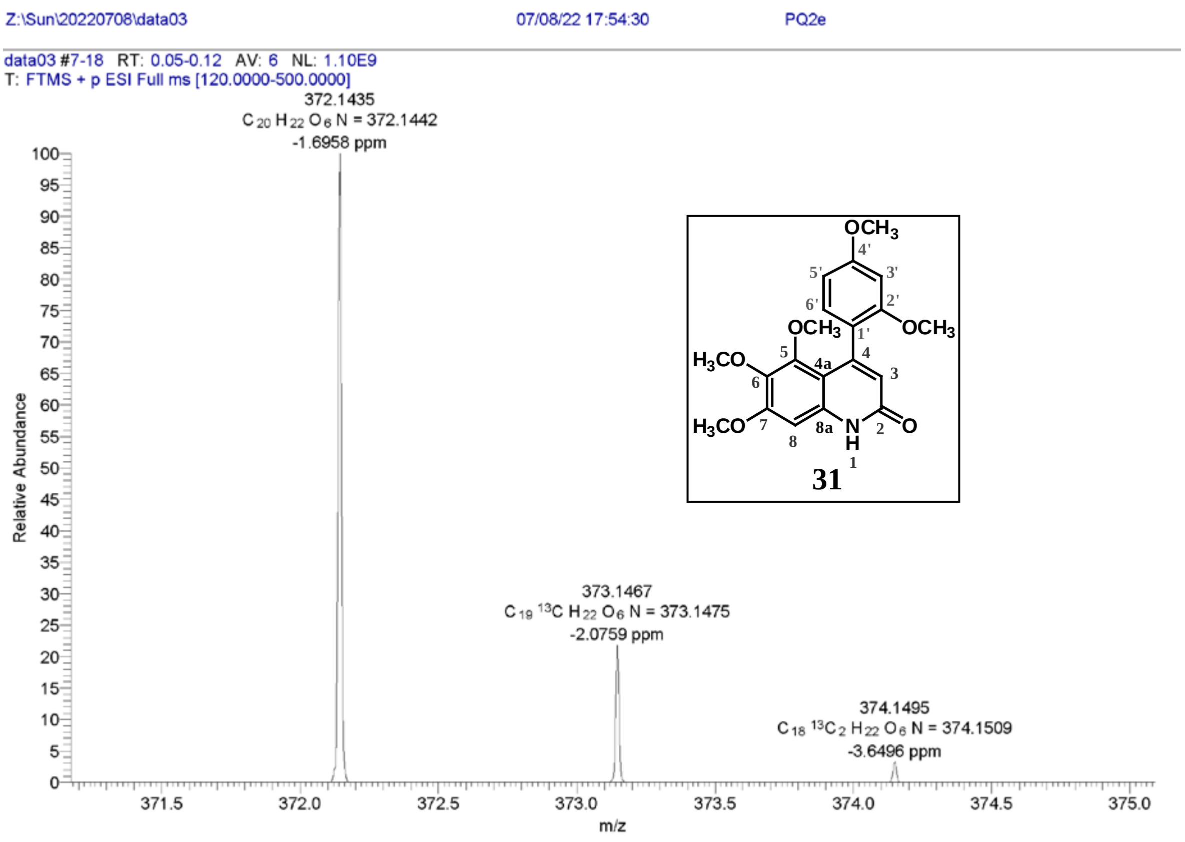


**Figure S83.** Mass Spectra of compound **4-(2,4-Dimethoxyphenyl)-5,6,7-trimethoxyquinolin-2(1*H*)-one (31)**

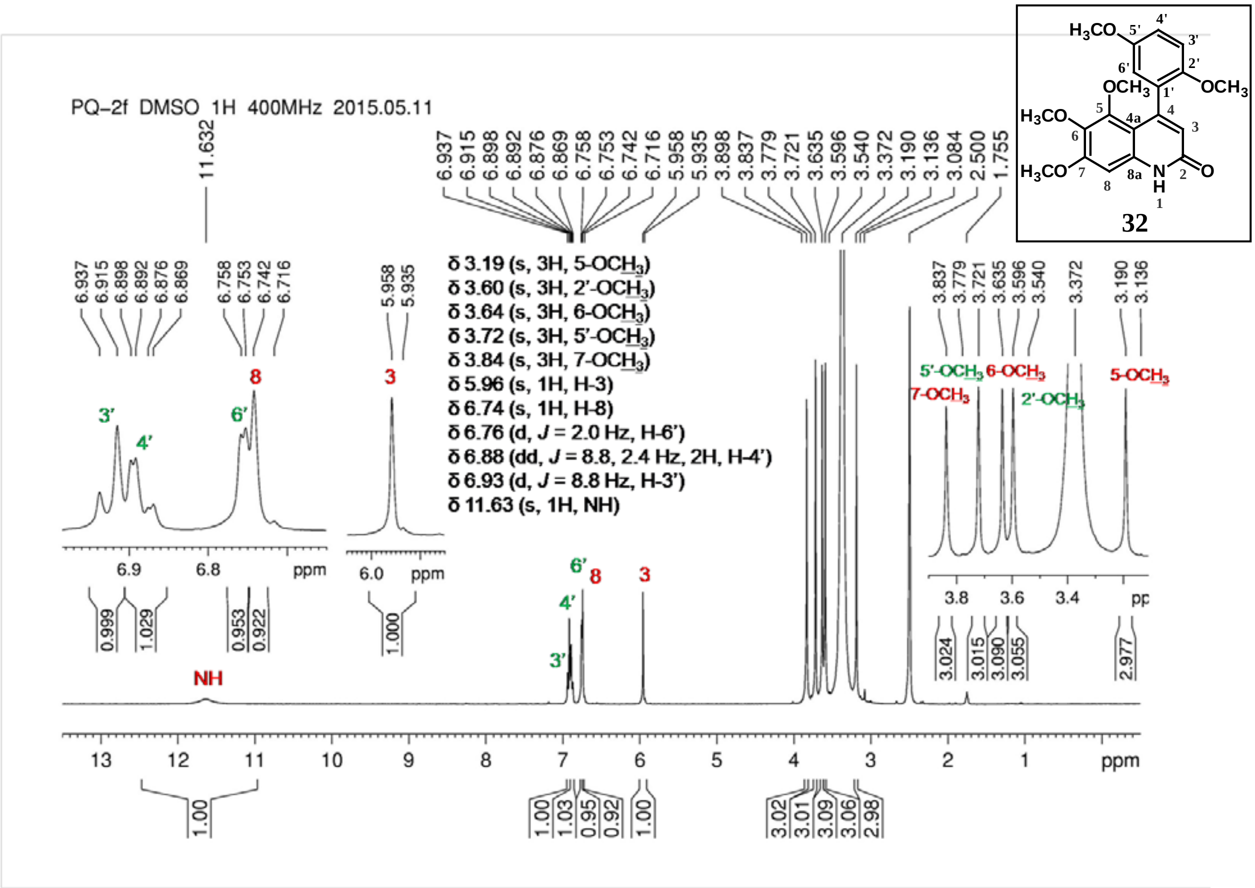


**Figure S84.** $^{1}$H-NMR Spectra of compound **4-(2,5-Dimethoxyphenyl)-5,6,7-trimethoxyquinolin-2(1*H*)-one (32)** was recorded in DMSO-$d_6$ (400MHz)

δ 55.82 (2′-$OCH_3$), 55.96 (5′-$OCH_3$), 56.17 (7-$OCH_3$), 60.88 (6-$OCH_3$), 60.95 (5-$OCH_3$), 94.17 (C-8), 108.09 (C-4a), 111.25 (C-3′), 113.02 (C-4′), 114.79 (C-6′), 120.47 (C-3), 131.52 (C-1′), 136.99 (C-8a), 137.50 (C-6), 148.09 (C-4), 150.59 (C-5′), 150.99 (C-5), 152.85 (C-2′), 155.98 (C-7), 161.56 (C-2).

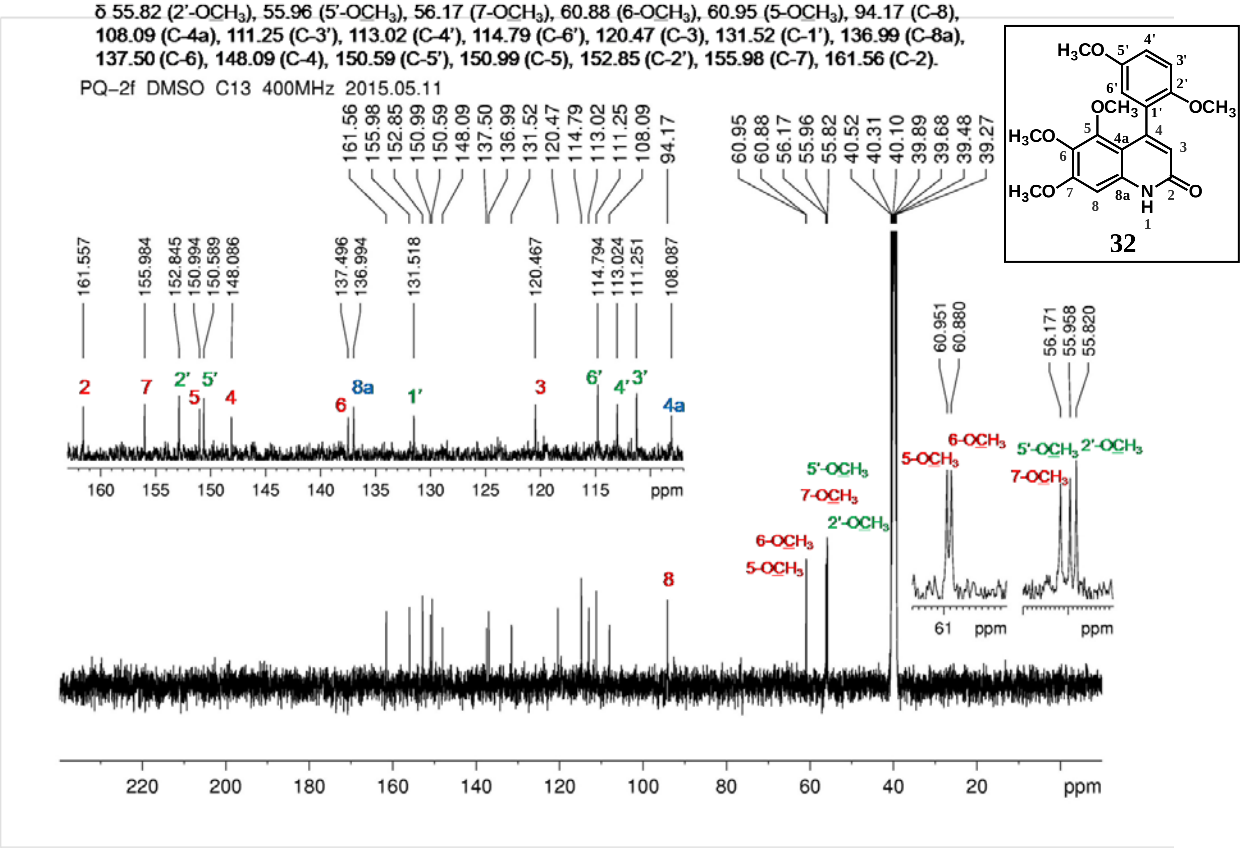


**Figure S85.** $^{13}$C-NMR Spectra of compound **4-(2,5-Dimethoxyphenyl)-5,6,7-trimethoxyquinolin-2(1*H*)-one (32)** was recorded in DMSO-$d_6$ (100MHz)

725, 750, 869, 1024, 1043, 1120, 1215, 1226, 1274, 1357, 1402, 1433, 1492, 1616, 1664 (C=O), 2601-3149 (C-H), 3410 (N-H).

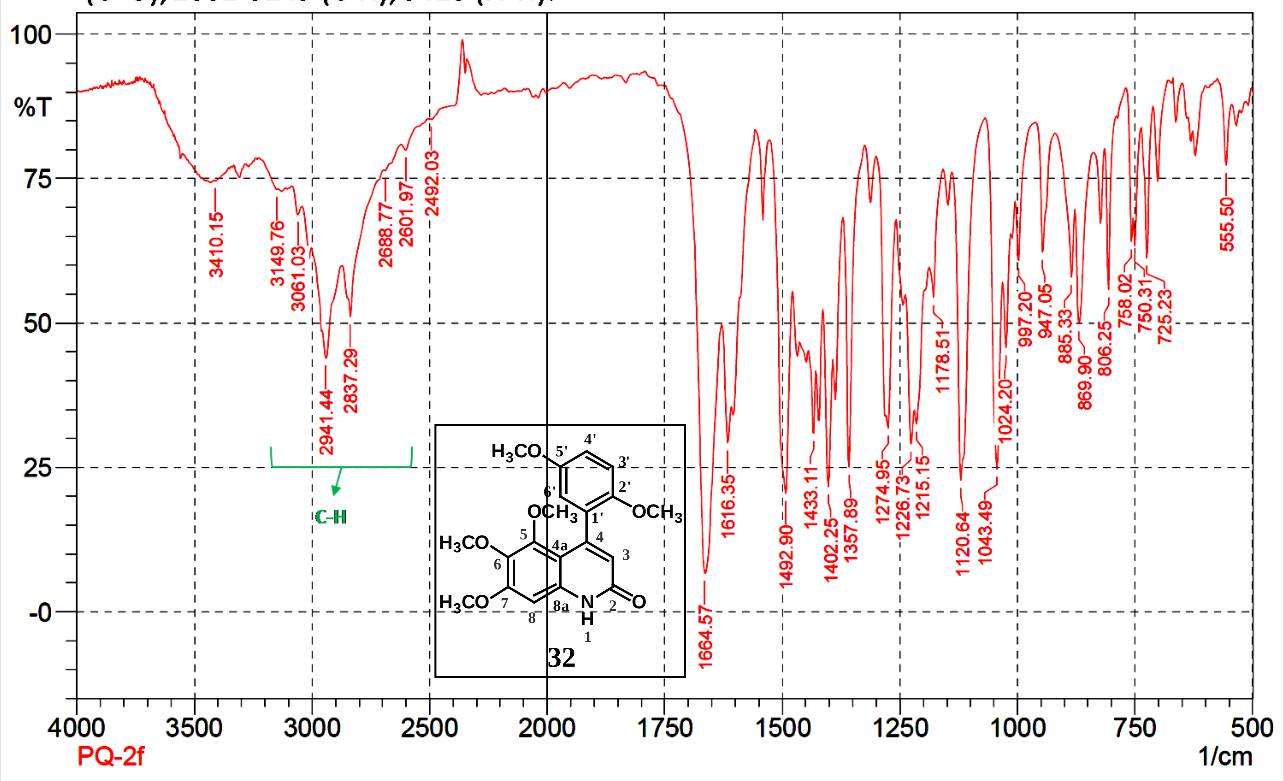


**Figure S86.** IR Spectra of compound **4-(2,5-Dimethoxyphenyl)-5,6,7-trimethoxyquinolin-2(1*H*)-one (32)**

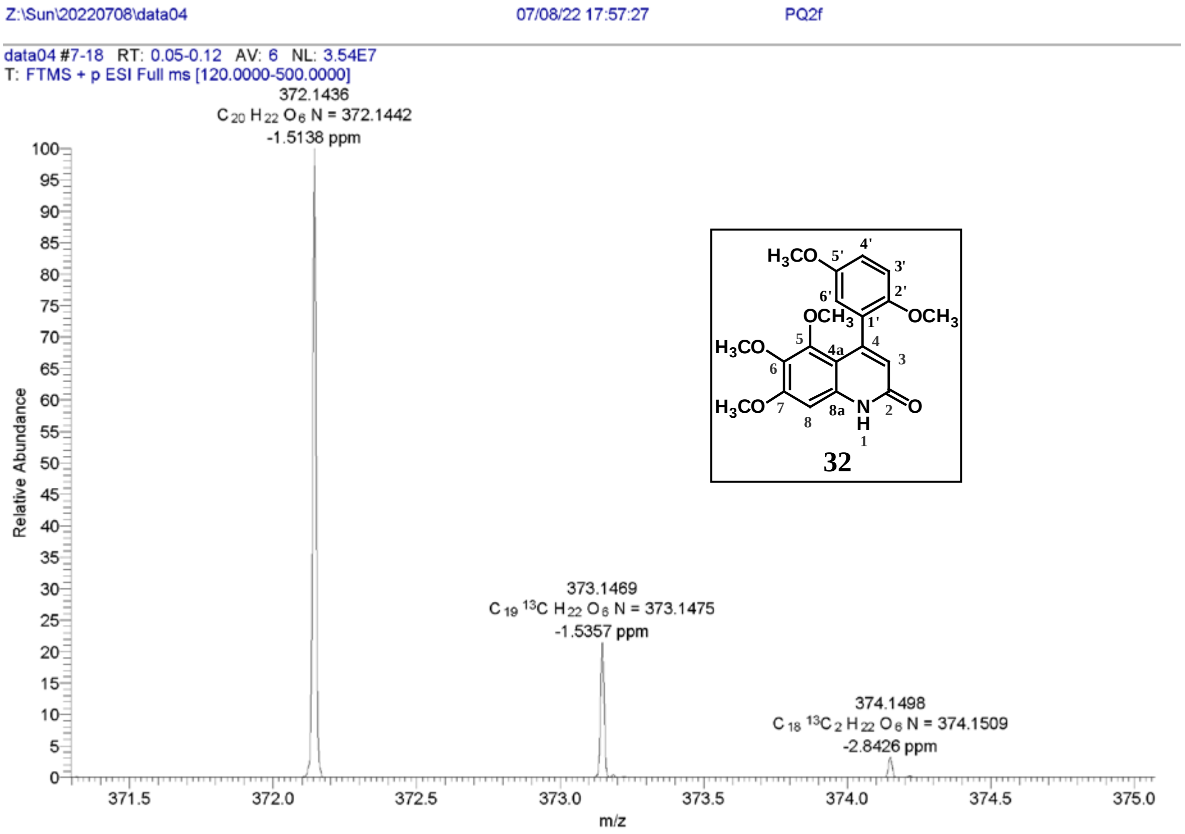


**Figure S87.** IR Spectra of compound **4-(2,5-Dimethoxyphenyl)-5,6,7-trimethoxyquinolin-2(1*H*)-one (32)**
